# Synthesis and biological evaluation of novel carnosic acid derivatives with anticancer activity

**DOI:** 10.1039/d5ra02441b

**Published:** 2025-10-06

**Authors:** Sara P. S. P. Moura, Marta Cascante, Ismael Rufino, Rita C. Guedes, Silvia Marin, Jorge A. R. Salvador

**Affiliations:** a Laboratory of Pharmaceutical Chemistry, Faculty of Pharmacy, University of Coimbra 3000-548 Coimbra Portugal salvador@ci.uc.pt +351-239-488-479; b Center for Neuroscience and Cell Biology (CNC), University of Coimbra 3004-504 Coimbra Portugal; c Centre for Innovative Biomedicine and Biotechnology (CIBB), University of Coimbra 3004-504 Coimbra Portugal; d Department of Biochemistry and Molecular Biomedicine, Faculty of Biology, University of Barcelona 08028 Barcelona Spain silviamarin@ub.edu +34-934-9683; e Centro de Investigación Biomédica en Red de Enfermedades Hepáticas y Digestivas (CIBEREHD), Instituto de Salud Carlos III (ISCIII) 28029 Madrid Spain; f Research Institute for Medicines (iMed.ULisboa), Faculty of Pharmacy, University of Lisbon 1649-003 Lisboa Portugal

## Abstract

Novel derivatives of carnosic acid 1 with ester or carbamate groups at C-20 and derivatives with these functional groups combined with benzylic modifications (C-7) were synthesized and evaluated in a colorectal cancer cell line (HCT116). Compound 8, which featured a butyl ester at C-20 and a carbonyl group at C-7, and compound 17, which featured a 2-methylpropyl carbamate at C-20, achieved the best results in HCT116 cells. Compounds 8 and 17 also demonstrated better ability to inhibit the growth of other cancer cell lines than CA 1. In general, the best results were achieved with compound 17, which exhibited higher potency against SW480 cells (IC_50_ = 6.3 μM). This compound also showed selectivity for cancer cells compared to normal cells. Compound 17 was subjected to additional studies to elucidate the mechanism responsible for its antiproliferative activity in SW480 cells. At 24 h, compound 17 arrested the cell cycle at the G0/G1 phase by decreasing the CDK4/CDK6 levels. It also reduced ROS levels by increasing the expression of SOD2/MnSOD. However, at 48 h, compound 17 induced cell cycle arrest in the S phase and increased ROS levels. At 72 h, compound 17 elevated the ROS levels without inducing cell cycle arrest. Additionally, molecular docking studies showed that compound 17 establishes several interactions with the amino acids of the CDK6 active site. In conclusion, compound 17 is a promising candidate for the development of novel anticancer drugs.

## Abstract 2

Novel derivatives of carnosic acid 1 with ester or carbamate groups at C-20 and derivatives with these functional groups combined with benzylic modifications (C-7) were synthesized and evaluated in a colorectal cancer cell line (HCT116).

## Graphical abstract

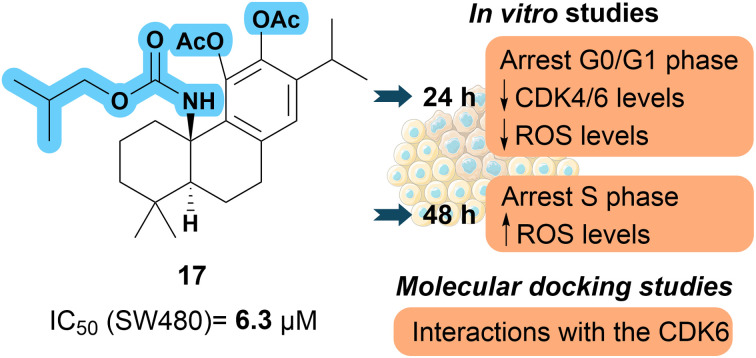

## Introduction

Natural products are a valuable source of bioactive compounds with potential uses in medicine, due to their chemical diversity.^1–3^ Approximately 25% of the anticancer drugs approved between 1981 and 2019 were related to natural products, underlining the significant contribution of these compounds to pharmaceutical advancements in the field.^2^

**Fig. 1:**
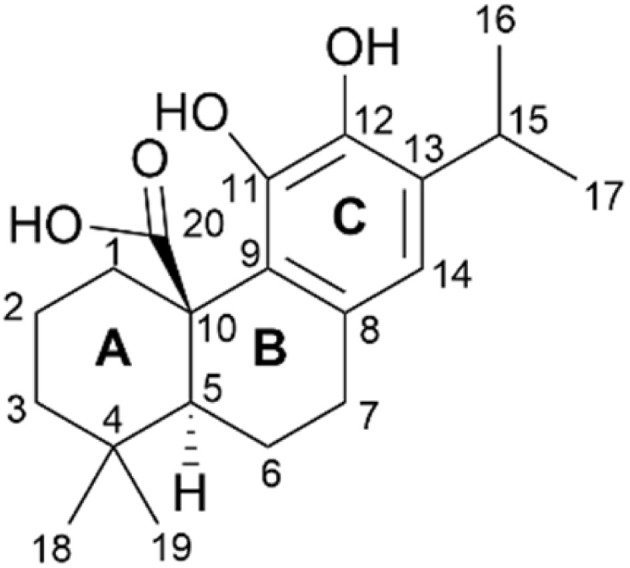
Chemical structure of CA 1.

Abietane-type diterpenoids belong to a family of natural compounds isolated from diverse terrestrial plants.^4,5^ The most abundant group of abietanes found in nature is aromatic abietanes, featuring varying degrees of oxygenation at various positions, an aromatic ring C, and nonfunctionalized A-ring carbons. Carnosic acid (CA) 1, a type of aromatic abietane, is characterized by a tricyclic ring system, a diphenol C-ring, and a carboxylic acid at the C-20 position (Fig. 1). This abietane is found in rosemary leaves (*Rosmarinus officinalis*) and common sage (*Salvia officinalis*). In recent years, numerous studies have highlighted various biological activities of CA 1, such as anticancer, antioxidant, anti-inflammatory, antimicrobial, antiadipogenic, antiangiogenic, antidiabetic, cardioprotective, neuroprotective, and gastroprotective activities.^4,6–13^ Among the biological activities of CA 1, anticancer activity has been the most investigated both *in vitro* and *in vivo* and mechanistic studies have revealed that CA 1 can modulate the biological pathways, such as apoptosis, autophagy, cell cycle and oxidative metabolism, involved in cancer development and progression.^4,6–9^

Although CA 1 has shown promising results in cancer treatment, enhancing its potency, selectivity, and pharmacokinetic properties is essential.^14–16^ One strategy to achieve this goal, minimally explored in this field, involves the chemical modification of the backbone.^5,17–20^ Han *et al.* observed that the anticancer activity of CA 1 against the PANC-1 pancreatic cancer cell line could be enhanced by modifying the carboxylic (C-20) and benzylic (C-7) positions.^17^ This included introducing methyl ester, amides, or substituted 1,3,4-oxadiazole at the C-20 position, as well as introducing substituents using S-linkers or benzylic oxidation at the C-7 position. In another study, it was observed that incorporating an ester group at C-20 along with *o*-quinone or quinone methide in the C-ring improved the anticancer activity of CA 1 against P388 murine leukemia cells.^18^ In a previous study, Moura *et al.* reported that the introduction of a urea group at the C-20 position improved the anticancer activity of CA 1 in HCT116 colorectal cancer cells.^20^ While CA 1 exhibited an IC_50_ of 42 μM, most derivatives showed enhanced potency, with the most active derivatives achieving IC_50_ values between 9.8–14 μM. Carbamate-containing compounds are gaining increasing attention in the field of medicinal chemistry and drug discovery. Some therapeutic agents containing this functional group, such as mitomycin C, irinotecan, and capecitabine, have been used for cancer treatment.^21–23^ The carbamate group enhances chemical stability and permeability across the cell membrane, and modulates interactions with target enzymes or receptors, highlighting its significance in pharmaceutical research.

In this study, we synthesized novel CA 1 derivatives incorporating an ester or a carbamate group at C-20 position, as well as derivatives featuring these functional groups associated with chemical modifications at the benzylic position (C-7). The antiproliferative activity of the CA 1 derivatives was initially evaluated in a colorectal cancer (CRC) cell line (HCT116). Subsequently, the derivatives exhibiting the most promising activity were examined against other cancer cell lines (colorectal, melanoma, and pancreatic). The CA 1 derivative with the best results was chosen for further investigation to elucidate the potential mechanism responsible for the observed anticancer activity.

## Results and discussion

### Chemistry

**Scheme 1:**
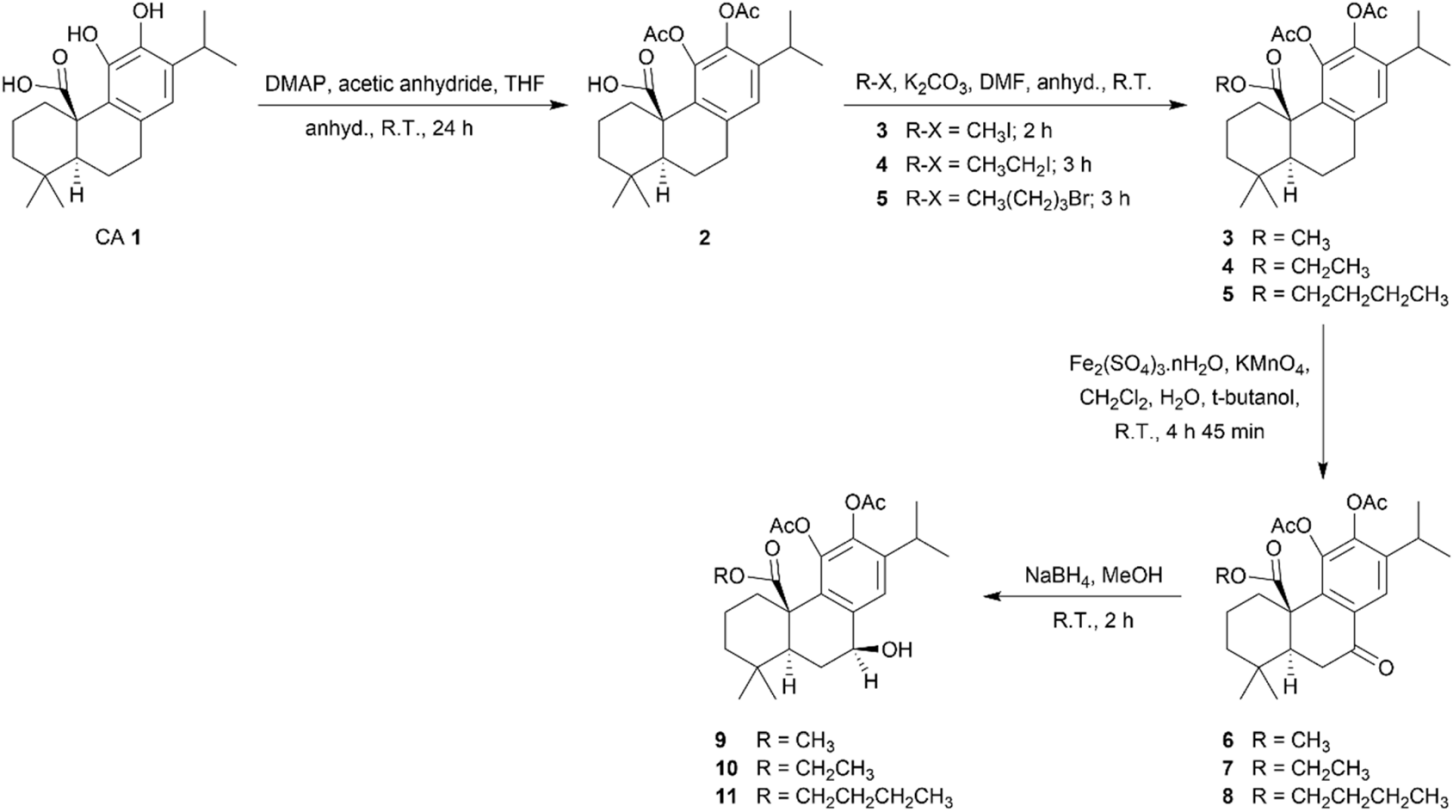
Synthesis of derivatives 2–11 of carnosic acid 1.

**Scheme 2:**
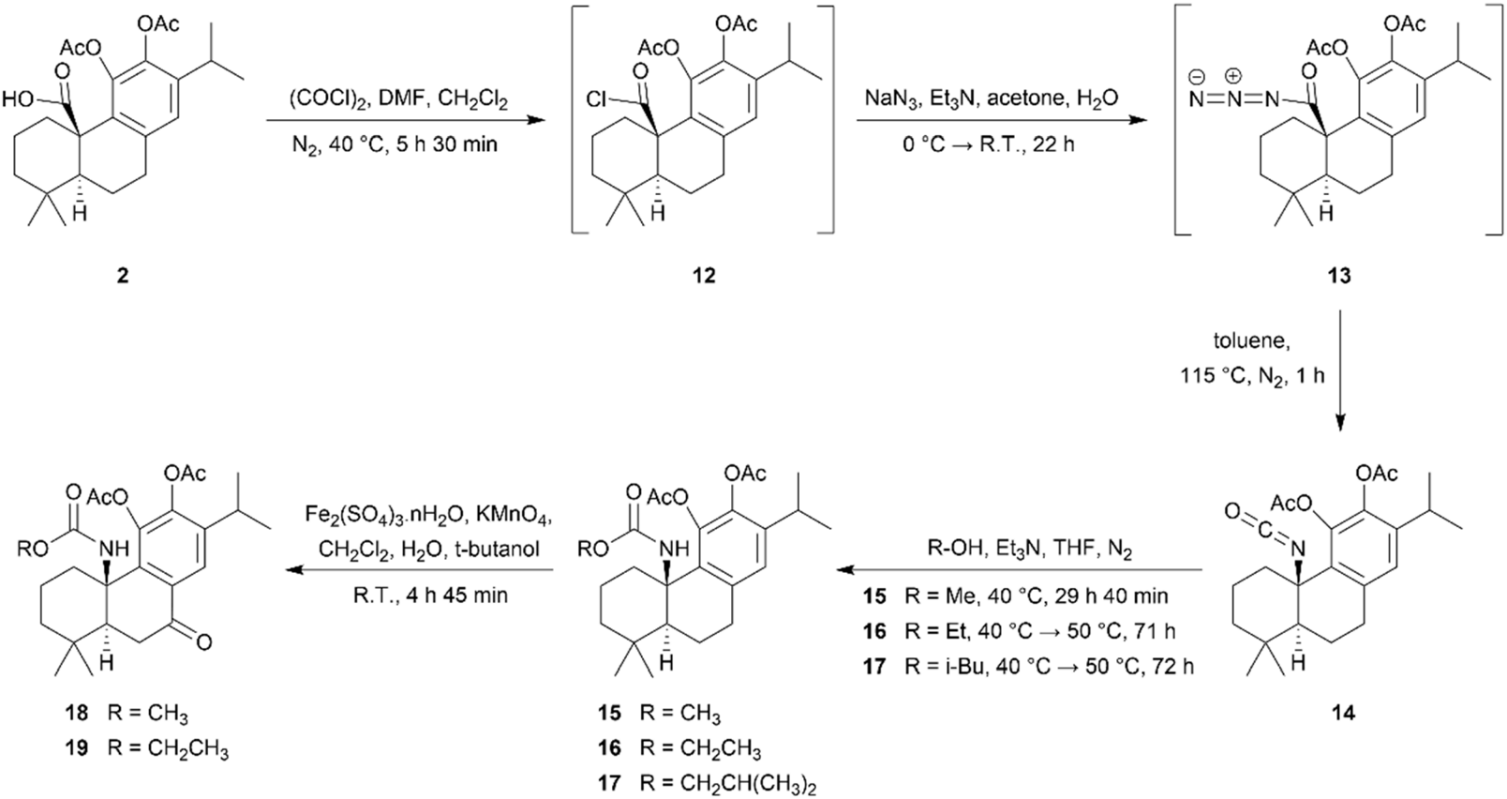
Synthesis of derivatives 14–19 of carnosic acid 1.

The syntheses of CA 1 derivatives with an ester or carbamate group at the C-20 position and derivatives featuring these functional groups, along with chemical modifications at the benzylic position (C-7), are illustrated in Schemes 1 and 2.

In order to control the reactivity of the hydroxyl groups at the C-11 and C-12 positions, CA 1 was treated with 4-dimethylaminopyridine (DMAP) and acetic anhydride to introduce acetate groups at these positions, resulting in derivative 2 (Scheme 1).^20^ This derivative with acetate groups at C-11 and C-12 allows for a more selective reaction at other positions, reducing the production of secondary products, and has already been synthesized by other research groups.^17,18,24^

The carboxylic acid at the C-20 of derivative 2 was esterified using methyl iodide, ethyl iodide, and *N*-butyl bromide, in the presence of anhydrous potassium carbonate, to give derivatives 3–5, respectively (Scheme 1). Compound 3 was previously synthesized by other groups of researchers using other synthetic routes.^17,18,24,25^ The successful incorporation of the ester group into derivatives 3–5 was confirmed by the presence of a peak at approximately 175 ppm in the ^13^C NMR spectra and a band stretch between 1713–1719 cm^−1^ in the IR spectra. A singlet at 3.51 ppm, a triplet at 1.16 and a triplet at 0.80 ppm were observed in the ^1^H NMR spectra of compounds 3–5, respectively, corresponding to methyl groups of the ester chains. In ^13^C NMR spectra of compounds 4 and 5, a peak at approximately 14 ppm was observed for the carbon of the methyl group of the ester chain. Signals of the methylene groups bonded to the oxygen atom of the ester chains in compounds 4 and 5 were observed in the range 3.82–4.06 ppm in the ^1^H NMR spectra, while the corresponding methylene carbons appeared at 60.93 and 64.96 ppm in the ^13^C NMR spectra.

The next step aimed to explore modifications at the benzylic position (C-7) of the ester derivatives through the incorporation of a carbonyl group (derivatives 6–8) and subsequent reduction to a hydroxyl group (derivatives 9–11), as depicted in Scheme 1. To the best of our knowledge, among the derivatives 6–11, only compound 6 was previously synthesized by another research group through an alternative synthetic route employing chromium trioxide in an acetic acid solution.^17^ Derivatives 3–5 underwent oxidation at C-7 by reacting with a mixture of iron sulfate and potassium permanganate to give derivatives 6–8, respectively (Scheme 1). Benzylic oxidation was confirmed by the presence of a peak at approximately 198 ppm in the ^13^C spectra and a band stretch at approximately 1690 cm^−1^ in the IR spectra of the derivatives 6–8. After, the derivatives 6–8 underwent reduction of the carbonyl group at C-7 through reaction with sodium borohydride in anhydrous methanol, resulting in the formation of derivatives 9–11, respectively (Scheme 1). The ^1^H NMR spectra of these derivatives revealed a signal around 4.74 ppm corresponding to the benzylic proton (7-H), while the ^13^C NMR spectra exhibited a peak around 71 ppm, attributed to the benzylic carbon linked to the hydroxyl group. Additionally, successful reduction was also confirmed by the presence of a broad O–H stretching vibration between 3503 and 3516 cm^−1^. This reduction generated a new stereogenic center at C-7. Analysis of the NOESY spectrum of compound 10 revealed a spatial correlation between the proton at C-5 (5-H, α-oriented) and the benzylic proton (7-H), allowing the stereochemical assignment of 7-H as α-oriented. Given the structural similarity and identical reaction conditions, this stereochemical configuration can be extended to compounds 9 and 11.

To synthesize the novel carbamate-containing derivatives CA 1, we initially focused on preparing an isocyanate-containing derivative at C-20 (derivative 14) from derivative 2. This derivative was previously synthesized by our research group.^20^ After, derivative 14 underwent a reaction with some alcohols in THF solution at 40/50 °C, resulting in the formation of carbamate-containing derivatives (15–17), as depicted in Scheme 2. The successful introduction of the carbamate moiety was verified by NMR and IR techniques. In the ^1^H NMR spectra, the proton signal of the –NH group appeared as a singlet in the 4.72–4.76 ppm range. In the IR spectra, a band was observed in the 1513–1515 cm^−1^ range, which resulted from a combination of an N–H bending band and a C–N stretching band, and another band was observed in the 3437–3447 cm^−1^ range relative to the N–H bond stretch. In the ^13^C NMR spectra, the signal of the carbamate carbonyl group appeared at approximately 154 ppm, and an IR band corresponding to the C=O bond stretch was observed in the characteristic region of 1729–1735 cm^−1^. In the ^1^H NMR spectra of derivatives 15–17, the signals referent to the methyl groups of the carbamate chains appeared as a singlet at 3.50 ppm for compound 15, a multiplet at 1.18–1.11 ppm for compound 16, and a doublet at 0.85 ppm for compound 17. The signals of the methylene groups bonded to the oxygen atom in compounds 16 and 17 were observed between 3.53 at 3.99 ppm. In the ^13^C NMR spectra, the signals of the carbons of the methylene groups appeared at 60.31 and 70.61 ppm.

Similar to the approach used for the ester derivatives, we synthesized carbamate derivatives containing a carbonyl group at the benzylic position (C-7) (derivatives 18 and 19), as illustrated in Scheme 2. To achieve this, derivatives 15 and 16 were treated under the aforementioned reaction conditions, resulting in the formation of derivatives 18 and 19, respectively. Successful benzylic oxidation was confirmed by the signal at approximately 197 ppm in the ^13^C spectra.

### Biology

#### Determination of the cell viability

**Fig. 2:**
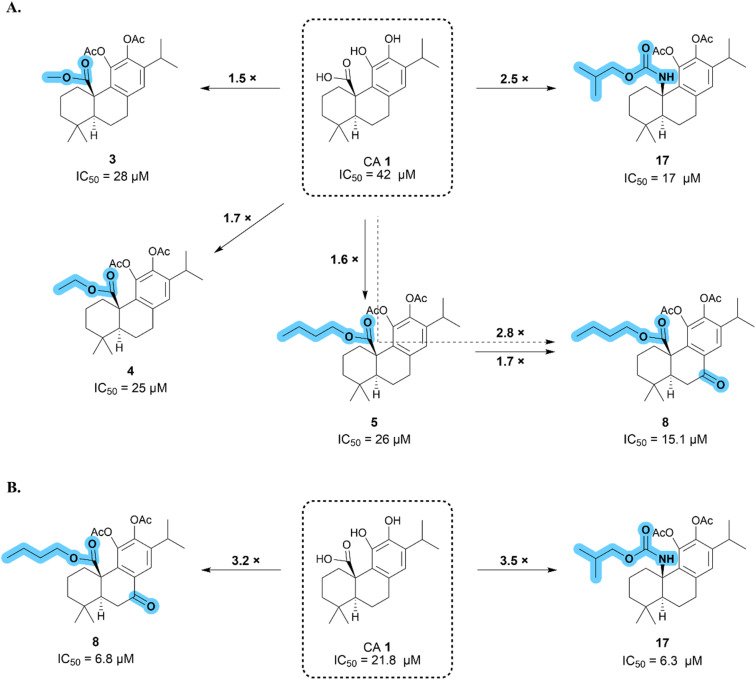
Schematic representation of the main SAR conclusions obtained from the IC_50_ values of the CA 1 derivatives in HCT116 (A) and SW480 (B) cells.

The influence of CA 1 and its derivatives (2–11 and 14–19) on cell viability was assessed in a CRC cell line (HCT116), using the MTT assay after 72 h of treatment. Cisplatin was used as the reference anticancer drug because it is a well-characterized drug employed in the treatment of several cancer types. The obtained results allowed the determination of the concentration necessary to inhibit half of the cell growth (IC_50_) (Table 1). The IC_50_ values were used to create a structure–activity relationship (SAR) between the derivatives, and the main SAR conclusions are summarized in Fig. 2A.

**Table 1:** IC_50_ values of CA 1, its derivatives (2–11 and 14–19) and cisplatin against on CRC cell line (HCT116)^a^

Compound	Cell line/IC_50_^b^ (μM)
	HCT116
CA 1	42 ± 4
2	44 ± 4
3	28 ± 2
4	25 ± 2
5	26 ± 1
6	36 ± 1
7	31 ± 3
8	15.1 ± 0.7
9	37 ± 3
10	34 ± 3
11	27 ± 4
14	28 ± 3
15	55 ± 5
16	37 ± 2
17	17 ± 2
18	>50
19	48.2 ± 0.1
Cisplatin	21 ± 1 (ref. 26)^c^

a After 72 h of incubation with increasing concentrations of each compound, IC_50_ values were calculated based on the results of cell viability obtained using the MTT assay. IC_50_ values are relative to at least three independent experiments and are expressed as the mean ± SD.

b IC_50_ is the compound concentration necessary to inhibit half of the cell growth.

c IC_50_ value was previously determined by our research team using the same method.

As presented in Table 1, compounds featuring an ester group at the C-20 position (compounds 3–5) demonstrated enhanced antiproliferative activity compared to CA 1. In fact, there was a decrease in the IC_50_ values from 42 μM (CA 1) to 28 (3), 25 (4), and 26 μM (5). The length of the ester chain did not seem to influence the potency of the compounds, revealing very similar potency.

In general, modifications at the benzylic position (C-7) in the ester derivatives, namely, the introduction of a carbonyl group (compounds 6–8) or hydroxyl group (compounds 9–11), led to a reduction in antiproliferative activity compared to ester derivatives without modifications at this position (compounds 3–5), as shown in Table 1. However, compound 8 was the only exception, exhibiting an increase in potency, verifying a reduction in the IC_50_ value from 26 μM (derivative 5) to 15.1 μM (compound 8). Consequently, it can be inferred that the insertion of a carbonyl group at the benzylic position (C-7) was advantageous for the antiproliferative activity of the butyl ester derivative.

In compounds featuring a carbamate moiety at the C-20 position (compounds 15–17), carbamate chain length influenced their antiproliferative activity (Table 1). Carbamate 15, which possesses a methyl chain, exhibited a decrease in activity with an IC_50_ value (55 μM) higher than that of CA 1 (42 μM). With an increase in chain length, a simultaneous increase in the potency of the compounds was observed. Compound 16, characterized by an ethyl chain, exhibited an IC_50_ of 37 μM, whereas compound 17, featuring a longer chain (2-methylpropyl), demonstrated even greater potency with an IC_50_ of 17 μM. Benzylic oxidation of compounds 15 and 16, resulting in the formation of compounds 18 and 19, did not contribute to improved antiproliferative activity (Table 1).

Compound 8, featuring a butyl ester at C-20 and a carbonyl group at benzylic position (C-7), and compound 17, featuring a 2-methylpropyl carbamate, exhibited the most potent antiproliferative activity in HCT116 cells, with IC_50_ values of 15.1 μM and 17 μM, respectively. These compounds achieved better results than cisplatin, being 1.4- and 1.2-fold more active, respectively. Therefore, compounds 8 and 17 were selected for further evaluation in other cancer cell lines. Different cell lines of the same cancer type carry different genetic mutations, leading to variations in their susceptibility to the same treatment.^27,28^ Given that the HCT116 cell line is wild-type for p53 and microsatellite unstable, our aim was to investigate the effects of compounds 8 and 17 on CRC cell lines with mutations in the p53 gene and microsatellite stable, such as SW480, SW620, and Caco-2 cells.^27^ Furthermore, we selected SW480 and SW620 cells because they were derived from the same tumor, but from different locations. We investigated whether the compounds tended to exhibit greater effectiveness in a cell line derived from the primary tumor (SW480) or on a cell line derived from metastasis (SW620). As displayed in Table 2, compounds 8 and 17 demonstrated higher potency against SW480, SW620, and Caco-2 cells than CA 1. Moreover, they achieved better IC_50_ values in these cell lines than in HCT116 cells, showing a preference for p53-mutated cell lines, particularly SW480 cells. In this line, compound 8 and 17 achieved IC_50_ values of 6.8 μM and 6.3 μM, respectively, being 3.2-fold and 3.5-fold more potent than CA 1 (Fig. 2B). These two compounds also showed better antiproliferative activity against SW480 cells than cisplatin, being 2.2-fold (compound 8) and 2.4-fold (compound 17) more active. Comparing the results obtained in SW480 and SW620 cells, it is evident that the compounds exhibited greater effectiveness against the primary tumor cells. Further investigations were conducted using compounds 8 and 17 to assess their capacity to inhibit cell growth in other cancer types, specifically pancreatic cancer, and melanoma. As shown in Table 2, it can be inferred that these compounds are also effective against other cancer types.

**Table 2:** IC_50_ values of CA 1, its derivatives (8 and 17) and cisplatin against colorectal, pancreas, and melanoma cancer cell lines and the non-tumoral cell line (BJ)^a^

Compound	Cell line/IC_50_^b^ (μM)					
	Colorectal			Pancreas	Melanoma	Non-tumoral
	SW480	SW620	Caco-2	Mia Paca-2	A375	BJ
CA 1	21.8 ± 0.7	18 ± 2	34 ± 2	21 ± 1	27.6 ± 0.5	N.D.
8	6.8 ± 0.6	11 ± 1	12.7 ± 0.3	12 ± 1	8.1 ± 0.5	N.D.
17	6.3 ± 0.5	10 ± 1	16 ± 2	7.5 ± 0.6	6.7 ± 0.5	>50
Cisplatin	15.2 ± 0.4 (ref. 34)^c^	1.4 ± 0.5 (ref. 35)^d^	12.5 ± 0.9 (ref. 36)^c^	5 ± 1 (ref. 37)^c^	3 ± 1 (ref. 38)^c^	10 ± 2 (ref. 39)^d^

a After 72 h of incubation with increasing concentrations of each compound, IC_50_ values were calculated based on the results of cell viability obtained using the MTT assay. IC_50_ values are relative to at least three independent experiments and are expressed as the mean ± SD. N.D. – not determined.

b IC_50_ is the compound concentration necessary to inhibit half of the cell growth.

c IC_50_ value was acquired from the literature by applying the same method.

d IC_50_ value was previously determined by our research team using the same method.

**Fig. 3:**
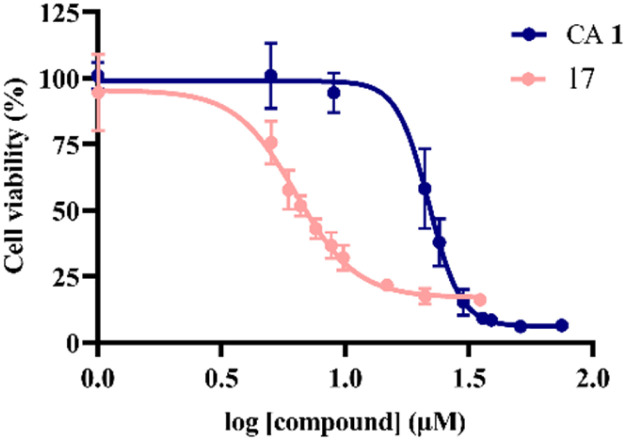
Dose–response curves of CA 1 (IC_50_ = 21.8 μM) and compound 17 (IC_50_ = 6.3 μM) in SW480 cells after 72 h of treatment. Cell viability was determined using the MTT assay, with the results reported as the mean ± SD obtained from at least three independent experiments.

In general, compound 17 achieved the most promising results against almost all cancer cell lines tested, and achieved the best results for SW480 cells, with an IC_50_ of 6.3 μM (Table 2 and Fig. 3). Therefore, we evaluated this compound in a non-tumoral cell line (BJ fibroblasts) to determine its selectivity for cancer cell lines. As indicated in Table 2, the antiproliferative activity of compound 17 notably decreased in BJ cells, being at least 3–8 times less potent than that in cancer cell lines. These findings highlight the selectivity of compound 17 in cancer cells, showing even greater selectivity than cisplatin. Hence, considering the acquired results, compound 17 was selected for additional biological studies in SW480 cells to gain insight into the mechanisms underlying its anticancer activity.

#### Effect of compound 17 on cell growth rate

**Fig. 4:**
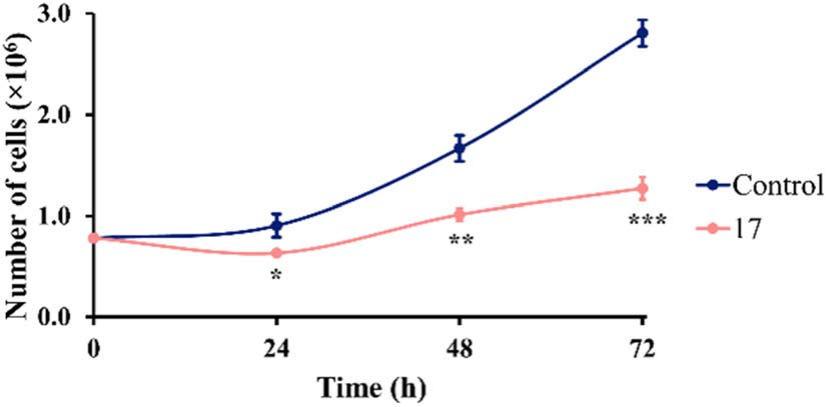
Time-dependent growth curves of control cells and cells treated with compound 17 at 6.3 μM (IC_50_ value). At each time point (0, 24, 48, and 72 h), the cells were collected, and the number of viable cells was counted. Data are relative to three independent experiments (mean ± SD). Multiple *t*-tests (one independent *t*-test per row) were used to assess differences between the control and treatment groups. Statistical significance was defined as *p* < 0.05 (*), *p* < 0.01 (**), or *p* < 0.001 (***).

Cell growth rates represent a characteristic of cell lines, indicating the rate at which the cell population size changes over time.^29^ The commonly used metric for assessing the cell growth rate is the cell doubling time, which denotes the time required for a cell population to double in size. Cell doubling time can be estimated using the following formula: ln(2)/cell growth rate.^30^ A time-dependent cell growth curve was constructed to elucidate the effect of compound 17 on the growth pattern of SW480 cells (Fig. 4). As shown in Fig. 4, SW480 cells treated with compound 17 exhibited decelerated growth over time compared to untreated cells. The cell concentration at each time point allowed us to determine the cell growth rate and subsequently calculate the cell doubling time. Compound 17 induced an increase in the doubling time of SW480 cells (69 h) compared to the control group (43 h). In summary, compound 17 affected the growth pattern of SW480 cells by increasing the cell doubling time, consequently decreasing the cell growth rate.

#### Evaluation of apoptosis in SW480 cells

The induction of apoptosis is a mechanism used by some anticancer drugs to kill cancer cells and control their proliferation, progression, and spread.^31,32^ Detection and quantification of apoptotic cells can be achieved using propidium iodide (PI) and annexin V staining.^33^ The live cells are annexin V-negative and PI-negative. In early apoptotic cells, phosphatidylserine (PS) is exposed on the extracellular side, leading to strong binding of annexin V to PS. However, the nuclear membrane remains intact, and PI cannot bind to DNA. In late apoptotic/necrotic cells, annexin V binds to PS and alterations in the nuclear membrane allow PI to bind to DNA.

**Fig. 5:**
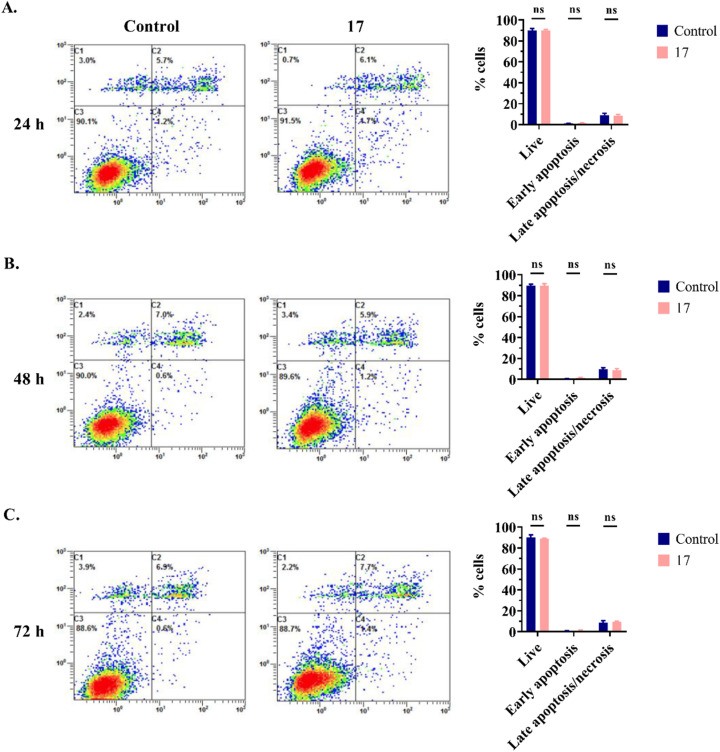
Analysis of apoptosis in SW480 cells for 24 h (A), 48 h (B) and 72 h (C). Untreated cells (control) or cells treated with compound 17 at 6.3 μM (IC_50_ value) were stained with annexin V–FITC and PI and analyzed by flow cytometry. Each histogram is divided into four quadrants: the lower left quadrant represents the percentage of live cells, the lower right quadrant represents the early apoptotic cells, and the upper quadrants (right and left) represent the late apoptotic/necrotic cells. The graphs illustrate the percentage of live cells, early apoptotic cells, and late apoptotic/necrotic cells. Data from three independent experiments are presented as the mean ± SD. Multiple *t*-tests (one independent *t*-test per row) were used to assess differences between the control and treatment groups. ns, statistically non-significant.

Our objective was to assess whether the anticancer activity observed in SW480 cells treated with compound 17 results from the activation of apoptosis. Therefore, SW480 cells were treated with 6.3 μM (IC_50_ value) of compound 17, incubated for 24, 48 or 72 h, and subsequently stained with annexin V and PI for post-analysis using flow cytometry. As shown in Fig. 5, compound 17 did not induce apoptosis in the SW480 cells, suggesting that this mechanism is not responsible for the anticancer activity of compound 17.

#### Evaluation of the cell cycle in SW480 cells

The cell cycle is a series of events that coordinate cell proliferation.^40,41^ Control of the cell cycle is essential for ensuring that cells only divide when necessary and when DNA replication occurs in an adequate manner.^41^ Cancer is characterized by uncontrolled proliferation due to the disruption of cell cycle control mechanisms.^41,42^ Hence, the development of drugs capable of regulating the cell cycle and reducing uncontrolled cell growth through cell cycle arrest is a promising strategy for combating cancer.^40–42^

**Fig. 6:**
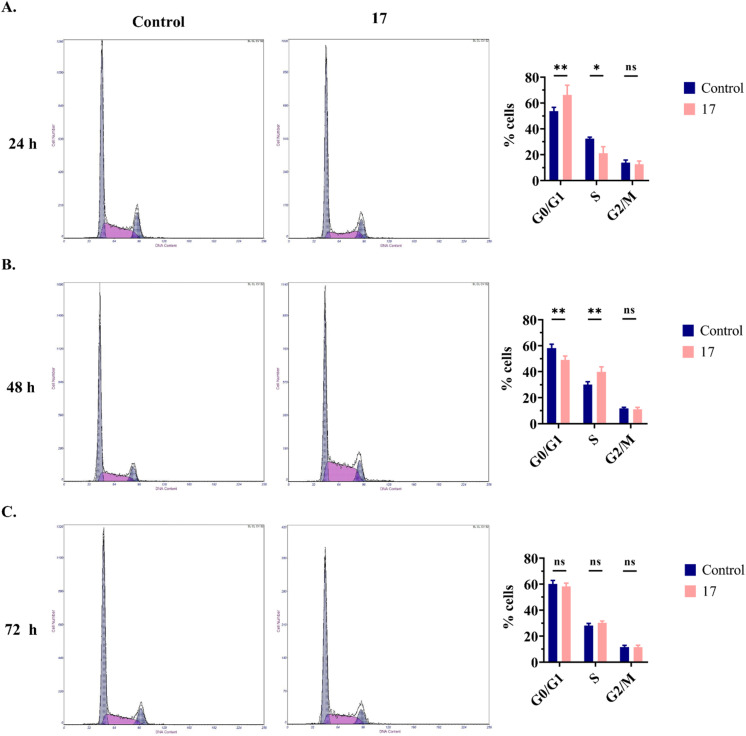
Evaluation of the cell cycle in SW480 cells after 24 h (A), 48 h (B) and 72 h (C) h. At each time point, untreated cells (control) or cells treated with compound 17 at 6.3 μM (IC_50_ value) were fixed and permeabilized with 70% cold ethanol. Cells were then stained with PI and analyzed by flow cytometry to evaluate DNA content. Each histogram demonstrates the number of SW480 cells in the G0/G1, S, and G2/M cell cycle phases. The graphs illustrate the percentage of SW480 cells in different cell cycle phases (G0/G1, S, and G2/M). Data from three independent experiments are presented as the mean ± SD. Two-way ANOVA (Sidak's *post hoc* comparison) was used to assess differences between the control and treatment groups. Statistical significance was defined as *p* < 0.05 (*) and *p* < 0.01 (**). ns, statistically non-significant.

Since compound 17 did not induce apoptosis but increased the duplication time of treated cells, we aimed to evaluate whether its anticancer activity was due to cell cycle arrest. To validate this hypothesis, compound 17 was administered at 6.3 μM to SW480 cells and incubated for 24, 48, and 72 h. Following each incubation period, the cells were fixed and permeabilized with 70% cold ethanol and stained with PI before flow cytometry analysis. At 24 h, compound 17 caused cell cycle arrest at the G0/G1 phase, as evidenced by a significant increase in the percentage of SW480 cells, from 54% in the control group to 66% in the treated group (Fig. 6). However, at 48 h, compound 17 induced cell cycle arrest in the S phase, as demonstrated by a significant increase in the percentage of cells from 30% in the control group to 40% in the treated group (Fig. 6). At 72 h, compound 17 had no impact on the cell cycle of SW480 cells, as observed in Fig. 6.

In conclusion, the effect of compound 17 on the cell cycle of SW480 cells varied over time, arresting the cell cycle in the G0/G1 phase at 24 h and in the S phase at 48 h. These results suggest that the cytostatic effect of compound 17 was responsible for its anticancer activity.

#### Evaluation of protein expression related to cell cycle regulation

The cell cycle is tightly regulated by a set of serine/threonine kinases designed for cyclin-dependent kinases (CDK), cyclins, and CDK inhibitors (CKI).^43–45^ CDKs form complexes with cyclins and orchestrate cell cycle progression by phosphorylating their target proteins. For example, CDK4/CDK6–cyclin D complexes drive the progression of the G1 phase by initiating phosphorylation of the retinoblastoma (Rb) protein. In the late G1 phase, the CDK2–cyclin E complex also phosphorylates Rb protein, surpassing the restriction point between the G1/S phases. This leads to the initiation of the S phase. During the S phase, CDK2 forms a new complex with cyclin A, inducing DNA synthesis and replication, leading to a transition from the S phase to the G2 phase.

**Fig. 7:**
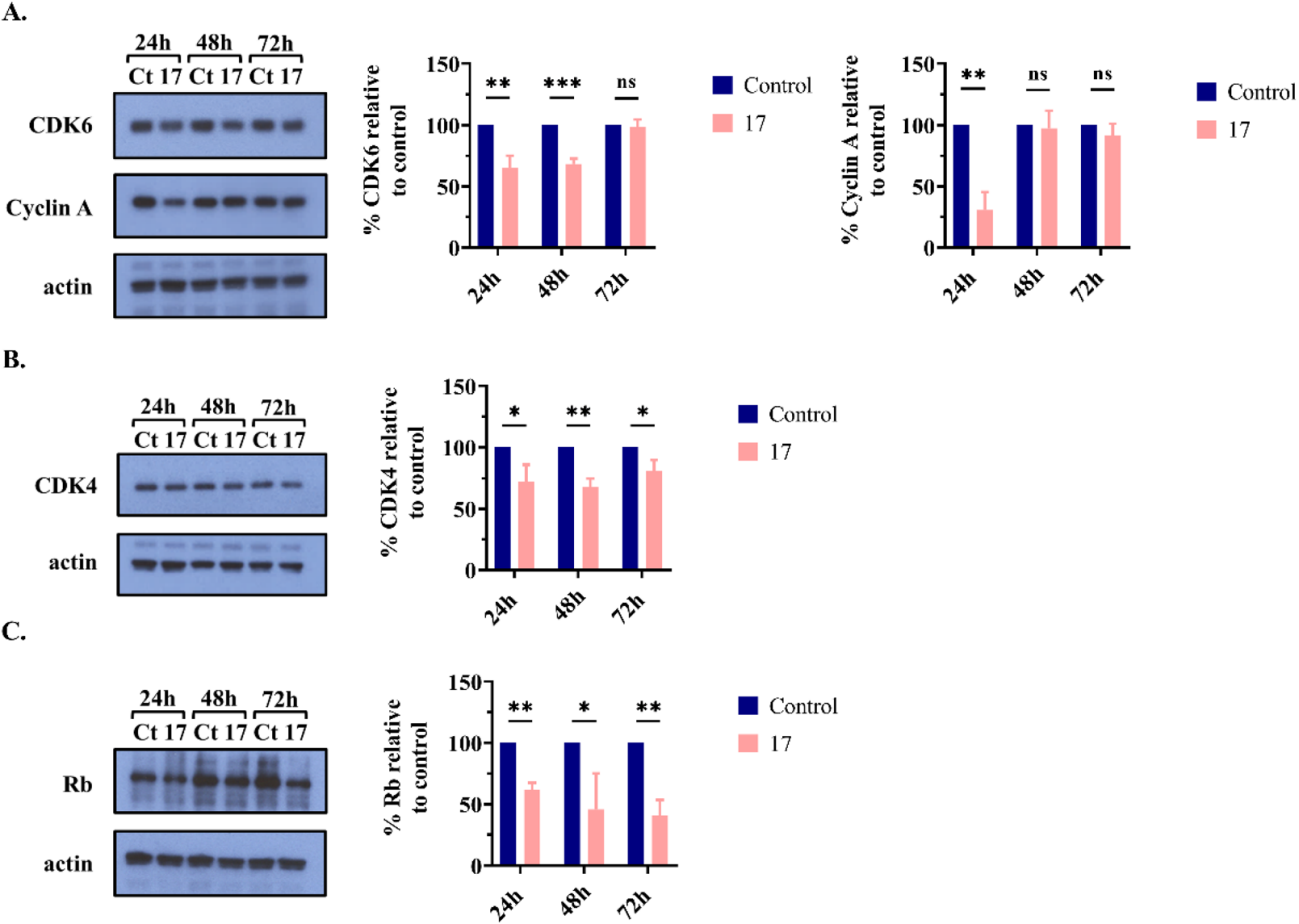
Analysis of protein levels associated with cell cycle regulation using western blot: (A) CDK6 and cyclin A; (B) CDK4; (C) Rb. Untreated cells (control) and cells treated with compound 17 at 6.3 μM (IC_50_ value) were incubated for 24, 48, and 72 h. Actin served as the loading control. The graphs show the percentage of the indicated proteins relative to the control group. Data are presented as the mean ± SD from at three independent experiments, except for cyclin A at 24 h (*n* = 2) and Rb protein at 24 and 72 h (*n* = 2). Multiple *t*-tests (one independent *t*-test per row) were used to assess differences between the control and treatment groups. Statistical significance was defined as *p* < 0.05 (*), *p* < 0.01 (**), or *p* < 0.001 (***). ns, statistically non-significant.

In the above studies, compound 17 induced cell cycle arrest in the G0/G1 and S phases in SW480 cells at 24 and 48 h, respectively. To better understand the mechanism underlying this cell cycle arrest, we analyzed the expression of key proteins associated with cell cycle regulation using western blot (Fig. 7). As shown in Fig. 7, at 24 h, compound 17 significantly reduced the levels of CDK4/6 proteins, explaining the observed cell cycle arrest at the G0/G1 phase.^46–48^ Additionally, a decrease in cyclin A expression was noted at 24 h, possibly due to a reduction in the number of cells in the S phase (Fig. 6). At 48 h, the CDK4/6 proteins levels in SW480 cells treated with compound 17 persisted lower than those in the control group (Fig. 7). This reduction may be attributed to a decrease in the number of cells in the G0/G1 phase (Fig. 6). Surprisingly, at 48 h, compound 17 did not affect the expression level of cyclin A, as shown in Fig. 7. Despite the cell cycle analysis indicating arrest in the S phase at this time point (Fig. 6), cyclin A, which is responsible for S phase progression, did not exhibit a decrease. The arrest of the cell cycle at the S phase may be due to a reduction in the expression of cyclin E, as reported in other studies.^49–51^ Moreover, a notable decrease in the Rb protein levels was observed in SW480 cells treated with compound 17 at three different time points (24, 48, and 72 h), as observed in Fig. 7. These findings are consistent with those of other studies that have reported a decrease in Rb protein levels linked to cell cycle arrest.^52,53^ As previous studies have reported no cell cycle changes at 72 h, we did not expect variations in the expression of cell cycle regulatory proteins. However, a reduction in CDK4 and Rb levels was detected after 72 h of treatment with compound 17. It is possible that these alterations may not have reached a magnitude significant enough to induce cell cycle arrest.

In conclusion, compound 17 induced changes in the levels of cell cycle regulatory proteins, explaining the observed cell cycle arrest in the SW480 cells. The G0/G1 phase arrest observed at 24 h was attributed to a decrease in the CDK4/6 protein levels. Additional studies are essential to elucidate the mechanism by which compound 17 arrests the cell cycle during the S phase. A decrease in Rb protein levels also contributed to cell cycle arrest.

#### Molecular docking studies: interaction of compound 17 with CDK6

CDK4/6 inhibitors have emerged as targeted therapies for cancer treatment.^54^ These molecules inhibit the activity of CDK4/6, leading to G1 cell cycle arrest and suppression of cell proliferation. Currently, three CDK4/CDK6 inhibitors – palbociclib, ribociclib, and abemaciclib – are used in clinical practice to treat hormone receptor (HR)-positive breast cancer. Additionally, there has been increasing interest in developing CDK4/CDK6 inhibitors for a broader range of cancer types.

**Fig. 8:**
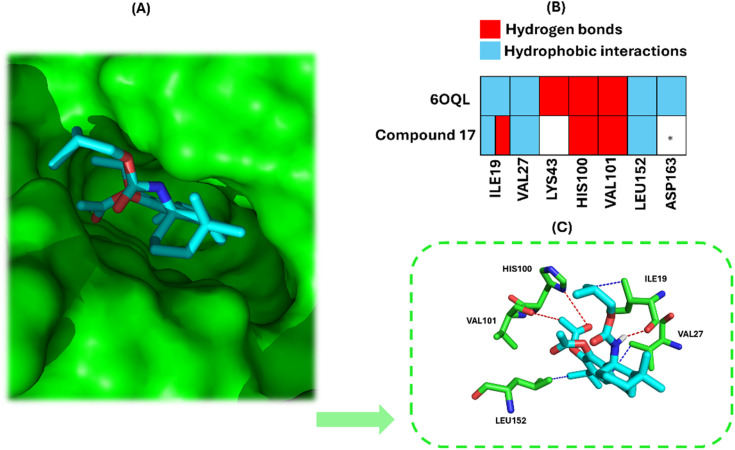
Docking studies of compound 17 into the active site of CDK6 (PDB ID 6OQL), as predicted by the GNINA 1.0 docking software. Panel (A) illustrates the optimal docking pose of compound 17, highlighted in cyan, within the CDK6 binding site, with the CDK6 surface highlighted in green. Panel (B) compares the interactions observed in the 6OQL structure with those established by compound 17, noting that five out of seven interactions involve identical amino acid residues. Additionally, an extra hydrogen bond interaction is formed by ILE19. Panel (C) provides a detailed view of the ligand–protein interactions between compound 17 and the amino acid residues within the CDK6 binding pocket. For clarity, only the five residues that simultaneously interact with both compound 17 and the native ligand in the 6OQL structure are shown. *Compound 17 interacts with the amino acid residue ALA162, which is very close to ASP163.

Given that compound 17 induced G1 phase arrest at 24 h and reduced CDK6 levels, we hypothesized that its effect on the cell cycle could also involve direct interaction with CDK6, in addition to the downregulation of its levels. Moreover, in our previous studies, a CA 1 derivative was shown to be a potential CDK6 inhibitor.^20^ Therefore, multiple molecular docking simulations were conducted to elucidate the binding mode of compound 17 and assess its interactions with CDK6. The CDK6 (PDB ID 6OQL) and compound 17 structures were prepared and validated using the methodology described by Moura *et al.*^20^ The selection of the optimal protocol, scoring function, and software is detailed elsewhere. The molecular docking results demonstrated that compound 17 adopts an optimal pose at the CDK6 binding site (Fig. 8A), interacting with amino acids ILE19, TYR24, VAL27, VAL77, HIS100, PHE98, VAL101, ASP104, ALA162, GLN149, and LEU152.

Further analysis of the docking simulations revealed that compound 17 adopts a pose highly similar to that of the ligand present in the X-ray crystal structure of CDK6 used for the docking studies (PDB ID 6OQL). The superposition of compound 17 and the crystallographic ligand is provided in the SI (Fig. S8). Compound 17 forms several interactions that are also present in the CDK6–ligand complex, specifically hydrophobic interactions with amino acid residues ILE19, VAL27, and LEU152, and hydrogen bonds with HIS100 and VAL101 (Fig. 8B and C). The distances between the atoms involved in these interactions are listed in the SI (Table S1). Additionally, compound 17 established a hydrogen bond with ILE19 and formed four extra hydrophobic interactions with the amino acids VAL77, PHE98, ASP104, and GLN149. These interactions contribute to stabilizing its position within the binding pocket, supporting the hypothesis that compound 17 could serve as a potential inhibitor of this enzyme.

#### Quantification of ROS levels in SW480 cells

ROS are byproducts generated during various cellular processes and serve as secondary messengers in cellular signaling pathways.^55–57^ When ROS levels surpass cellular antioxidant capacity, it results in damage to DNA, lipids, and proteins, thereby promoting tumorigenesis. In cancer, ROS play a dual role, acting either as pro-tumorigenic or anti-tumorigenic agents, depending on their concentration within the cells. A modest elevation in intracellular ROS levels favors pro-tumorigenic effects. Nevertheless, when ROS levels reach toxic concentrations, they assume an anti-tumorigenic role, inducing cell cycle arrest and cell death. Considering the dual role of ROS, approaches aimed at decreasing or increasing ROS levels in cancer cells have shown promise as therapeutic strategies.^55–59^ On the one hand, reducing ROS levels inhibits pro-tumorigenic signals, decreasing proliferation, cell survival, metabolic adaptations, and DNA damage. However, elevating ROS levels to toxic levels induces anti-tumorigenic signals by overwhelming the antioxidant system.

**Fig. 9:**
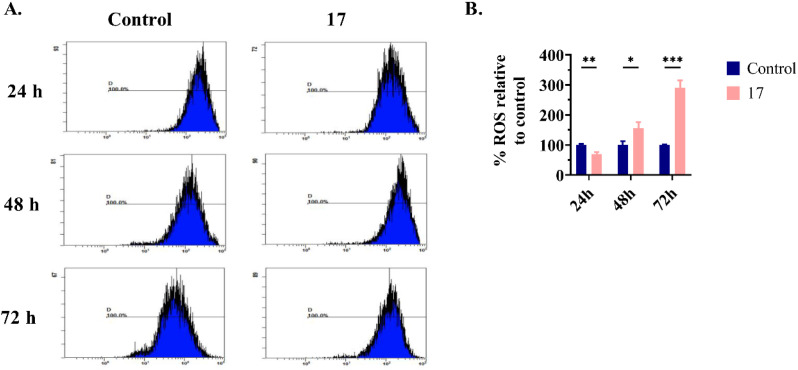
Determination of intracellular ROS levels in SW480 cells. Untreated cells (control) or cells treated with compound 17 at 6.3 μM (IC_50_ value) were incubated with H_2_DCFDA probe 24, 48 and 72 h pos-treatment. Before flow cytometry analysis, cells were stained with PI. In this analysis, the fluorescence emitted by the DCF probe was recorded only in cells that were negative for PI. (A) Each histogram represents DCF intensity, which is proportional to ROS levels. (B) Graphs show the percentage of ROS relative to the control cells. Data from three independent experiments are given as the mean ± SD. Multiple *t*-tests (one independent *t*-test per row) were used to assess differences between the control and treatment groups. Statistical significance was defined as *p* < 0.05 (*), *p* < 0.01 (**), or *p* < 0.001 (***).

Intracellular ROS levels can be quantified using lipophilic and non-fluorescent probes, such as 2′,7′-dichlorodihydrofluorescein diacetate (H_2_DCFDA).^60^ To assess the effect of compound 17 on intracellular ROS levels, SW480 cells were treated with compound 17, followed by incubation with the H_2_DCFDA probe after 24, 48, and 72 h of treatment. After, the cells were subjected to flow cytometry to quantify the fluorescence intensity of 2′,7′-dichlorofluorescein (DCF), as shown in Fig. 9. At 24 h, administration of compound 17 resulted in a reduction in intracellular ROS levels in SW480 cells relative to untreated cells (70 *vs.* 100%), as depicted in Fig. 9. However, at 48 and 72 h, compound 17 exhibited contrasting effects, causing an increase in intracellular ROS levels in SW480 cells to 157% and 290%, respectively. These results suggest that the effect of compound 17 on intracellular ROS levels is dependent on the time of exposure of the cells to the treatment, exhibiting an antioxidant role at 24 h and a pro-oxidant role at 48 and 72 h. Both effects of compound 17 contributed to the inhibition of SW480 cell growth.

The cellular redox environment regulates the cell cycle, and ROS levels determine whether the cell cycle is negatively or positively regulated.^61,62^ In our study, there seems to be a potential correlation between ROS levels and the cell cycle phase in which arrest occurs. At 24 h, a reduction in ROS levels could have led to cell cycle arrest at the G0/G1 phase in the SW480 cells. However, at 48 h, compound 17 arrested the cell cycle in another phase (S phase), which may be related to an increase in ROS levels. At 72 h, the concentration of ROS was further elevated; therefore, it was expected to arrest the cell cycle. Nevertheless, as observed in previous studies, no significant effect on the cell cycle was observed at 72 h. Further studies are required to investigate whether ROS levels influence the cell cycle of SW480 cells treated with compound 17.

#### Evaluation of SOD2/MnSOD levels in SW480 cells

Antioxidant systems play a crucial role in maintaining ROS homeostasis to prevent oxidative stress, involving both nonenzymatic and enzymatic antioxidant mechanisms.^56,58^ One family of enzymatic antioxidants important for scavenging ROS is superoxide dismutase (SOD).^56,58,63^ These metalloenzymes catalyze the conversion of superoxide anions to hydrogen peroxide and molecular oxygen, thereby regulating ROS levels. There are three types of SODs localized in different cellular compartments: SOD1 (Cu/ZnSOD) in the cytosol and mitochondrial intermembrane space, SOD2 (MnSOD) in the inner membrane and mitochondrial matrix, and SOD3 (Cu/ZnSOD) in the extracellular compartment. SOD2 (MnSOD) catalyzes the dismutation of the majority of superoxide anions produced within the mitochondria, which is the main organelle responsible for intracellular ROS generation.^56,58^

**Fig. 10:**
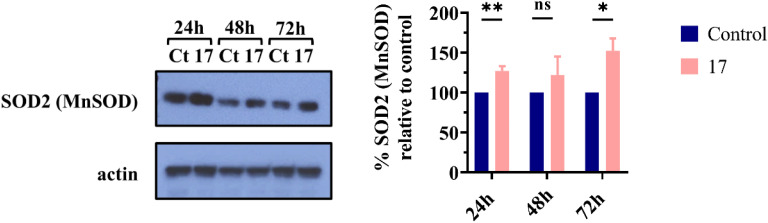
Analysis of SOD2 (MnSOD) levels by western blot. Untreated cells (control) and cells treated with compound 17 at 6.3 μM (IC_50_ value) were incubated for 24, 48, and 72 h. Actin served as the loading control. The graph illustrates the percentage of SOD2 (MnSOD) protein relative to the control group. Data are presented as mean ± SD from three independent experiments, except for 72 h (*n* = 2). Multiple *t*-tests (one independent *t*-test per row) were used to assess differences between the control and treatment groups. Statistical significance was defined as *p* < 0.05 (*) and *p* < 0.01 (**). ns, statistically non-significant.

Our studies revealed that compound 17 affects ROS levels; therefore, we assessed the effect of compound 17 on the expression of SOD2 (MnSOD) in SW480 cells after 24, 48, and 72 h of incubation. As shown in Fig. 10, compound 17 significantly increased the expression of SOD2/MnSOD at 24 and 72 h. At 24 h, overexpression of SOD2/MnSOD led to a decrease in ROS levels. However, after 72 h, the increased expression of SOD2 (MnSOD) was insufficient to reduce ROS levels.

## Experimental

### Chemistry

#### General

Solvents and reagents were obtained from VWR Portugal (Carnaxide, Portugal) and Sigma-Aldrich (St. Louis, MO, USA) and dried following standard procedures. CA 1 was purchased from Sigma-Aldrich (St. Louis, MO, USA). Thin-layer chromatography (TLC) plates of aluminum covered with silica gel 60 containing the fluorescent indicator, F_254_, were purchased from Merck Co. (Rahway, NJ, USA). Preparative TLCs were prepared by combination of 1 : 1 of silica gel 60 F_254_ and silica gel 60 purchased from Merck Co. (Rahway, NJ, USA). A Büchi® Mp B-540 apparatus (Büchi®, Flawil, Switzerland) was used to determine the melting points (mp), which were reported without adjustment. A PerkinElmer Spectrum 400 FT-IR/FT-NIR spectrometer (PerkinElmer, Waltham, MA, USA) was used to record the infrared (IR) spectra. Chemical structures were elucidated using 1D nuclear magnetic resonance (NMR) (^1^H, ^13^C, and DEPT-135) and 2D NMR (COSY, NOESY, HSQC, and HMBC). The samples were dissolved in CDCl_3_, and ^1^H and ^13^C spectra were recorded at 400 and 100 MHz, respectively, using a Bruker Avance III 400 MHz spectrometer (Bruker, Billerica, MA, USA). The coupling constants (*J*) are denoted in hertz (Hz), and the chemical shifts (*δ*) are represented in parts per million (ppm). The ^1^H and ^13^C spectra were calibrated at *δ* 7.26 ppm and *δ* 77.16 ppm, respectively. Mass spectrometry (MS) was conducted using a Thermo Scientific Finnigan LXQ Linear Ion Trap Mass Spectrometer (Thermo Fisher Scientific, Waltham, MA, USA) with electrospray ionization (ESI). The ESI conditions were set to 5 kV in the positive mode, with a sheath gas flow of 5 U and a capillary temperature of 250 °C. Elemental analysis was conducted using a TruSpec 630-200-200 CHNS analyzer (Leco Corporation, St. Joseph, MI, USA).

##### (4*aR*,10*aS*)-5,6-Bis(acetyloxy)-1,1-dimethyl-7-(propan-2-yl)1,3,4,9,10,10*a*-hexahydrophenanthrene-4*a*(2*H*)-carboxylic acid (2)

Compound 2 was prepared from CA 1 using the method previously reported in the literature.^20^ DMAP (22.5 mg, 0.18 mmol) and acetic anhydride (0.25 mL, 2.64 mmol) were added to a solution of CA 1 (150.0 mg, 0.45 mmol) in dry THF (3.50 mL) under anhydrous conditions, at room temperature. After 24 h, the THF was removed under reduced pressure. The crude obtained was extracted using ethyl acetate (3 × 30 mL) from water (15 mL). The organic layer was washed with HCl 5% (45 mL), NaHCO_3_ 10% (45 mL), H_2_O (2 × 45 mL), brine (45 mL), and dried over anhydrous Na_2_SO_4_. The obtained organic phase was filtered, and the solvent was removed under reduced pressure to obtain 2 (177.2 mg, 94%) as a white powder. Mp: 220.4–222.3 °C. IR (neat)_*v*_max__: 3022, 1777, 1768, 1689, 1194, 1186, 1166 cm^−1^. ^1^H NMR (400 MHz, CDCl_3_) *δ* 6.95 (1H, s, 14-H), 2.26 (3H, s, OCOCH̲_3_), 2.24 (3H, s, OCOCH̲_3_), 1.21 (3H, d, *J* = 6.9 Hz, CHCH̲_3_), 1.14 (3H, d, *J* = 6.9 Hz, CHCH̲_3_), 0.96 (3H, s, CH̲_3_), 0.86 (3H, s, CH̲_3_); ^13^C NMR (100 MHz, CDCl_3_) *δ* 179.61 (C20), 168.80 (OC̲OCH_3_), 168.33 (OC̲OCH_3_), 141.49, 140.07, 138.82, 136.93, 132.09, 125.36 (C14), 53.91, 47.70, 41.21, 34.66, 34.17, 32.62, 32.05, 27.52, 23.12, 22.87, 20.68, 20.56, 20.20, 19.96, 18.26. ESI-MS *m*/*z*: 439.3 [M + Na]^+^. Anal. calcd for C_24_H_32_O_6_·0.3C_6_H_14_: C, 70.05; H, 8.25. Found: C, 70.5; H, 7.9%.

##### Methyl(4*aR*,10*aS*)-5,6-bis(acetyloxy)-1,1-dimethyl-7-(propan-2-yl)-1,3,4,9,10,10*a*-hexahydrophenanthrene-4*a*(2*H*)-carboxylate (3)

Compound 3 was synthesized according to a previously described method, using another scaffold.^39^ Methyl iodide (0.06 mL, 0.96 mmol, 2 eq.) and anhydrous potassium carbonate (165.8 mg, 1.20 mmol, 2.5 eq.) was added to a stirred solution of 2 (200.0 mg, 0.48 mmol) in dry DMF (4 mL) under anhydrous conditions, at room temperature. After 2 h, the reaction mixture was extracted with ethyl acetate (3 × 50 mL) from water (50 mL). The organic layer was washed with HCl 5% (2 × 50 mL), NaHCO_3_ 10% (2 × 50 mL), H_2_O (2 × 50 mL) and brine (50 mL), dried over anhydrous Na_2_SO_4,_ filtered, and concentrated under reduced pressure to afford a crude product. The crude product was purified by preparative TLC (PE/EtOAc 5 : 1) to obtain the 3 as a white powder (190.0 mg, 92%). Mp: 158.2–160.0 °C. IR (neat)_*v*_max__: 3021, 1773, 1763, 1719, 1221, 1210, 1193, 1181 cm^−1^. ^1^H NMR (400 MHz, CDCl_3_) *δ* 6.95 (1H, s, 14-H), 3.51 (3H, s, COOCH̲_3_), 2.26 (3H, s, OCOCH̲_3_), 2.25 (3H, s, OCOCH̲_3_), 1.22 (3H, d, *J* = 6.9 Hz, CHCH̲_3_), 1.14 (3H, d, *J* = 6.9 Hz, CHCH̲_3_), 0.96 (3H, s, CH̲_3_), 0.74 (3H, s, CH̲_3_); ^13^C NMR (100 MHz, CDCl_3_) *δ* 175.31 (C20), 168.76 (OC̲OCH_3_), 168.43 (OC̲OCH_3_), 141.44, 139.82, 138.83, 136.90, 131.88, 125.24 (C14), 53.76, 51.90, 47.86, 41.27, 34.79, 34.08, 32.60, 32.01, 27.51, 23.16, 22.77, 20.84, 20.54, 20.04, 19.90, 18.46. ESI-MS *m*/*z*: 453.3 [M + Na]^+^, 882.7 [2M + Na]^+^. Anal. calcd for C_25_H_34_O_6_: C, 69.7; H, 8.0. Found: C, 69.9; H, 8.4%.

##### Ethyl(4*aR*,10*aS*)-5,6-bis(acetyloxy)-1,1-dimethyl-7-(propan-2-yl)-1,3,4,9,10,10*a*-hexahydrophenanthrene-4*a*(2*H*)-carboxylate (4)

Based on the method described for the compound 3, using compound 2 (200.0 mg, 0.48 mmol), ethyl iodide (0.08 mL, 0.96 mmol, 2 eq.), anhydrous potassium carbonate (165.8 mg, 1.20 mmol, 2.5 eq.) and dry DMF (4 mL) for 3 h to afford a crude product. The crude product was purified by preparative TLC (PE/EtOAc 5 : 1) to obtain the 4 as a white powder (122.0 mg, 57%). Mp: 125.2–127.1 °C. IR (neat)_*v*_max__: 3032, 1772, 1713, 1205, 1195, 1184, 1173 cm^−1^. ^1^H NMR (400 MHz, CDCl_3_) *δ* 6.94 (1H, s, 14-H), 4.06 (1H, dq, *J* = 10.9, 7.2 Hz, COOCH̲_2_CH_3_), 3.88 (1H, dq, *J* = 10.9, 7.1 Hz, COOCH̲_2_CH_3_), 2.25 (3H, s, OCOCH̲_3_), 2.24 (3H, s, OCOCH̲_3_), 1.22 (3H, d, *J* = 6.9 Hz, CHCH̲_3_), 1.16 (3H, t, *J* = 7.1 Hz, COOCH_2_CH̲_3_), 1.14 (3H, d, *J* = 6.9 Hz, CHCH̲_3_), 0.97 (3H, s, CH̲_3_), 0.78 (3H, s, CH̲_3_). ^13^C NMR (100 MHz, CDCl_3_) *δ* 174.72 (C20), 168.72 (OC̲OCH_3_), 168.25 (OC̲OCH_3_), 141.39, 139.69, 138.74, 136.92, 132.10, 125.09 (C14), 60.93 (OC̲H_2_), 53.70, 47.89, 41.33, 34.69, 34.19, 32.60, 31.95, 27.46, 23.19, 22.76, 20.88, 20.52, 20.15, 20.06, 18.43, 13.98 (OCH_2_C̲H_3_). ESI-MS *m*/*z*: 467.4 [M + Na]^+^, 910.6 [2M + Na]^+^. Anal. calcd for C_26_H_36_O_6_·0.1C_6_H_14_·0.4H_2_O: C, 69.4; H, 8.4. Found: C, 69.0; H, 8.8%.

##### Butyl(4*aR*,10*aS*)-5,6-bis(acetyloxy)-1,1-dimethyl-7-(propan-2-yl)-1,3,4,9,10,10*a*-hexahydrophenanthrene-4*a*(2*H*)-carboxylate (5)

Based on the method described for compound 3, using compound 2 (200.0 mg, 0.48 mmol), *N*-butyl bromide (0.10 mL, 0.96 mmol, 2 eq.), anhydrous potassium carbonate (165.8 mg, 1.20 mmol, 2.5 eq.) and dry DMF (4 mL) for 3 h to obtain a crude product. The crude product was purified by preparative TLC (PE/EtOAc 6 : 1) to afford the 5 as a white powder (191.3 mg, 84%). Mp: 86.8–88.1 °C. IR (neat)_*v*_max__: 3022, 1780, 1766, 1714, 1204, 1183 cm^−1^. ^1^H NMR (400 MHz, CDCl_3_) *δ* 6.93 (1H, s, 14-H), 3.94 (1H, dt, *J* = 10.9, 6.5 Hz, COOCH̲_2_CH_2_), 3.82 (1H, dt, *J* = 10.9, 6.8 Hz, COOCH̲_2_CH_2_), 2.25 (3H, s, OCOCH̲_3_), 2.24 (3H, s, OCOCH̲_3_), 1.20 (3H, d, *J* = 6.9 Hz, CHCH̲_3_), 1.14 (3H, d, *J* = 6.9 Hz, CHCH̲_3_), 0.97 (3H, s, CH̲_3_), 0.80 (3H, t, *J* = 7.4 Hz, COOCH_2_CH_2_CH_2_CH̲_3_), 0.77 (3H, s, CH̲_3_). ^13^C NMR (100 MHz, CDCl_3_) *δ* 174.88 (C20), 168.76 (OC̲OCH_3_), 168.22 (OC̲OCH_3_), 141.41, 139.76, 138.74, 136.94, 132.06, 125.02 (C14), 64.96 (OC̲H_2_), 53.57, 48.01, 41.33, 34.62, 34.19, 32.60, 31.92, 30.34, 27.46, 23.12, 22.92, 20.91, 20.54, 20.11, 20.06, 19.41, 18.54, 13.69 (OCH_2_CH_2_CH_2_CH̲_3_). ESI-MS *m*/*z*: 495.4 [M + Na]^+^, 966.6 [2M + Na]^+^. Anal. calcd for C_28_H_40_O_6_·0.2C_6_H_14_·0.1H_2_O: C, 71.3; H, 8.8. Found: C, 71.0; H, 9.2%.

##### Methyl(4*aR*,10*aS*)-5,6-bis(acetyloxy)-1,1-dimethyl-9-oxo-7-(propan-2-yl)-1,3,4,9,10,10*a*-hexahydrophenanthrene-4*a*(2*H*)-carboxylate (6)

Compound 6 was synthesized from compound 3 (100.0 mg, 0.23 mmol) according to the method described in the literature, using another scaffold.^39^ The crude was purified by preparative TLC (PE/EtOAc 4 : 1) to afford the 6 as a white powder (83.8 mg, 82%). Mp: 147.2–149.1 °C. IR (neat)_*v*_max__: 3026, 1778, 1727,1688, 1198, 1182, 1171, 1150, 1144 cm^−1^. ^1^H NMR (400 MHz, CDCl_3_) *δ* 8.06 (1H, s, 14-H), 3.55 (3H, s, COOCH̲_3_), 2.30 (3H, s, OCOCH̲_3_), 2.29 (3H, s, OCOCH̲_3_), 1.26 (3H, d, *J* = 6.9 Hz, CHCH̲_3_), 1.17 (3H, d, *J* = 6.9 Hz, CHCH̲_3_), 0.96 (3H, s, CH̲_3_), 0.80 (3H, s, CH̲_3_); ^13^C NMR (100 MHz, CDCl_3_) *δ* 197.65 (C7), 173.46 (C20), 168.14 (OC̲OCH_3_), 167.96 (OC̲OCH_3_), 145.58, 141.72, 141.14, 137.14, 131.42, 124.56 (C14), 52.32, 50.97, 48.70, 41.07, 35.43, 34.02, 33.96, 31.97, 27.83, 22.96, 22.63, 20.75, 20.52, 19.90, 19.44. ESI-MS *m*/*z*: 445.1 [M + H]^+^. Anal. calcd for C_25_H_32_O_7_: C, 67.55; H, 7.3. Found: C, 67.4; H, 7.75%.

##### Ethyl(4*aR*,10*aS*)-5,6-bis(acetyloxy)-1,1-dimethyl-9-oxo-7-(propan-2-yl)-1,3,4,9,10,10*a*-hexahydrophenanthrene-4*a*(2*H*)-carboxylate (7)

Compound 7 was synthesized from compound 4 (100.0 mg, 0.22 mmol) according to the method described in the literature, using another scaffold.^39^ The crude was purified by preparative TLC (PE/EtOAc 4 : 1) to obtain the 7 as a white powder (72.6 mg, 72%). Mp: 122.4–125.2 °C. IR (neat)_*v*_max__: 3039, 1771, 1718, 1691, 1192, 1171, 1159, 1140 cm^−1^. ^1^H NMR (400 MHz, CDCl_3_) *δ* 8.05 (1H, s, 14-H), 4.09 (1H, dq, *J* = 10.9, 7.2 Hz, COOCH̲_2_CH_3_), 3.93 (1H, dq, *J* = 10.9, 7.2 Hz, COOCH̲_2_CH_3_), 2.30 (3H, s, OCOCH̲_3_), 2.29 (3H, s, OCOCH̲_3_), 1.27 (3H, d, *J* = 6.9 Hz, CHCH̲_3_), 1.17 (3H, d, *J* = 6.9 Hz, CHCH̲_3_), 1.15 (3H, t, *J* = 7.2 Hz, COOCH_2_CH̲_3_), 0.96 (3H, s, CH̲_3_), 0.84 (3H, s, CH̲_3_). ^13^C NMR (100 MHz, CDCl_3_) *δ* 197.80 (C7), 172.85 (C20), 168.01 (OC̲OCH_3_), 167.96 (OC̲OCH_3_), 145.56, 141.62, 141.10, 137.33, 131.48, 124.50 (C14), 61.61 (OC̲H_2_), 50.95, 48.76, 41.14, 35.48, 34.09, 33.94, 31.96, 27.82, 22.99, 22.61, 20.81, 20.52, 19.94, 19.69, 13.95 (OCH_2_C̲H_3_). ESI-MS *m*/*z*: 459.1 [M + H]^+^. Anal. calcd for C_26_H_34_O_7_·0.5H_2_O: C, 66.8; H, 7.55. Found: C, 67.1; H, 8.1%.

##### Butyl(4*aR*,10*aS*)-5,6-bis(acetyloxy)-1,1-dimethyl-9-oxo-7-(propan-2-yl)-1,3,4,9,10,10*a*-hexahydrophenanthrene-4*a*(2*H*)-carboxylate (8)

Compound 8 was synthesized from compound 5 (100 mg, 0.21 mmol) according to the method described in the literature, using another scaffold.^39^ The crude was purified by preparative TLC (PE/EtOAc 6 : 1) to obtain the 8 as a white powder (77.7 mg, 76%). Mp: 88.2–90.1 °C. IR (neat)_*v*_max__: 3012, 1781, 1761, 1714, 1691, 1193, 1173, 1155, 1138 cm^−1^. ^1^H NMR (400 MHz, CDCl_3_) *δ* 8.05 (1H, s, 14-H), 4.00–3.93 (1H, m, COOCH̲_2_CH_2_), 3.89 (1H, dt, *J* = 10.8, 6.8 Hz, COOCH̲_2_CH_2_), 2.29 (3H, s, OCOCH̲_3_), 2.30 (3H, s, OCOCH̲_3_), 1.25 (3H, d, *J* = 6.9 Hz, CHCH̲_3_), 1.18 (3H, d, *J* = 6.9 Hz, CHCH̲_3_), 0.96 (3H, s, CH̲_3_), 0.83 (3H, s, CH̲_3_), 0.79 (3H, t, *J* = 7.4 Hz, COOCH_2_CH_2_CH_2_CH̲_3_).^13^C NMR (100 MHz, CDCl_3_) *δ* 197.77 (C7), 172.98 (C20), 167.98 (OC̲OCH_3_), 167.94 (OC̲OCH_3_), 145.54, 141.66, 141.11, 137.33, 131.51, 124.46 (C14), 65.58 (OC̲H_2_), 50.88, 48.84, 41.12, 35.53, 34.08, 33.89, 31.94, 30.25, 27.80, 22.92, 22.69, 20.80, 20.51, 19.93, 19.61, 19.34, 13.60 (OCH_2_CH_2_CH_2_CH̲_3_). ESI-MS *m*/*z*: 487.2 [M + H]^+^. Anal. calcd for C_28_H_38_O_7_: C, 69.1; H, 7.9. Found: C, 69.0; H, 8.3%.

##### Methyl(4*aR*,10*aS*)-5,6-bis(acetyloxy)-9-hydroxy-1,1-dimethyl-7-(propan-2-yl)-1,3,4,9,10,10*a*-hexahydrophenanthrene-4*a*(2*H*)-carboxylate (9)

Compound 9 was synthesized from compound 6 (80.0 mg, 0.18 mmol) according to the method described in the literature, using another scaffold.^64^ The crude was purified by preparative TLC (PE/EtOAc 3 : 1) to afford the 9 as a light-yellow powder (60.5 mg, 76%). Mp: 89.1–91.8 °C. IR (neat)_*v*_max__: 3503, 3025, 1771, 1718, 1202, 1176 cm^−1^. ^1^H NMR (400 MHz, CDCl_3_) *δ* 7.58 (1H, s, 14-H), 4.78–4.69 (1H, m, 7-H), 3.54 (3H, s, COOCH̲_3_), 2.26 (3H, s, OCOCH̲_3_), 2.24 (3H, s, OCOCH̲_3_), 1.24 (3H, d, *J* = 6.9 Hz, CHCH̲_3_), 1.17 (3H, d, *J* = 6.9 Hz, CHCH̲_3_), 0.97 (3H, s, CH̲_3_), 0.75 (3H, s, CH̲_3_); ^13^C NMR (100 MHz, CDCl_3_) *δ* 174.86 (C20), 168.56 (OC̲OCH_3_), 168.34 (OC̲OCH_3_), 140.84, 140.66, 140.12, 140.00, 131.99, 123.58 (C14), 71.03 (C7), 52.11, 51.18, 48.54, 41.00, 34.63, 33.77, 32.45, 29.25, 27.79, 23.04, 22.92, 20.77, 20.53, 19.88 (2C). ESI-MS *m*/*z*: 469.3 [M + Na]^+^. Anal. calcd for C_25_H_34_O_7_·1H_2_O: C, 64.6; H, 7.8. Found: C, 64.3; H, 8.1%.

##### Ethyl(4*aR*,10*aS*)-5,6-bis(acetyloxy)-9-hydroxy-1,1-dimethyl-7-(propan-2-yl)-1,3,4,9,10,10*a*-hexahydrophenanthrene-4*a*(2*H*)-carboxylate (10)

Compound 10 was synthesized from compound 7 (80.0 mg, 0.18 mmol) according to the method described in the literature, using another scaffold.^64^ The crude was purified by preparative TLC (PE/EtOAc 3 : 1) to obtain the 10 as a light-yellow powder (67.1 mg, 81%). Mp: 83.0–85.6 °C. IR (neat)_*v*_max__: 3510, 3025, 1772, 1715, 1203, 1184, 1174 cm^−1^. ^1^H NMR (400 MHz, CDCl_3_) *δ* 7.56 (1H, s, 14-H), 4.79–4.69 (1H, m, 7-H), 4.09 (1H, dq, *J* = 10.9, 7.2 Hz, COOCH̲_2_CH_3_), 3.91 (1H, dq, *J* = 10.9, 7.1 Hz, COOCH̲_2_CH_3_), 2.26 (3H, s, OCOCH̲_3_), 2.24 (3H, s, OCOCH̲_3_), 1.60 (1H, dd, *J* = 13.0, 1.9 Hz, 5-H), 1.24 (3H, d, *J* = 6.9 Hz, CHCH̲_3_), 1.19 (3H, t, *J* = 7.2 Hz, COOCH_2_CH̲_3_), 1.17 (3H, d, *J* = 6.9 Hz, CHCH̲_3_), 0.98 (3H, s, CH̲_3_), 0.79 (3H, s, CH̲_3_). ^13^C NMR (100 MHz, CDCl_3_) *δ* 174.28 (C20), 168.54 (OC̲OCH_3_), 168.20 (OC̲OCH_3_), 140.76, 140.64, 140.10, 139.98, 132.31, 123.60 (C14), 71.01 (C7), 61.26 (OC̲H_2_), 51.23 (C5), 48.57, 41.08, 34.56, 33.89, 32.46, 29.27, 27.78, 23.09, 22.90, 20.84, 20.53, 20.14, 19.94, 14.04 (OCH_2_C̲H_3_). ESI-MS *m*/*z*: 483.3 [M + Na]^+^. Anal. calcd for C_26_H_36_O_7_·0.2C_6_H_14_·0.5H_2_O: C, 67.1; H, 8.2. Found: C, 66.8; H, 8.75%.

##### Butyl(4*aR*,10*aS*)-5,6-bis(acetyloxy)-9-hydroxy-1,1-dimethyl-7-(propan-2-yl)-1,3,4,9,10,10*a*-hexahydrophenanthrene-4*a*(2*H*)-carboxylate (11)

Compound 11 was synthesized from compound 8 (80.0 mg, 0.16 mmol) according to the method described in the literature, using another scaffold.^64^ The crude was purified by preparative TLC (PE/EtOAc 5 : 1) to obtain the 11 as a dark yellow powder (61.0 mg, 78%). Mp: 63.1–65.8 °C. IR (neat)_*v*_max__: 3516, 3026, 1773, 1715, 1202, 1183, 1172 cm^−1^. ^1^H NMR (400 MHz, CDCl_3_) *δ* 7.56 (1H, s, 14-H), 4.74 (1H, dd, *J* = 10.2, 7.0 Hz, 7-H), 3.98 (1H, dt, *J* = 10.9, 6.6 Hz, COOCH̲_2_CH_2_), 3.85 (1H, dt, *J* = 10.8, 6.9 Hz, COOCH̲_2_CH_2_), 2.26 (3H, s, OCOCH̲_3_), 2.24 (3H, s, OCOCH̲_3_), 1.23 (3H, d, *J* = 6.8 Hz, CHCH̲_3_), 1.17 (3H, d, *J* = 6.9 Hz, CHCH̲_3_), 0.98 (3H, s, CH̲_3_), 0.83 (3H, t, *J* = 7.4 Hz, COOCH_2_CH_2_CH_2_CH̲_3_), 0.78 (3H, s, CH̲_3_). ^13^C NMR (100 MHz, CDCl_3_) *δ* 174.40 (C20), 168.54 (OC̲OCH_3_), 168.16 (OC̲OCH_3_), 140.78, 140.63, 140.06, 139.97, 132.26, 123.47 (C14), 70.97 (C7), 65.28 (OC̲H_2_), 51.13, 48.64, 41.05, 34.49, 33.87, 32.44, 30.35, 29.32, 27.76, 23.01, 22.98, 20.84, 20.52, 20.06, 19.92, 19.46, 13.74 (OCH_2_CH_2_CH_2_CH̲_3_). ESI-MS *m*/*z*: 511.3 [M + Na]^+^. Anal. calcd for C_28_H_40_O_7_: C, 68.8; H, 8.25. Found: C, 68.8; H, 8.7%.

##### (4*bR*,8*aS*)-4*b*-Isocyanato-8,8-dimethyl-2-(propan-2-yl)-4*b*,5,6,7,8,8*a*,9,10-octahydrophenanthrene-3,4-diyl diacetate (14)

Compound 14 was prepared from compound 2 according to the method described in the literature.^20^ Oxalyl chloride (0.16 mL, 1.90 mmol) and dry DMF (0.15 mL, 0.72 mmol) were added to a stirred solution of 2 (300.0 mg, 0.72 mmol) in dry CH_2_Cl_2_ (8 mL) in reflux at 40 °C under N_2_ atmosphere. After 5 h 30 min, the solution was concentrated under vacuum to afford a crude 12. At 0 °C, to a stirred aqueous solution of sodium azide (176.5 mg, 2.71 mmol in 1.80 mL of H_2_O) was added dropwise the crude 12 dissolved in acetone (8 mL) and Et_3_N (1.50 mmol, 0.21 mL). After 22 h at room temperature, the mixture was extracted from water (7.5 mL) using ethyl acetate (3 × 15 mL). The organic layer was washed with brine (22.5 mL), dried over anhydrous Na_2_SO_4_, filtered and the solvent removed under reduced pressure to afford 13. The crude 13 was dissolved in toluene (8 mL) and the reaction was maintained at 115 °C (in reflux) under an N_2_ atmosphere. After 1 h, toluene was evaporated under vacuum to obtain 14 (232.2 mg, 78%) as a dark yellow powder. Mp: 58.4–61.3 °C. IR (neat)_*v*_max__: 3018, 2247, 1777, 1197, 1181, 1170 cm^−1^. ^1^H NMR (400 MHz, CDCl3) *δ* 6.94 (1H, s, 14-H), 2.32 (3H, s, OCOCH̲_3_), 2.29 (3H, s, OCOCH̲_3_), 1.22 (3H, d, *J* = 6.9 Hz, CHCH̲_3_), 1.16 (3H, d, *J* = 6.9 Hz, CHCH̲_3_), 1.03 (3H, s, CH̲_3_), 0.98 (3H, s, CH̲_3_); ^13^C NMR (100 MHz, CDCl_3_) *δ* 168.67 (OC̲OCH_3_), 168.10 (OC̲OCH_3_), 141.54, 141.20, 139.19, 135.78, 132.22, 125.17 (C14), 123.40 (NC̲O), 61.07, 53.29, 40.66, 37.32, 33.89, 32.85, 32.31, 27.61, 22.99, 22.93, 21.16, 21.02, 20.56, 19.43, 19.28. ESI-MS *m*/*z*: 436.3 [M + Na]^+^. Anal. calcd for C_24_H_31_NO_5_·0.2C_6_H_14_: C, 70.3; H, 7.9; N, 3.25. Found: C, 70.5; H, 7.6; N, 3.2%.

##### (4*bR*,8*aS*)-4*b*-[(Methoxycarbonyl)amino]-8,8-dimethyl-2-(propan-2-yl)-4*b*,5,6,7,8,8*a*,9,10-octahydrophenanthrene-3,4-diyl diacetate (15)

Dry methanol (2 mL) and Et_3_N (0.36 mL) were added to a stirred solution of 14 (110.0 mg, 0.27 mmol) in dry THF (4.5 mL) at 40 °C under N_2_ atmosphere. After 25 h 40 min, more dry methanol (0.5 mL) was added to the reaction mixture. After 4 h, the reaction mixture was concentrated under reduced pressure and purified by preparative TLC (PE/EtOAc 3 : 1) to obtain the 15 as a light-yellow powder (60.2 mg, 50%). Mp: 164.7–166.4 °C. IR (neat)_*v*_max__: 3437, 3060, 1770, 1735, 1515, 1228, 1197, 1176, 1146, 1108 cm^−1^. ^1^H NMR (400 MHz, CDCl_3_) *δ* 6.91 (1H, s, 14-H), 4.76 (1H, br s, NH̲), 3.50 (3H, s, OCH̲_3_), 2.28 (3H, s, OCOCH̲_3_), 2.27 (3H, s, OCOCH̲_3_), 1.20 (3H, d, *J* = 6.9 Hz, CHCH̲_3_), 1.14 (3H, d, *J* = 6.9 Hz, CHCH̲_3_), 0.97 (3H, s, CH̲_3_), 0.96 (3H, s, CH̲_3_); ^13^C NMR (100 MHz, CDCl_3_) *δ* 168.80 (OC̲OCH_3_), 168.73 (OC̲OCH_3_), 154.48 (NHC̲O), 141.37, 140.17, 138.88, 136.20, 133.35, 124.73 (C14), 56.34, 53.21, 51.76, 40.79, 34.40, 33.92, 32.96, 31.59, 27.55, 23.13, 22.81, 21.48, 21.40, 20.57, 19.40, 18.12. ESI-MS *m*/*z*: 468.3 [M + Na]^+^, 912.5 [2M + Na]^+^. Anal. calcd for C_25_H_35_NO_6_·0.1C_6_H_15_N·0.3H_2_O: C, 66.7; H, 8.1; N, 3.3. Found: C, 66.4; H, 8.5; N, 3.1%.

##### (4*bR*,8*aS*)-4*b*-[(Ethoxycarbonyl)amino]-8,8-dimethyl-2-(propan-2-yl)-4*b*,5,6,7,8,8*a*,9,10-octahydrophenanthrene-3,4-diyl diacetate (16)

Dry ethanol (7.61 mL) and Et_3_N (0.83 mL) were added to a stirred solution of 14 (168.0 mg, 0.41 mmol) in dry THF (6.83 mL) at 40 °C under N_2_ atmosphere. After 23 h, the temperature was increased to 50 °C. After 24 h, dry ethanol (1.9 mL) was added to the reaction mixture. After a total of 71 h, the reaction mixture was concentrated under reduced pressure and purified by preparative TLC (PE/EtOAc 3 : 1) to afford 16 as a beige-salmon powder (60.6 mg, 32%). Mp: 77.5–79.9 °C. IR (neat)_*v*_max__: 3447, 3018, 1775, 1729, 1687, 1514, 1228, 1200, 1174, 1150, 1106 cm^−1^. ^1^H NMR (400 MHz, CDCl_3_) *δ* 6.91 (1H, s, 14-H), 4.72 (1H, br s, NH̲), 3.99–3.87 (2H, m, OCH̲_2_CH_3_), 2.28 (3H, s, OCOCH̲_3_), 2.27 (3H, s, OCOCH̲_3_), 1.20 (3H, d, *J* = 6.8 Hz, CHCH̲_3_), 1.18–1.11 (3H, m, OCH_2_CH̲_3_), 1.14 (3H, d, *J* = 6.9 Hz, CHCH̲_3_), 0.98 (3H, s, CH̲_3_), 0.96 (3H, s, CH̲_3_); ^13^C NMR (100 MHz, CDCl_3_) *δ* 168.76 (OC̲OCH_3_), 168.56 (OC̲OCH_3_), 154.15 (NHC̲O), 141.44, 140.12, 138.91, 135.80, 133.47, 124.68 (C14), 60.31 (OC̲H_2_), 55.73, 53.33, 40.79, 34.49, 33.89, 32.98, 31.66, 27.56, 23.10, 22.86, 21.49, 21.35, 20.55, 19.42, 18.14, 14.74 (OCH_2_C̲H_3_). ESI-MS *m*/*z*: 482.4 [M + Na]^+^, 940.5 [2M + Na]^+^. Anal. calcd for C_26_H_37_NO_6_·0.15C_6_H_15_N·0.4H_2_O: C, 67.0; H, 8.4; N, 3.3. Found: C, 67.2; H, 8.9; N, 2.9%.

##### (4*bR*,8*aS*)-8,8-Dimethyl-4*b*-{[(2-methylpropoxy)carbonyl]amino}-2-(propan-2-yl)-4*b*,5,6,7,8,8*a*,9,10-octahydrophenanthrene-3,4-diyl diacetate (17)

Dry 2-methylpropan-1-ol (6.68 mL) and Et_3_N (0.73 mL) were added to a stirred solution of 14 (150.0 mg, 0.36 mmol) in dry THF (6 mL) at 40 °C under N_2_ atmosphere. After 42 h, the temperature was increased to 50 °C. After 23 h, dry 2-methylpropan-1-ol (1.60 mL) was added to the reaction mixture. After a total of 72 h, the reaction mixture was concentrated under reduced pressure and purified by preparative TLC (PE/EtOAc 5 : 1) to obtain 17 as a beige-yellow powder (100.5 mg, 57%). Mp: 72.6–74.1 °C. IR (neat)_*v*_max__: 3447, 3025, 1776, 1731, 1685, 1513, 1226, 1201, 1174, 1149 cm^−1^. ^1^H NMR (400 MHz, CDCl_3_) *δ* 6.91 (1H, s, 14-H), 4.74 (1H, br s, NH̲), 3.75–3.66 (1H, m, OCH̲_2_), 3.66–3.53 (2H, m, OCH̲_2_ (1H)), 2.26 (6H, s, 2 × OCOCH̲_3_), 1.20 (3H, d, *J* = 7.0 Hz, CHCH̲_3_), 1.14 (3H, d, *J* = 6.9 Hz, CHCH̲_3_), 0.98 (3H, s, CH̲_3_), 0.96 (3H, s, CH̲_3_), 0.85 (6H, d, *J* = 4.2 Hz, OCH_2_CH(CH̲_3_)_2_); ^13^C NMR (100 MHz, CDCl_3_) *δ* 168.81 (OC̲OCH_3_), 168.53 (OC̲OCH_3_), 154.24 (NHC̲O), 141.40, 140.06, 138.87, 136.34, 133.30, 124.67 (C14), 70.61 (OC̲H_2_), 56.12, 53.36, 40.73, 34.63, 33.87, 32.99, 31.73, 27.97, 27.53, 23.14, 22.79, 21.42, 21.32, 20.56, 19.39, 19.17 (2C, OCH_2_CH (C̲H_3_)_2_), 18.16. ESI-MS *m*/*z*: 510.4 [M + Na]^+^, 996.6 [2M + Na]^+^. Anal. calcd for C_28_H_41_NO_6_·0.2C_6_H_15_N·0.2H_2_O: C, 68.6; H, 8.75; N, 3.3. Found: C, 68.3; H, 9.3; N, 2.8%.

##### (4*bR*,8*aS*)-4*b*-[(Methoxycarbonyl)amino]-8,8-dimethyl-10-oxo-2-(propan-2-yl)-4*b*,5,6,7,8,8*a*,9,10-octahydrophenanthrene-3,4-diyl diacetate (18)

Compound 18 was synthesized from compound 15 (180.0 mg, 0.40 mmol) according to the method described in the literature, using another scaffold.^39^ The crude was purified by preparative TLC (PE/EtOAc 1 : 1) to obtain 18 as a white powder (119.2 mg, 65%). Mp: 67.3–69.7 °C. IR (neat)_*v*_max__: 3435, 3067, 1773, 1744, 1690, 1508, 1188, 1169, 1142 cm^−1^. ^1^H NMR (400 MHz, CDCl_3_) *δ* 8.00 (1H, s, 14-H), 4.89 (1H, br s, NH̲), 3.49 (3H, s, OCH̲_3_), 2.30 (3H, s, OCOCH̲_3_), 2.30 (3H, s, OCOCH̲_3_), 1.27 (3H, d, *J* = 6.9 Hz, CHCH̲_3_), 1.16 (3H, d, *J* = 6.9 Hz, CHCH̲_3_), 1.05 (3H, s, CH̲_3_), 0.96 (3H, s, CH̲_3_); ^13^C NMR (100 MHz, CDCl_3_) *δ* 197.39 (C7), 167.93 (2 × OC̲OCH_3_), 155.22 (NHC̲O), 145.49, 141.78, 140.93, 136.83, 132.00, 123.76 (C14), 56.41, 52.21, 50.10, 40.44, 35.61, 34.81, 33.63, 32.47, 27.92, 23.06, 22.53, 21.04, 21.01, 20.53, 18.82. ESI-MS *m*/*z*: 482.3 [M + Na]^+^. Anal. calcd for C_25_H_33_NO_7_·0.7H_2_O: C, 63.6; H, 7.3; N, 3.0. Found: C, 63.75; H, 7.7; N, 2.8%.

##### (4*bR*,8*aS*)-4*b*-[(Ethoxycarbonyl)amino]-8,8-dimethyl-10-oxo-2-(propan-2-yl)-4*b*,5,6,7,8,8*a*,9,10-octahydrophenanthrene-3,4-diyl diacetate (19)

Compound 19 was synthesized from compound 16 (160.0 mg, 0.35 mmol) according to the method described in the literature, using another scaffold.^39^ The crude was purified by preparative TLC (PE/EtOAc 2 : 1) to afford the 19 as a white powder (123.0 mg, 74%). Mp: 67.5–69.2 °C. IR (neat)_*v*_max__: 3373, 3026, 1780, 1727, 1684, 1521, 1193, 1170, 1143 cm^−1^. ^1^H NMR (400 MHz, CDCl_3_) *δ* 8.00 (1H, s, 14-H), 4.86 (1H, br s, NH̲), 4.00–3.85 (2H, m, OCH̲_2_CH_3_), 2.30 (3H, s, OCOCH̲_3_), 2.29 (3H, s, OCOCH̲_3_), 1.26 (3H, d, *J* = 6.9 Hz, CHCH̲_3_), 1.17 (3H, d, *J* = 7.1 Hz, CHCH̲_3_), 1.15–1.07 (3H, m, OCH_2_CH̲_3_), 1.05 (3H, s, CH̲_3_), 0.96 (3H, s, CH̲_3_); ^13^C NMR (100 MHz, CDCl_3_) *δ* 197.55 (C7), 167.98 (OC̲OCH_3_), 167.85 (OC̲OCH_3_), 154.84 (NHC̲O), 145.45, 141.70, 140.89, 136.91, 132.07, 123.68 (C14), 60.96 (OC̲H_2_), 56.27, 50.14, 40.39, 35.69, 34.86, 33.57, 32.50, 27.86, 23.04, 22.58, 21.00 (2C), 20.53, 18.80, 14.52. ESI-MS *m*/*z*: 496.3 [M + Na]^+^. Anal. calcd for C_26_H_35_NO_7_·0.6H_2_O: C, 64.5; H, 7.5; N, 2.9. Found: C, 64.45; H, 8.1; N, 2.8%.

### Biology

#### Materials and cells

A375, BJ, Caco-2, HCT116, Mia Paca-2, SW480, and SW620 cells were obtained from the American Type Culture Collection (ATCC, Manassas, VA, USA). Dulbecco's Modified Eagle Medium (DMEM) without sodium pyruvate, l-glutamine, and HEPES and with phenol red and 25 mM d-glucose (# 11960044); DMEM without sodium pyruvate, d-glucose, and HEPES and with phenol red, and 4 mM l-glutamine (# 11966025); l-glutamine (200 mM); trypsin–EDTA (0.05%) with phenol red; Fetal Bovine Serum (FBS) (# 10270-106); Hanks' Balanced Salt Solution (HBSS) with 5.56 mM d-glucose, magnesium, and calcium and without sodium pyruvate, and phenol red; penicillin (10.000 U mL^−1^)/streptomycin (10.000 U mL^−1^) (P/S) solution; sodium pyruvate (100 mM); trypan blue solution (0.4%); Dimethyl Sulfoxide (DMSO) (≥99.7%); PI; annexin V coupled with fluorescein isothiocyanate (FITC); Pierce™ bovine serum albumin standard ampules (2 mg mL^−1^); and Pierce™ BCA protein assay reagents A and B were purchased from Thermo Fisher Scientific (Waltham, MA, USA). HyClone Dulbecco's Phosphate Buffered Saline (DPBS) without magnesium, and calcium was purchased from Cytiva (Marlborough, MA, USA). Ham's F12 medium with 1 mM l-glutamine, 10 mM glucose, and phenol red (# L0135) was purchased from BioWest (Nuaillé, France). DMSO (≥99.5%), thiazolyl blue tetrazolium bromide (MTT), NaH_2_PO_4_·2H_2_O, NaCl, Na_2_HPO_4_·12H_2_O, Tris and acrylamide were purchased from PanReac AppliChem (Barcelona, USA). H_2_DCFDA, d-(+)-glucose solution (405–495 g L^−1^), dithiothreitol (DTT), Triton X-100, HEPES, NaOH, HCl, CaCl_2_, ethanol, Tween-20, Na-EDTA, sodium dodecyl sulfate (SDS) and sodium deoxycholate were purchased from Sigma-Aldrich (St. Louis, MO, USA). Immobilon®-P Polyvinylidene Fluoride (PVDF) membranes, phosphatase inhibitor cocktail, protease inhibitor cocktail, and Immobilon Western Chemiluminescent HRP Substrate Kit were purchased from Merck Millipore (Darmstadt, Germany). Skimmed milk was purchased from Nestlé (Vevey, Switzerland). Bovine pancreas-derived RNase powder was purchased from Roche Diagnostics (Rotkreuz, Switzerland). Blue-sensitive X-ray films (CP-BU) were purchased from AGFA (Mortsel, Belgium). Monoclonal mouse primary antibody targeting actin (# 691001) was purchased from MP Biomedicals (Santa Ana, CA, USA). Rabbit polyclonal IgG primary antibodies targeting Cdk4 (sc-260), Cdk6 (sc-177), cyclin A (sc-751), and Rb (sc-50) were purchased from Santa Cruz Biotechnology (Dallas, TX, USA). Rabbit monoclonal primary antibody targeting SOD2/MnSOD (ab68155), as well as the secondary antibodies conjugated with HRP (anti-mouse and anti-rabbit) were purchased from Abcam (Cambridge, UK).

#### Compounds

CA 1 and its derivatives were kept in powdered form at −20 °C. Prior to each experiment, the compounds were weighed, dissolved in DMSO, and diluted in the growth medium to obtain working solutions at the desired concentration. In all the working solutions, the concentration of DMSO was consistently less than or equal to 0.5%.

#### Cell culture

A375 and Mia Paca-2 cells were grown in DMEM (without d-glucose) supplemented with 1% P/S solution, 10% FBS, and 25 mM d-glucose. BJ cells were maintained in DMEM (high glucose) supplemented with 1 mM sodium pyruvate, 2 mM l-glutamine, 1% P/S solution, and 10% FBS. Caco-2 cells were cultured in DMEM (without d-glucose) supplemented with 1% P/S solution, 10% FBS, and 10 mM d-glucose. HCT116 cells were grown in a mixture (1 : 1) of DMEM (without d-glucose) and Ham's F12 supplemented with 0.5% P/S solution, 10% FBS, and 7.5 mM d-glucose. SW480 and SW620 cells were maintained in DMEM (without d-glucose) supplemented with 1% P/S solution, 5% FBS, and 12.5 mM d-glucose. Cells were maintained in a humidified atmosphere containing 5% CO_2_ at 37 °C. All studies used subconfluent cell monolayers.

#### Cell viability assay

The MTT assay was used to assess the effect of the compounds on cell viability. For this purpose, A375 (1500 cells per well), BJ (10 000 cells per well), Caco-2 (4000 cells per well), HCT116 (3000 cells per well), Mia Paca-2 (2000 cells per well), SW480 (6000 cells per well), and SW620 (8000 cells per well) cells were seeded in 96-well plates and incubated for 24 h. Subsequently, the culture medium was switched with a new medium containing DMSO (control) or the compound at a specified concentration. Following a 72 h incubation period, the supernatant was aspirated, and 100 μL of a mixture (1 : 1) of filtered MTT solution (1 mg per mL PBS) and FBS-free medium was added. The plates were then incubated for 1 h at 37 °C. After this time, the supernatant was aspirated, and the formazan crystals were dissolved in 100 μL of DMSO in each well. Relative cell viability was calculated by measuring absorbance at 570 nm using a Benchmark Plus Microplate Reader (Bio-Rad Laboratories, Hercules, CA, USA). Results are presented as the mean ± standard deviation (SD) of IC_50_ values, determined by nonlinear regression analysis of dose–response curves (log(inhibitor) *vs.* response with variable slope (four parameters)) using GraphPad Prism 9 software (GraphPad Software, San Diego, CA, USA).

#### Cell proliferation assay and cell doubling time

SW480 cells (1.68 × 10^5^ cells per well) were seeded in 6-well plates and incubated at 37 °C for 24 h. The cells were then treated with compound 17 (IC_50_ value) for 24, 48 or 72 h. At 0 h time (without treatment), the cells were harvested by trypsinization, washed with DPBS, collected by centrifugation, and resuspended in DPBS containing trypan blue. Cell viability was determined using the Invitrogen Countess 3 Automated Cell Counter (Thermo Fisher Scientific, Waltham, MA, USA). At 24, 48 and 72 h, the same process was repeated for the control and treated cells, with the aim of depicting a cell proliferation curve over time. The collected data also permitted the determination of cell doubling time by splitting the natural logarithm of 2 by the growth rate.^30^

#### Apoptosis assay

SW480 cells were seeded at a density of 1.68 × 10^5^ cells per well in 6-well plates and incubated at 37 °C. After 24 h of incubation, cells were treated with compound 17 (IC_50_ value) for 24, 48 or 72 h. Adherent cells were harvested by trypsinization, and they were washed with DPBS and collected by centrifugation along with the cells in suspension. Cells were resuspended in 3 μL annexin V FITC and 95 μL binding buffer containing 140 mM NaCl, 2.5 mM CaCl_2_, and 10 mM HEPES/NaOH (pH 7.4). The cell suspension was then incubated in the dark, at room temperature for 30 min. The cells were then stained with 510 μL of a mixture containing 500 μL of binding buffer and 10 μL of PI solution (1 mg mL^−1^). Finally, data from 1 × 10^4^ cells were collected through fluorescence activated cell sorting (FACS) analysis using a Gallios flow cytometer (Beckman Coulter, Brea, CA, USA) at 488 nm.

#### Cell cycle assay

SW480 cells were seeded, treated, incubated, and collected, as described for the apoptosis assay. After centrifugation, the cells were resuspended in 500 μL DPBS, added dropwise to 4.5 mL of 70% (v/v) cold ethanol, and stored at −20 °C for at least 4 h. Each cell suspension was centrifuged, washed with cold DPBS, and resuspended in 350 μL DPBS and 10 μL RNase (10 mg mL^−1^). After 1 h of incubation at 37 °C with agitation, the cells were stained with 20 μL of PI solution (1 mg mL^−1^). A Gallios flow cytometer (Beckman Coulter, Brea, CA, USA) at a wavelength of 488 nm was used for FACS analysis. Data from 1 × 10^4^ cells were collected, and a multicycle software (Phoenix Flow Systems, San Diego, CA, USA) was used for cell cycle analysis.

#### Intracellular ROS quantification assay

SW480 cells were seeded at a density of 1.68 × 10^5^ cells per well in 6-well plates and incubated at 37 °C for 24 h. The cells were then treated with compound 17 (IC_50_ value) for 24, 48, or 72 h. The adherent cells were trypsinized, washed with DPBS, collected by centrifugation, and resuspended in 1 mL of HBSS containing 50 μM H_2_DCFDA probe and 2 mM l-glutamine. After 45 min of incubation at 37 °C in a cell culture incubator, the cell suspensions were washed with 2 mL DPBS and collected by centrifugation. The cells were then stained with 350 μL of DPBS containing 20 μg per mL PI solution (1 mg mL^−1^). Inside the cells, the H_2_DCFDA probe is deacetylated and undergoes oxidation in the presence of ROS, transforming into a DCF fluorescent compound. The fluorescence emitted by DCF from 1 × 10^4^ PI-negative cells was measured at 520 nm using a Gallios flow cytometer (Beckman Coulter, Brea, CA, USA).

#### Preparation of total protein extracts and western blot analysis

SW480 cells were seeded in 6-well plates and treated as described in the previous subsection. After 24, 48 or 72 h of incubation, adherent cells were washed twice with ice-cold DPBS and incubated with lysis buffer (0.2% Triton X-100, 0.5 mM sodium deoxycholate, 1 mM Na-EDTA, 1 mM DTT, 20 mM Tris–HCl (pH 7.5), 0.5% phosphatases inhibitor cocktail, and 1% proteases inhibitor cocktail) for 30 min at 4 °C. The cells were scraped, sonicated, and centrifuged at 120 00 × *g* (20 min at 4 °C). After, the protein concentration on supernatants was quantified using Pierce™ bovine serum albumin standard ampules (2 mg mL^−1^) and Pierce™ BCA protein assay reagents A and B. A volume of protein extract corresponding to 5 μg of protein was loaded onto 12% SDS – polyacrylamide gels and separated by electrophoresis. The gels were then transferred to PVDF membranes, which were blocked with 5% skim milk in PBS-Tween-20 (0.1%) for 1 h at room temperature. Subsequently, the membranes were incubated with the primary antibodies for 30 mi at room temperature (actin) or overnight at 4 °C (CDK4, CDK6, cyclin A, Rb and SOD2/MnSOD). After incubation, the membranes were washed with PBS-Tween-20 (0.1%) and incubated with anti-mouse secondary antibody (40 min) or anti-rabbit secondary antibody (1 h) at room temperature. Finally, the membranes were washed with PBS-Tween-20 (0.1%) and treated with the Immobilon Western Chemiluminescent HRP Substrate Kit, and autoradiography was performed using an FPM-100A film processor (Fujifilm, Tokyo, Japan). western blot results were obtained from three independent experiments, except for cyclin A at 24 h (*n* = 2), Rb protein at 24 and 72 h (*n* = 2), and SOD2 (MnSOD) at 72 h (*n* = 2).

#### Statistical analysis

The results are expressed as mean ± SD of at least three independent experiments, unless otherwise specified. Each condition (control or treatment) was performed in triplicate. Statistical analyses were performed using the GraphPad Prism 9 software (GraphPad Software, San Diego, CA, USA). The means between the control and treated cells were compared using multiple *t*-tests (one independent *t*-test per row) or two-way ANOVA (Sidak's *post hoc* comparison test). The means were statistically different at *p* < 0.05 (*), *p* < 0.01 (**) or *p* < 0.001 (***).

### Molecular docking studies

The structures of CDK6 and compound 17 were prepared and validated using the same methodology described in our previous study.^20^ Furthermore, selection of the optimal protocol, scoring function, and software was also conducted using the same procedure described in that study.

## Conclusions

In conclusion, we successfully synthesized novel CA 1 derivatives featuring ester or carbamate groups at C-20, and derivatives containing these functional groups along with carbonyl or hydroxyl groups at C-7. The evaluation of these compounds in HCT116 cells demonstrated that introducing a butyl ester at C-20 in combination with a carbonyl group at C-7 (compound 8, IC_50_ = 15.1 μM) or introducing a 2-methylpropyl carbamate at C-20 (compound 17, IC_50_ = 17 μM) contributed to the increase in antiproliferative activity relative to CA 1 (IC_50_ = 42 μM). Overall, compound 17 displayed the highest potency against other cancer cell lines, achieving the best results against SW480 cells with an IC_50_ of 6.3 μM. Moreover, this compound was less potent in inhibiting the growth of BJ fibroblasts than the growth of cancer cells, with an IC_50_ of > 50 μM, showing selectivity towards cancer cells. Additional studies revealed that compound 17 arrested the cell cycle and modulated oxidative stress in a time-dependent manner in SW480 cells. At 24 h, compound 17 arrested the cell cycle in the G0/G1 phase by downregulating CDK4 and CDK6 in SW480 cells, and increased SOD2 (MnSOD) levels, leading to a reduction in ROS levels. At 48 h, compound 17 induced cell cycle arrest in the S phase and increased ROS levels. After 72 h of treatment, the pro-oxidant effect was maintained. Furthermore, molecular docking studies showed that compound 17 established several interactions with amino acids of the CDK6 active site, suggesting its potential as a CDK6 inhibitor. Our findings suggest that compound 17 is a promising candidate for the development of novel anticancer agents, and warrants further research.

## Author contributions

Sara P. S. P. Moura: conceptualization, methodology, validation, formal analysis, investigation, data curation, writing – original draft preparation, visualization. Marta Cascante: conceptualization, ethodology, validation, resources, writing – review and editing, supervision, project administration, funding acquisition. Ismael Rufino: methodology, formal analysis, investigation, data curation, writing – original draft preparation, visualization. Rita C. Guedes: conceptualization, methodology, validation, resources, writing – review and editing, supervision, project administration, funding acquisition. Silvia Marin: conceptualization, methodology, validation, formal analysis, writing – review and editing, supervision. Jorge A. R. Salvador: conceptualization, methodology, validation, resources, writing – review and editing, supervision, project administration, funding acquisition.

## Conflicts of interest

There are no conflicts to declare.

## Supplementary Material

RA-015-D5RA02441B-s001

RA-015-D5RA02441B-s002

## Data Availability

Raw data files supporting the western blot experiments are available at Zenodo at https://doi.org/10.5281/zenodo.15577318. Supplementary information: Selected spectroscopic information, dose–response curves, and additional molecular docking results. See DOI: https://doi.org/10.1039/d5ra02441b.
