# Supplementary material for: Synthesis and biological evaluation of novel carnosic acid derivatives with anticancer activity
Source: RSC Adv. 2025 Oct 6;15(44):36861–78. doi: 10.1039/d5ra02441b (PMC12498136; doi:10.1039/d5ra02441b)
Supplement: RA-015-D5RA02441B-s001 [file RA-015-D5RA02441B-s001.pdf]

Supplementary Information (SI) for RSC Advances.
This journal is © The Royal Society of Chemistry 2025

Unprocessed images of western blot of Figure **6**:

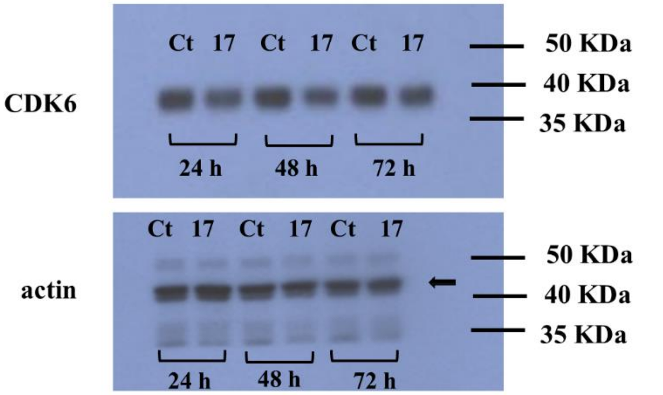

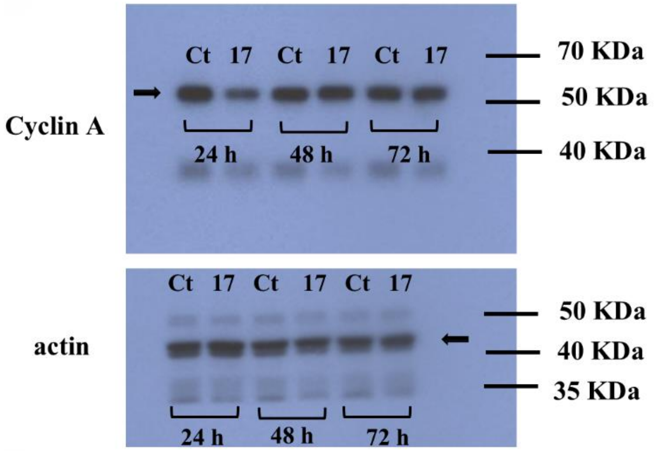

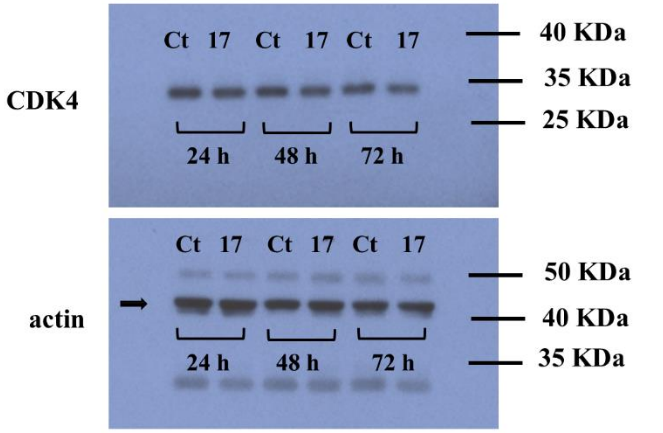

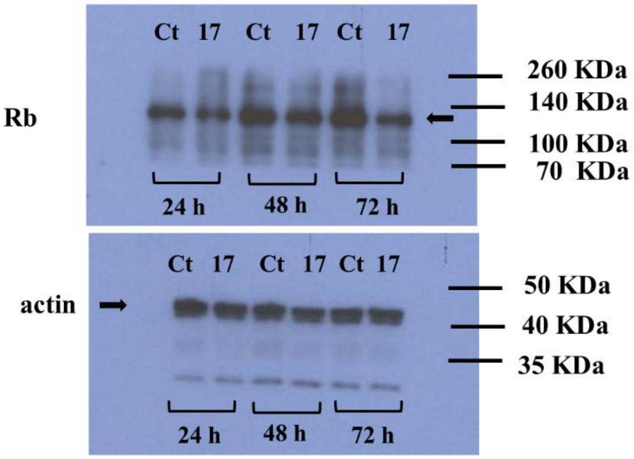

Unprocessed images of western blot of Figure **9**:

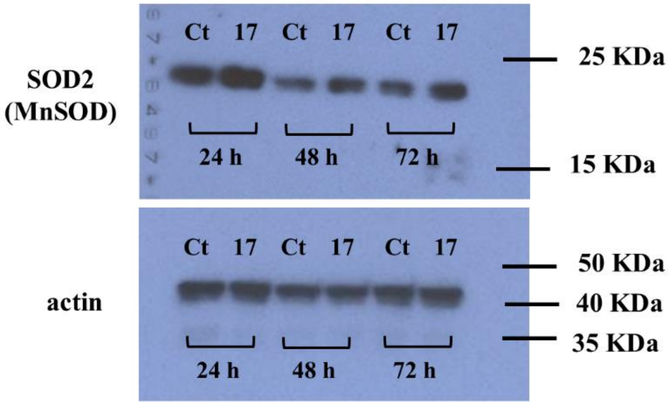

Images of the western blots of independent biological replicates used in the quantitative analysis are presented below:

- **CDK6**

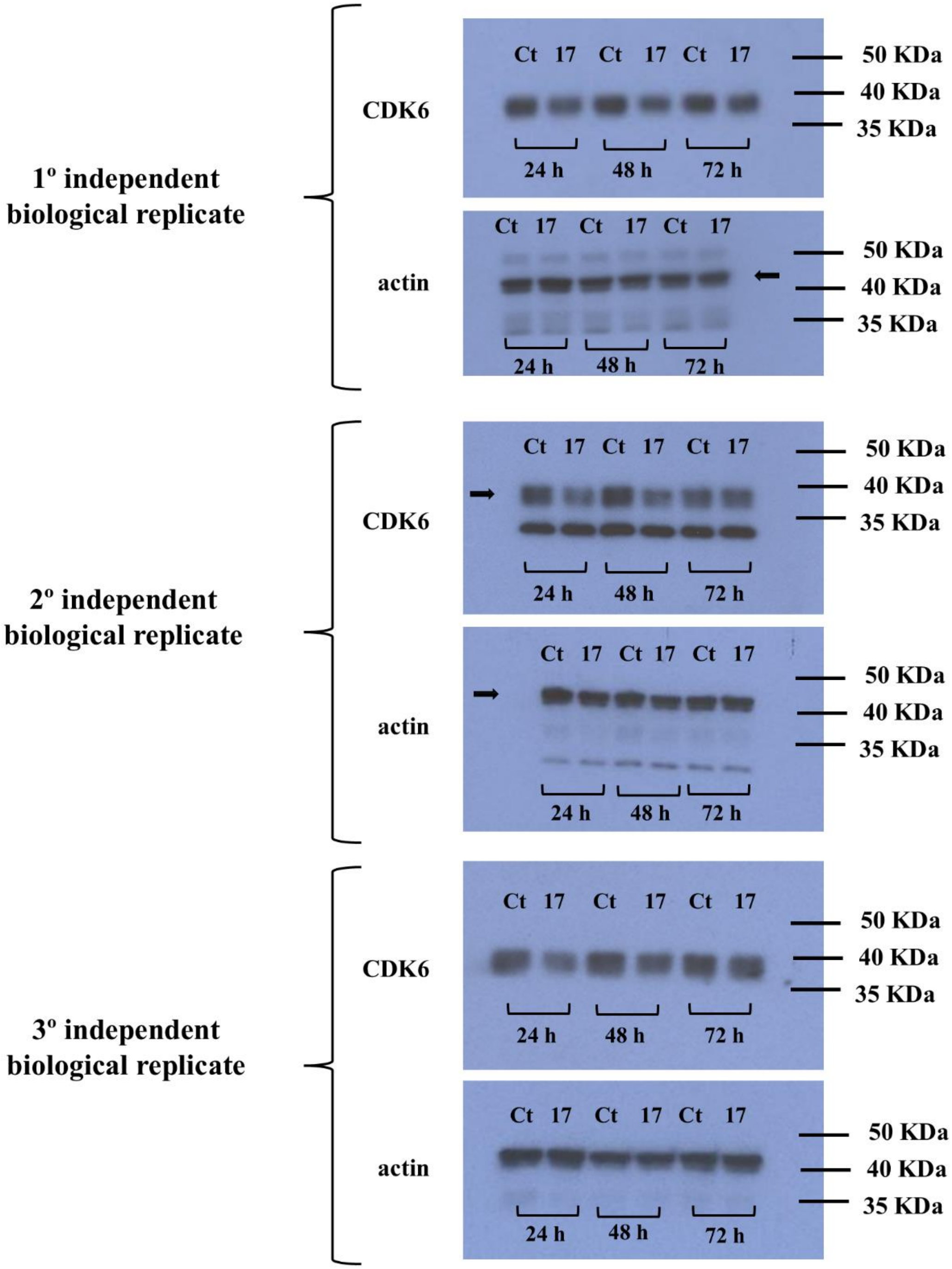

Note: For membranes containing multiple bands, an arrow marks the band of interest.

- **Cyclin A**

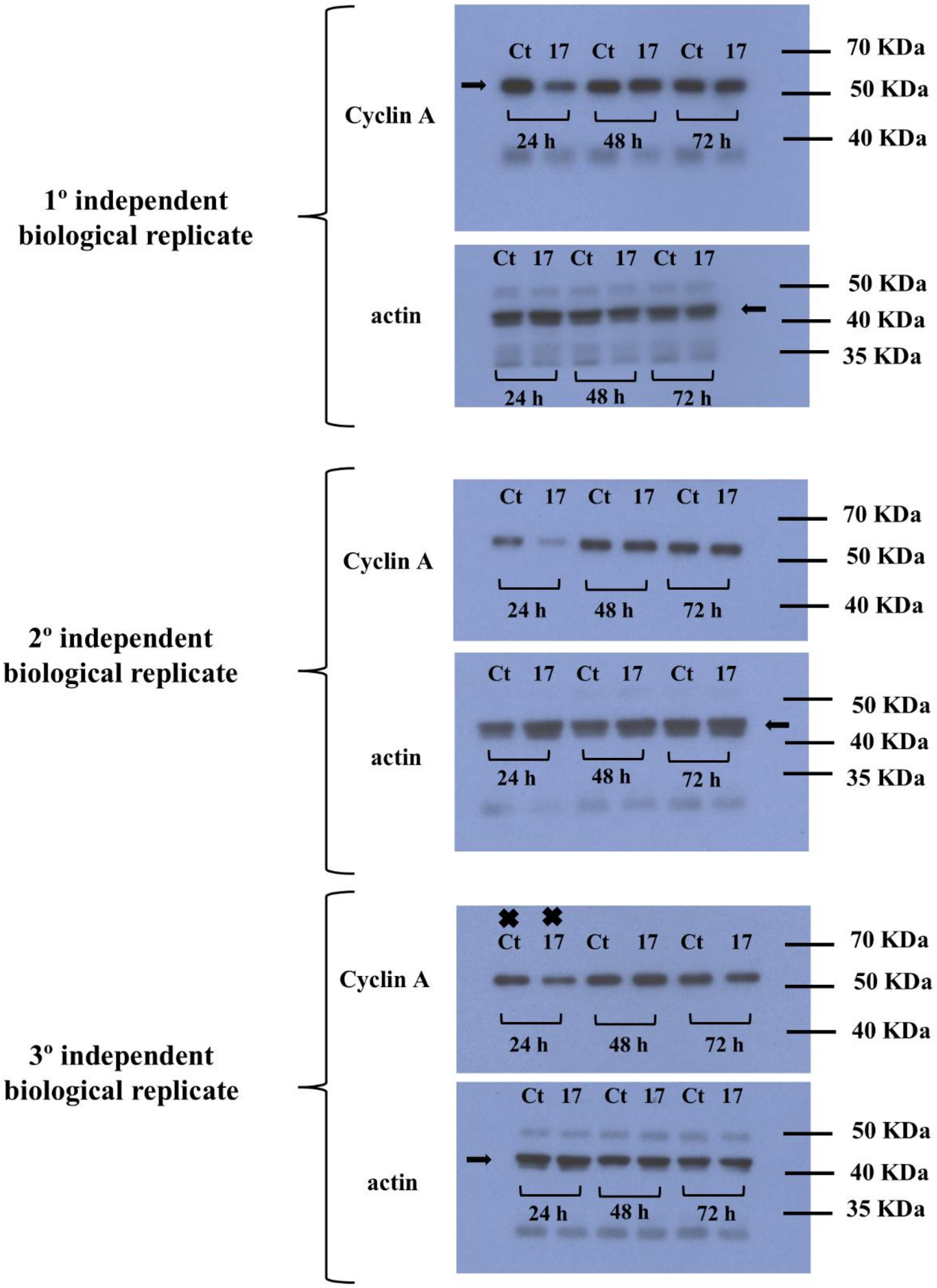

Note: For membranes containing multiple bands, an arrow marks the band of interest. The lanes marked with an "X" above the lane label on the blot image were not included in the quantitative analysis.

- **CDK4**

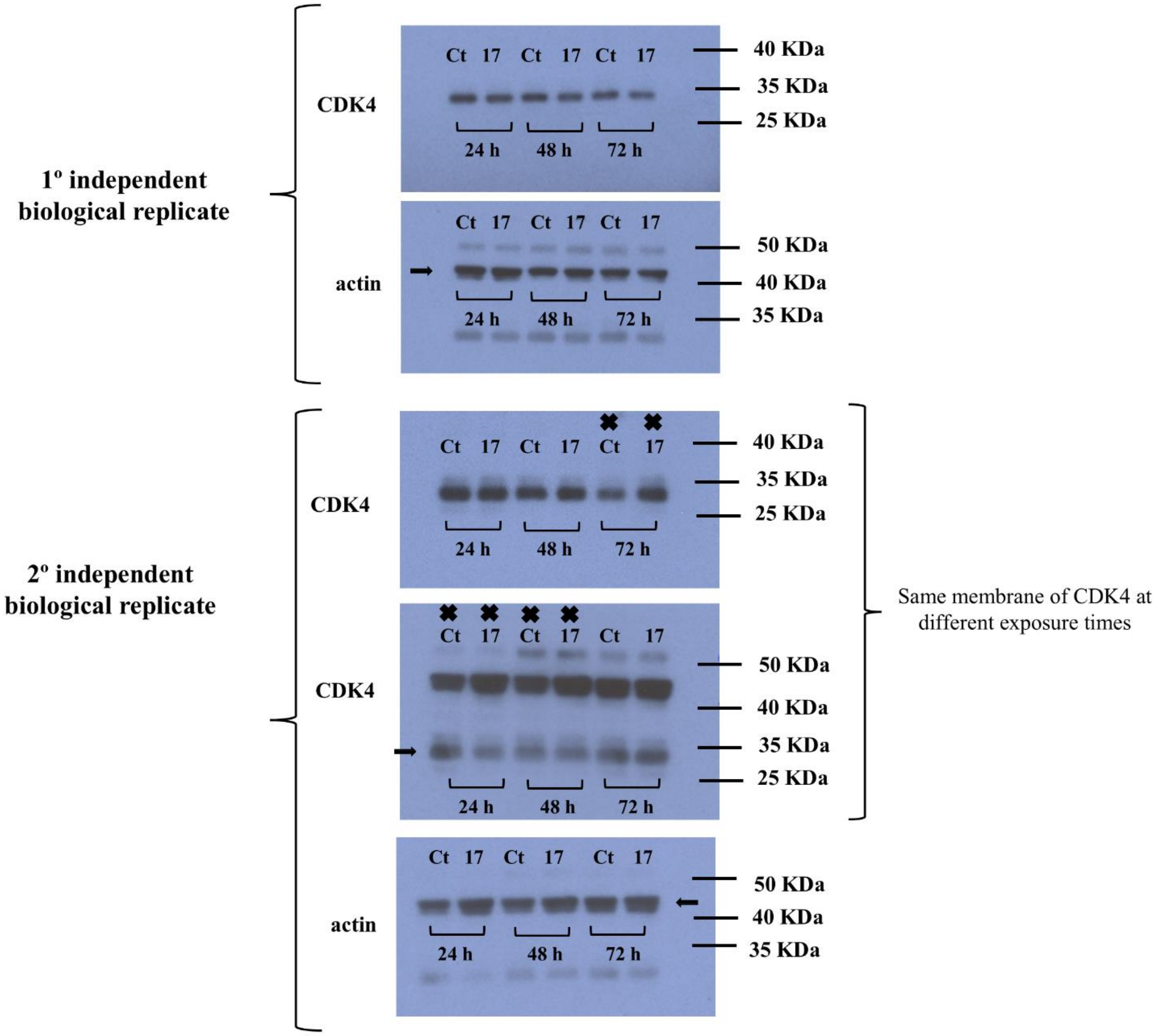
1º independent biological replicate
CDK4
Ct 17 Ct 17 Ct 17
24 h 48 h 72 h
40 KDa
35 KDa
25 KDa
actin
50 KDa
40 KDa
35 KDa
2º independent biological replicate
CDK4
CDK4
actin
Same membrane of CDK4 at different exposure times

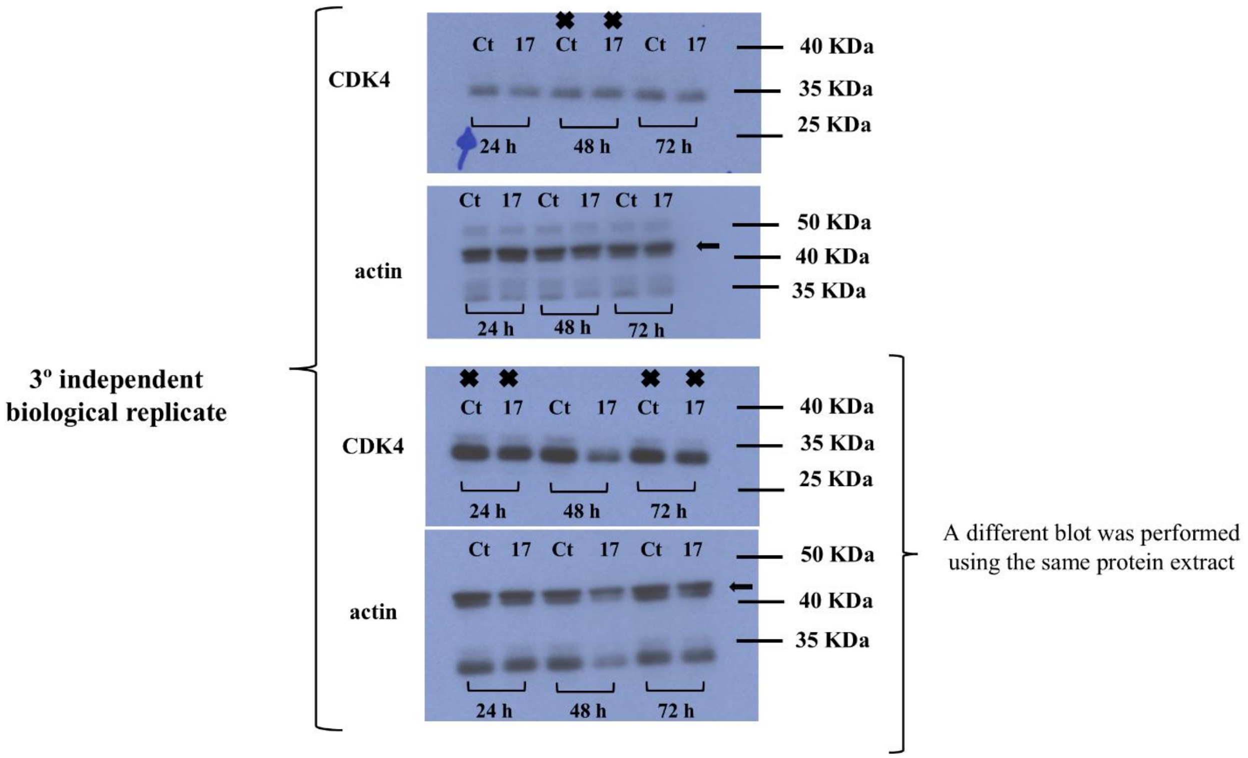

Note: For membranes containing multiple bands, an arrow marks the band of interest. The lanes marked with an “X” above the lane label on the blot image were not included in the quantitative analysis.

- **Rb**

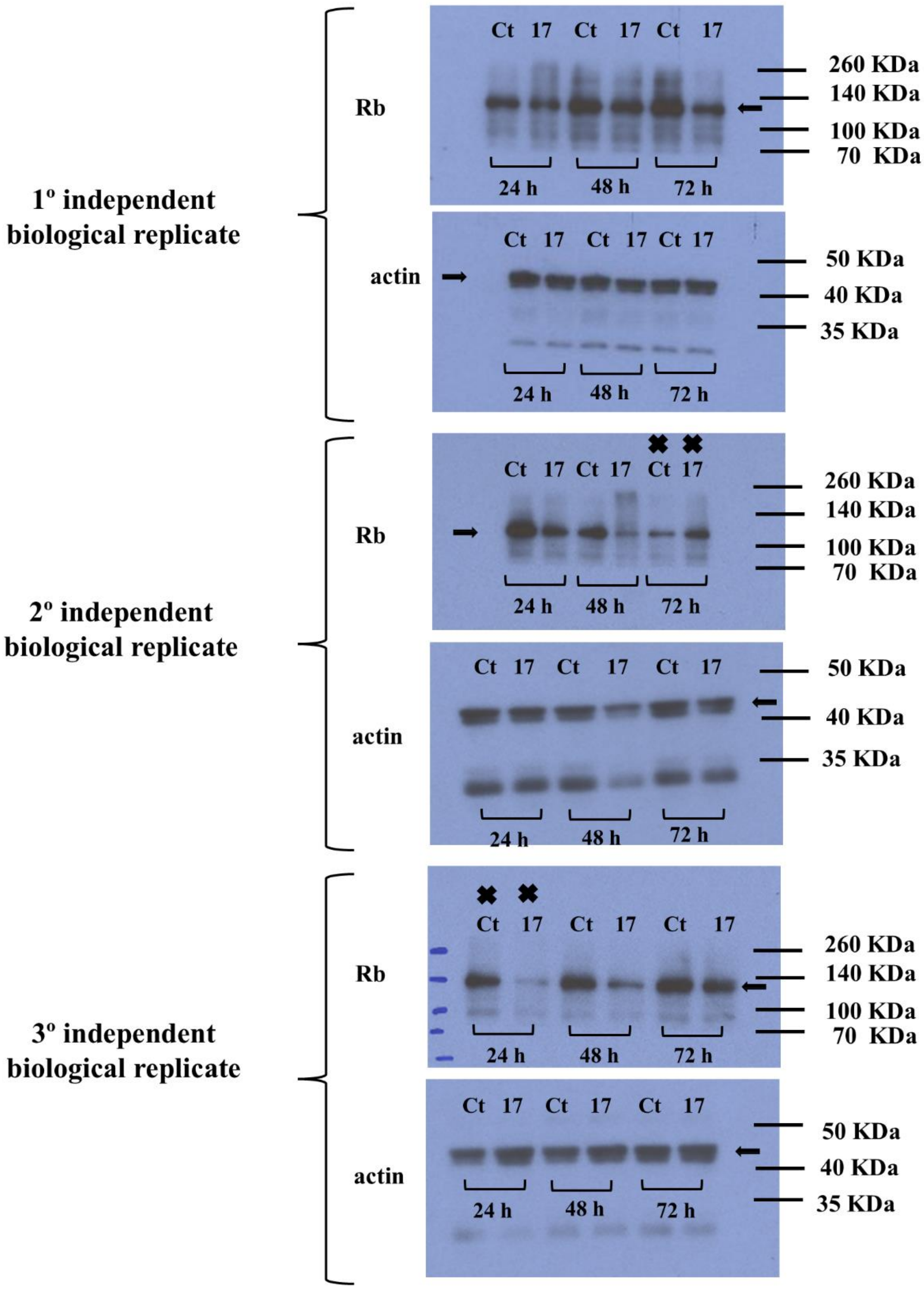

Note: For membranes containing multiple bands, an arrow marks the band of interest. The lanes marked with an “X” above the lane label on the blot image were not included in the quantitative analysis.

- **SOD2 (MnSOD)**

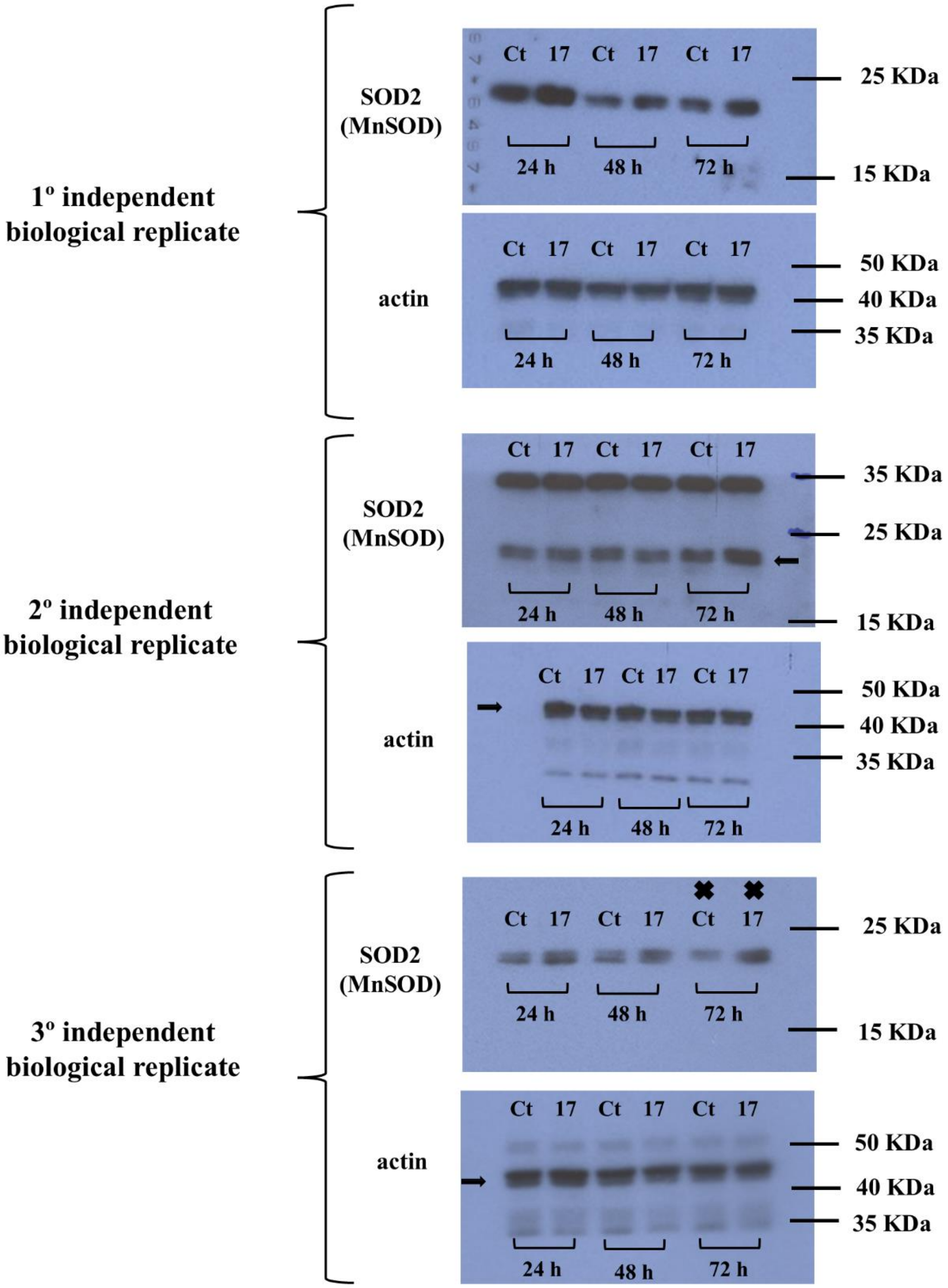

Note: For membranes containing multiple bands, an arrow marks the band of interest. The lanes marked with an “X” above the lane label on the blot image were not included in the quantitative analysis.
