# Supplementary material for: Synthesis and biological evaluation of novel carnosic acid derivatives with anticancer activity
Source: RSC Adv. 2025 Oct 6;15(44):36861–78. doi: 10.1039/d5ra02441b (PMC12498136; doi:10.1039/d5ra02441b)
Supplement: RA-015-D5RA02441B-s002 [file RA-015-D5RA02441B-s002.pdf]

Supplementary Information (SI) for RSC Advances.
This journal is © The Royal Society of Chemistry 2025

Supplementary Information

Synthesis and biological evaluation of novel carnosic acid derivatives with anticancer activity

Sara P. S. P. Moura,[a, b, c] Marta Cascante,[d, e] Ismael Rufino,[f] Rita C. Guedes,[f] Silvia Marín,[*d, e] and Jorge A. R. Salvador[*a, b, c]

[a] *Laboratory of Pharmaceutical Chemistry, Faculty of Pharmacy, University of Coimbra, 3000-548 Coimbra, Portugal. Tel: +351-239-488-479; E-mail: salvador@ci.uc.pt*

[b] *Center for Neuroscience and Cell Biology (CNC), University of Coimbra, 3004-504 Coimbra, Portugal.*

[c] *Centre for Innovative Biomedicine and Biotechnology (CIBB), University of Coimbra, 3004-504 Coimbra, Portugal.*

[d] *Department of Biochemistry and Molecular Biomedicine, Faculty of Biology, University of Barcelona, 08028 Barcelona, Spain. Tel: +34-934-9683; E-mail: silviamarin@ub.edu*

[e] *Centro de Investigación Biomédica en Red de Enfermedades Hepáticas y Digestivas (CIBEREHD), Instituto de Salud Carlos III (ISCIII), 28029 Madrid, Spain.*

[f] *Research Institute for Medicines (iMed.ULisboa), Faculty of Pharmacy, University of Lisboa, 1649-003 Lisboa, Portugal.*

Table of Contents

1. NMR data for compound 2 5

1.1. ^{1}H spectrum for compound **2** recorded in $CDCl_3$ 5

1.2. ^{13}C spectrum for compound **2** recorded in $CDCl_3$ 6

2. NMR data for compound 3 7

2.1. ^{1}H spectrum for compound **3** recorded in $CDCl_3$ 7

2.2. ^{13}C spectrum for compound **3** recorded in $CDCl_3$ 8

3. NMR data for compound 4 9

3.1. ^{1}H spectrum for compound **4** recorded in $CDCl_3$ 9

3.2. ^{13}C spectrum for compound **4** recorded in $CDCl_3$ 10

4. NMR data for compound 5 11

4.1. ^{1}H spectrum for compound **5** recorded in $CDCl_3$ 11

4.2. ^{13}C spectrum for compound **5** recorded in $CDCl_3$ 12

5. NMR data for compound 6 13

5.1. ^{1}H spectrum for compound **6** recorded in $CDCl_3$ 13

5.2. ^{13}C spectrum for compound **6** recorded in $CDCl_3$ 14

6. NMR data for compound 7 15

6.1. ^{1}H spectrum for compound **7** recorded in $CDCl_3$ 15

6.2. ^{13}C spectrum for compound **7** recorded in $CDCl_3$ 16

7. NMR data for compound 8 17

7.1. ^{1}H spectrum for compound **8** recorded in $CDCl_3$ 17

7.2. ^{13}C spectrum for compound **8** recorded in $CDCl_3$ 18

8. NMR data for compound 9 19

8.1. ^{1}H spectrum for compound **9** recorded in $CDCl_3$ 19

8.2. ^{13}C spectrum for compound **9** recorded in $CDCl_3$ 20

9. NMR data for compound 10 21

9.1. ^{1}H spectrum for compound **10** recorded in $CDCl_3$ 21

9.2. ^{13}C spectrum for compound **10** recorded in $CDCl_3$ 22

9.3. NOESY spectrum for compound **10** recorded in $CDCl_3$ 23

9.4. HSQC spectrum for compound **10** recorded in $CDCl_3$ 24

9.5. HMBC spectrum for compound **10** recorded in $CDCl_3$ 25
10. NMR data for compound 11 26
10.1. ^{1}H spectrum for compound **11** recorded in $CDCl_3$ 26
10.2. ^{13}C spectrum for compound **11** recorded in $CDCl_3$ 27
11. NMR data for compound 14 28
11.1. ^{1}H spectrum for compound **14** recorded in $CDCl_3$ 28
11.2. ^{13}C spectrum for compound **14** recorded in $CDCl_3$ 29
12. NMR data for compound 15 30
12.1. ^{1}H spectrum for compound **15** recorded in $CDCl_3$ 30
12.2. ^{13}C spectrum for compound **15** recorded in $CDCl_3$ 31
13. NMR data for compound 16 32
13.1. ^{1}H spectrum for compound **16** recorded in $CDCl_3$ 32
13.2. ^{13}C spectrum for compound **16** recorded in $CDCl_3$ 33
13.3. COSY spectrum for compound **16** recorded in $CDCl_3$ 34
13.4. HSQC spectrum for compound **16** recorded in $CDCl_3$ 35
13.5. HMBC spectrum for compound **16** recorded in $CDCl_3$ 36
14. NMR data for compound 17 37
14.1. ^{1}H spectrum for compound **17** recorded in $CDCl_3$ 37
14.2. ^{13}C spectrum for compound **17** recorded in $CDCl_3$ 38
14.3. COSY spectrum for compound **17** recorded in $CDCl_3$ 39
14.4. HSQC spectrum for compound **17** recorded in $CDCl_3$ 40
14.5. HMBC spectrum for compound **17** recorded in $CDCl_3$ 41
15. NMR data for compound 18 42
15.1. ^{1}H spectrum for compound **18** recorded in $CDCl_3$ 42
15.2. ^{13}C spectrum for compound **18** recorded in $CDCl_3$ 43
16. NMR data for compound 19 44
16.1. ^{1}H spectrum for compound **19** recorded in $CDCl_3$ 44
16.2. ^{13}C spectrum for compound **19** recorded in $CDCl_3$ 45
16.3. COSY spectrum for compound **19** recorded in $CDCl_3$ 46
16.4. NOESY spectrum for compound **19** recorded in $CDCl_3$ 47
16.5. HSQC spectrum for compound **19** recorded in $CDCl_3$ 48

16.6. HMBC spectrum for compound **19** recorded in $CDCl_3$ 49
17. Dose-response curves 50
18. Molecular docking studies 56

1. NMR data for compound 2

*1.1. ^{1}H spectrum for compound **2** recorded in $CDCl_3$*

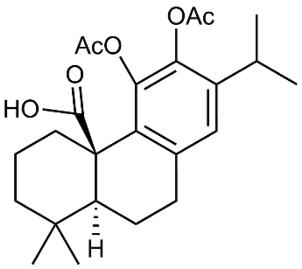

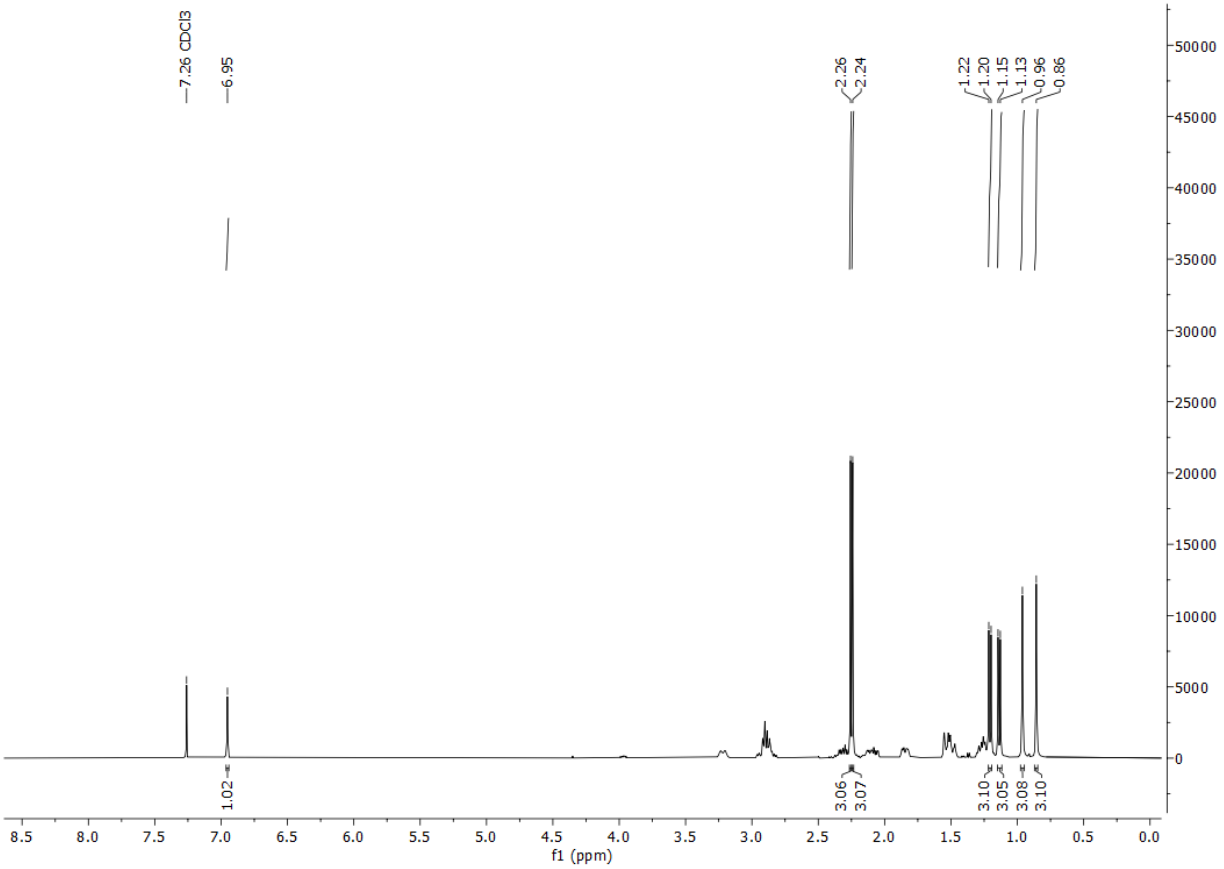

*1.2. ^{13}C spectrum for compound **2** recorded in $CDCl_3$*

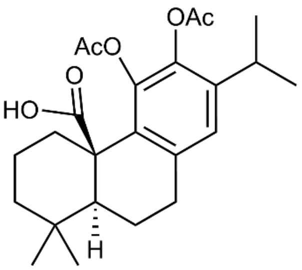

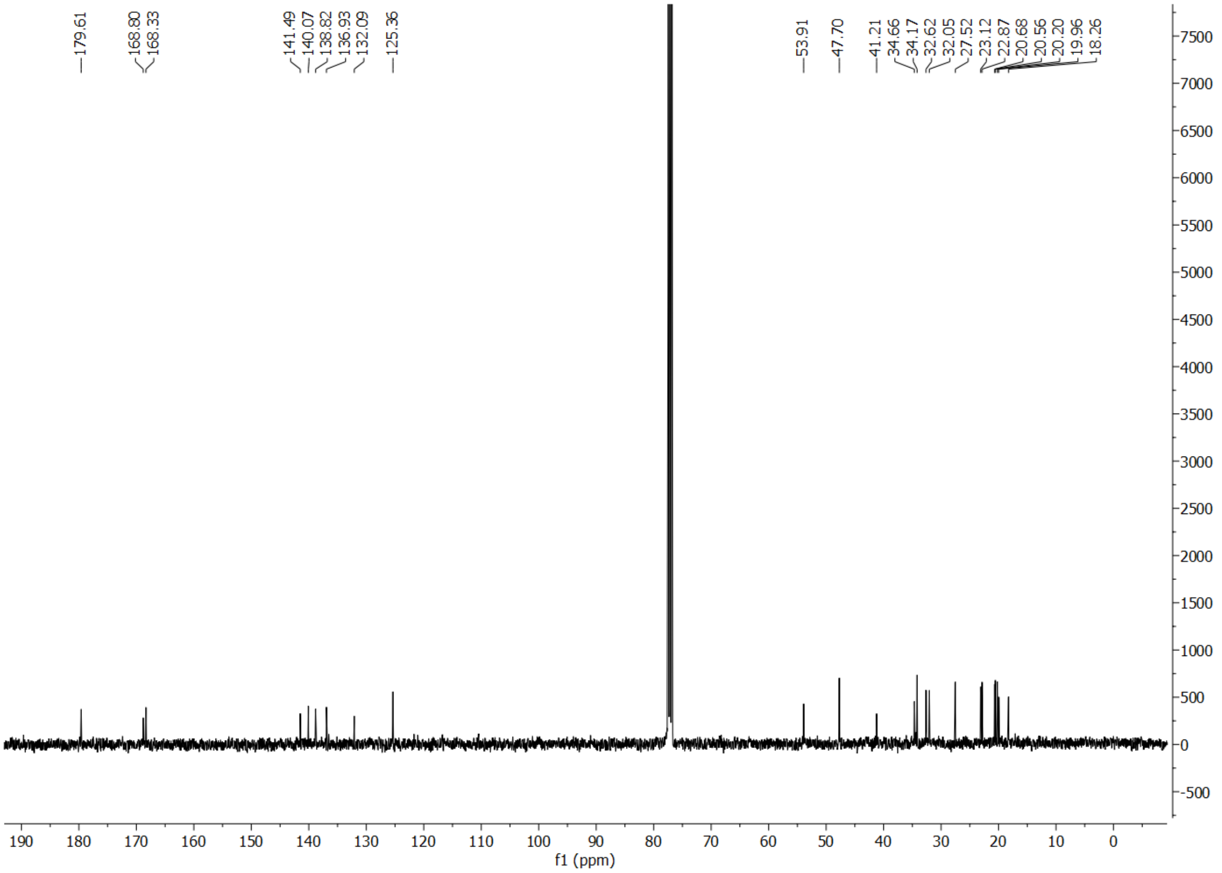

2. NMR data for compound 3

*2.1. ^{1}H spectrum for compound **3** recorded in $CDCl_3$*

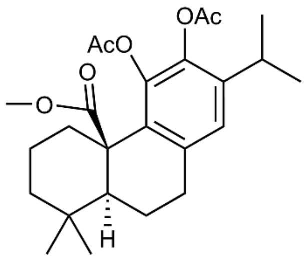

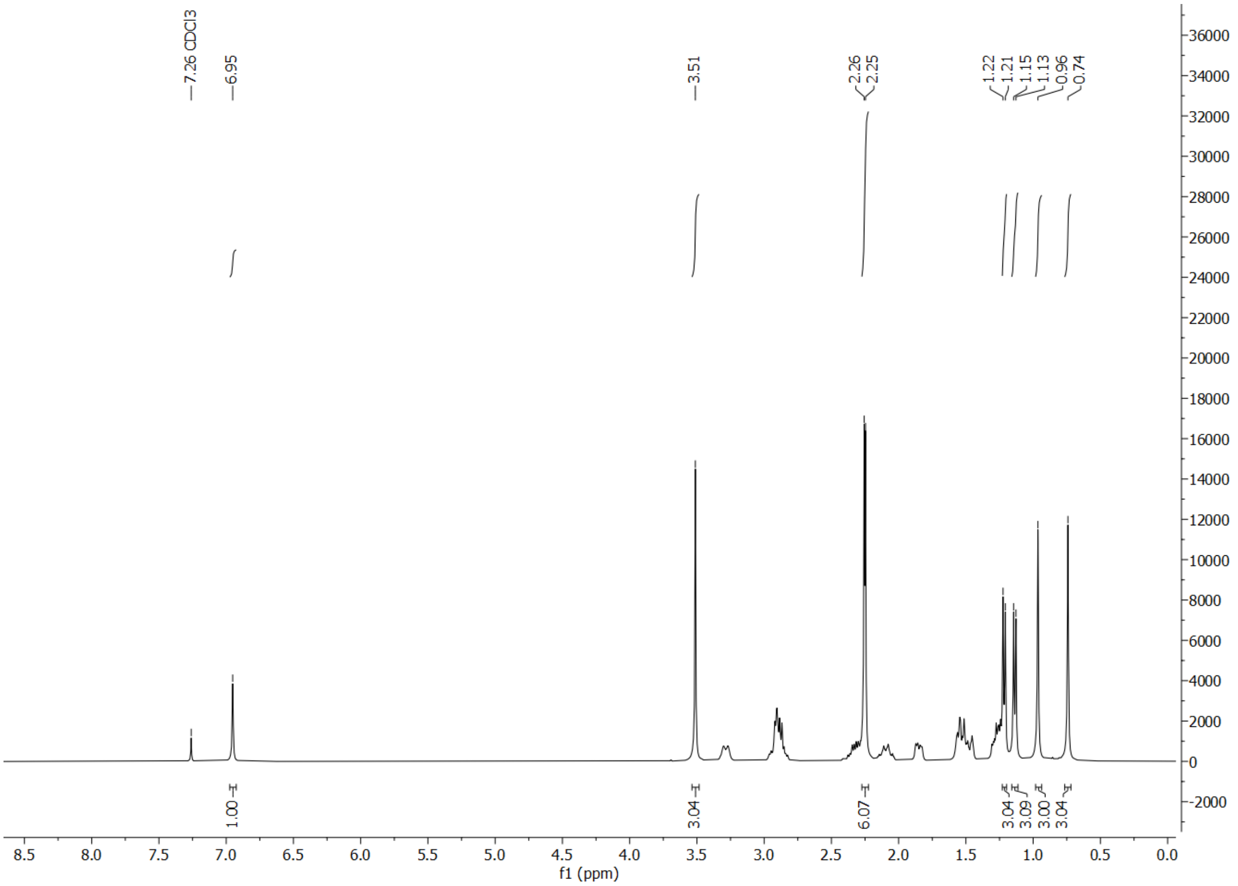

*2.2. ^{13}C spectrum for compound **3** recorded in $CDCl_3$*

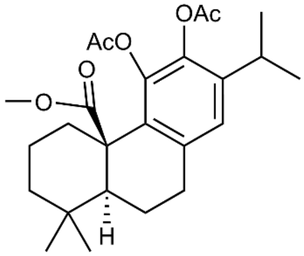

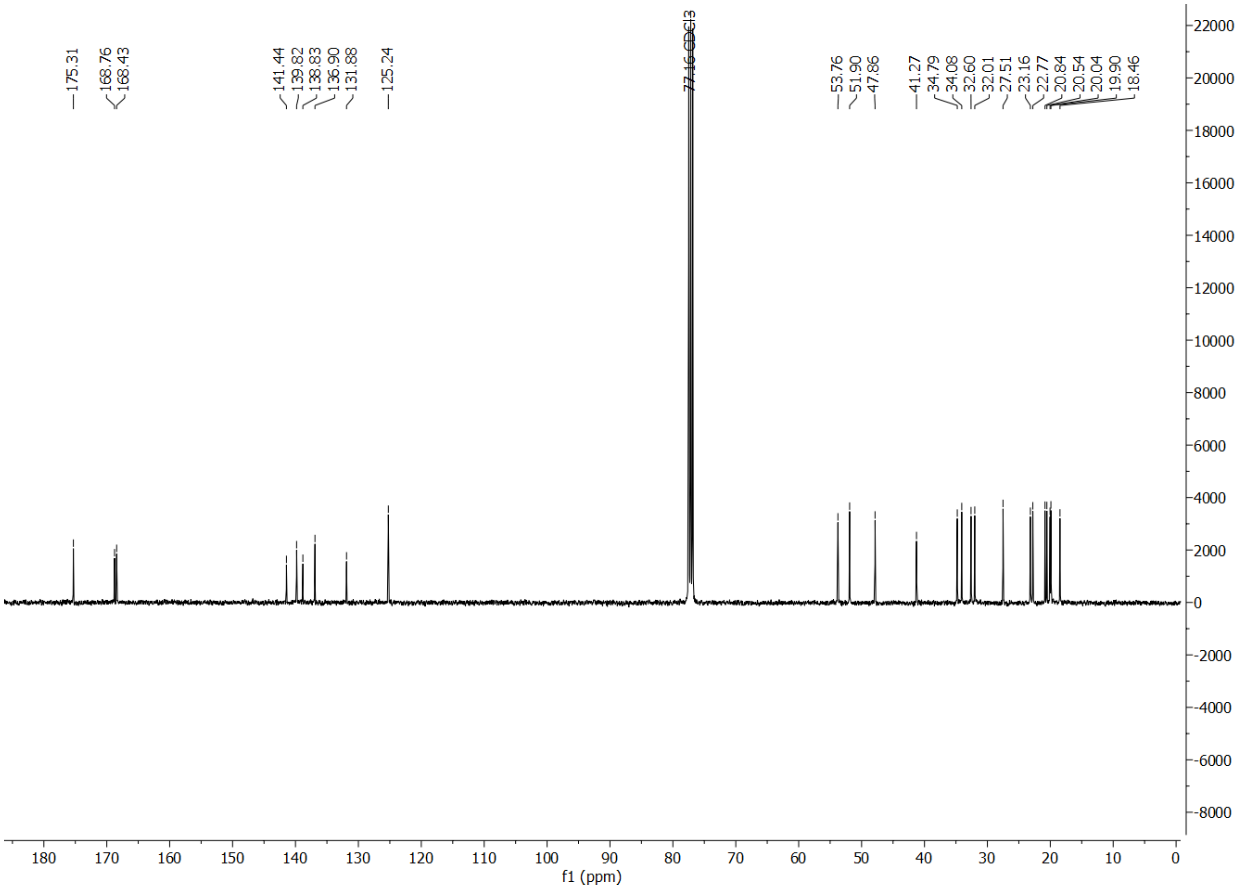

3. NMR data for compound 4

*3.1. ^{1}H spectrum for compound **4** recorded in $CDCl_3$*

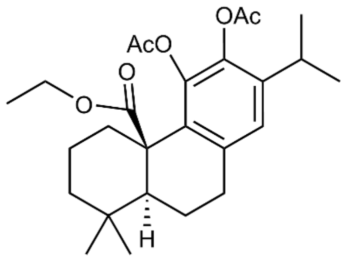

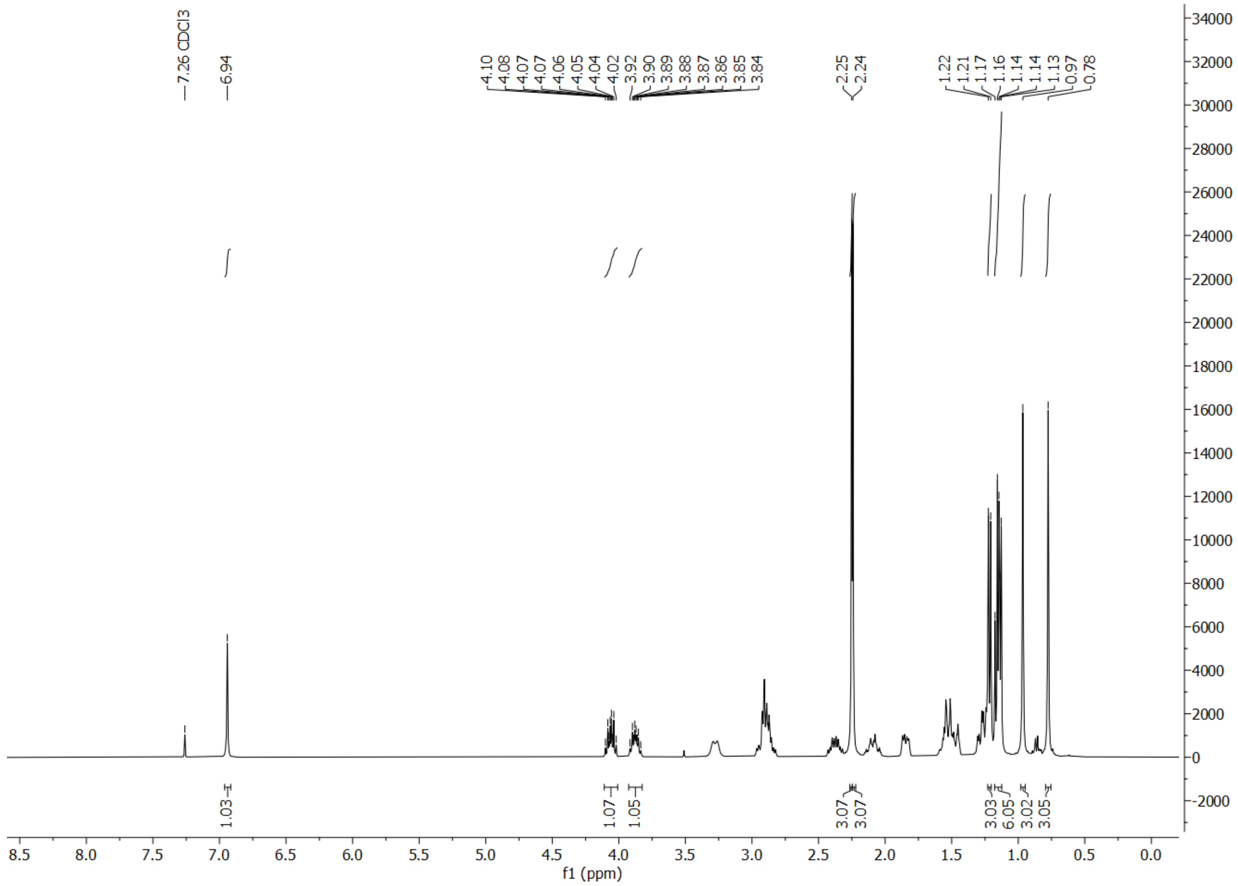

*3.2. ^{13}C spectrum for compound **4** recorded in $CDCl_3$*

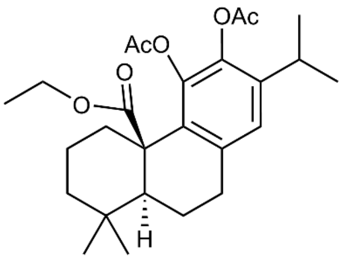

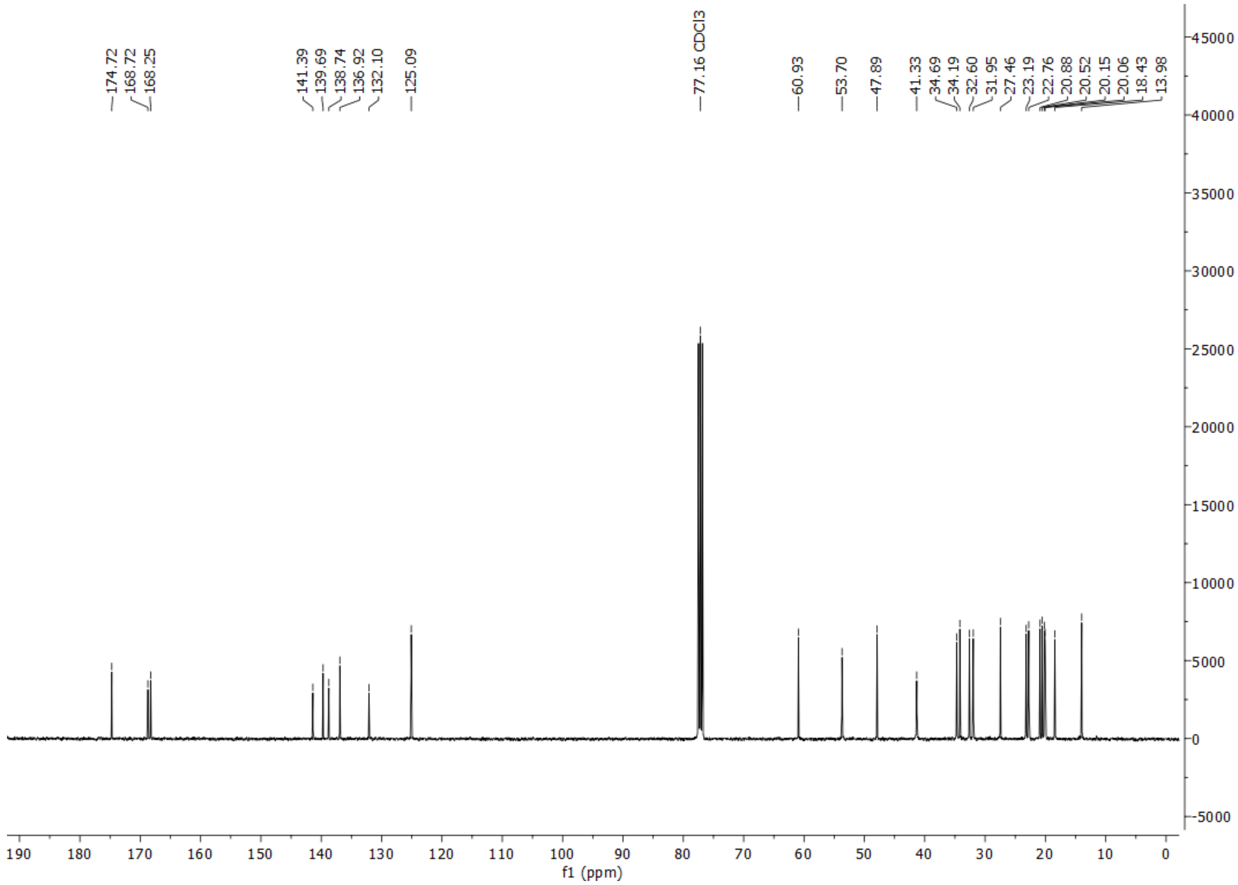

4. NMR data for compound 5

*4.1. ^{1}H spectrum for compound **5** recorded in $CDCl_3$*

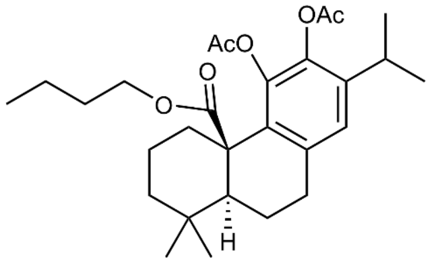

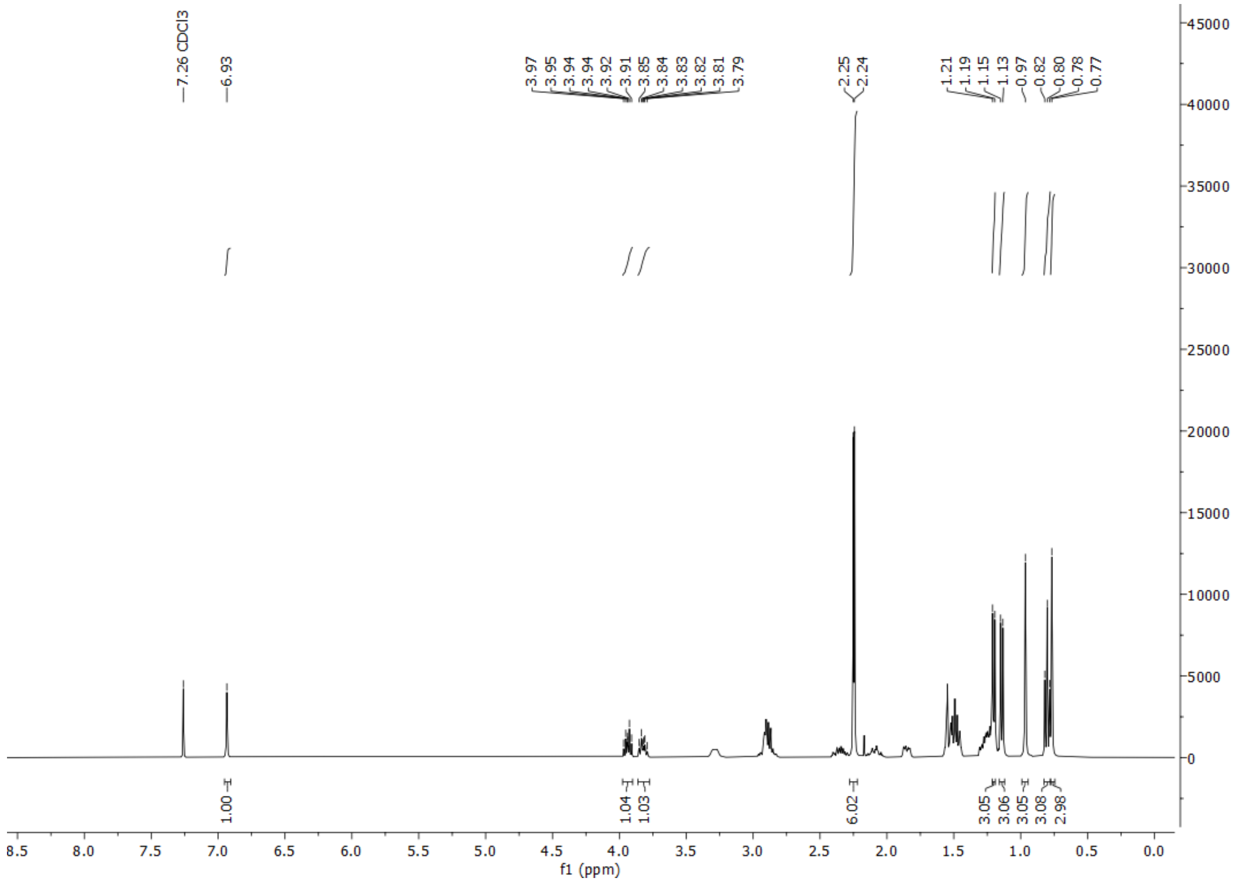

*4.2. ^{13}C spectrum for compound **5** recorded in $CDCl_3$*

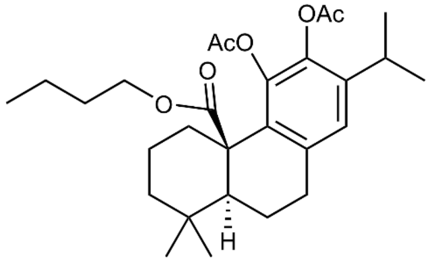

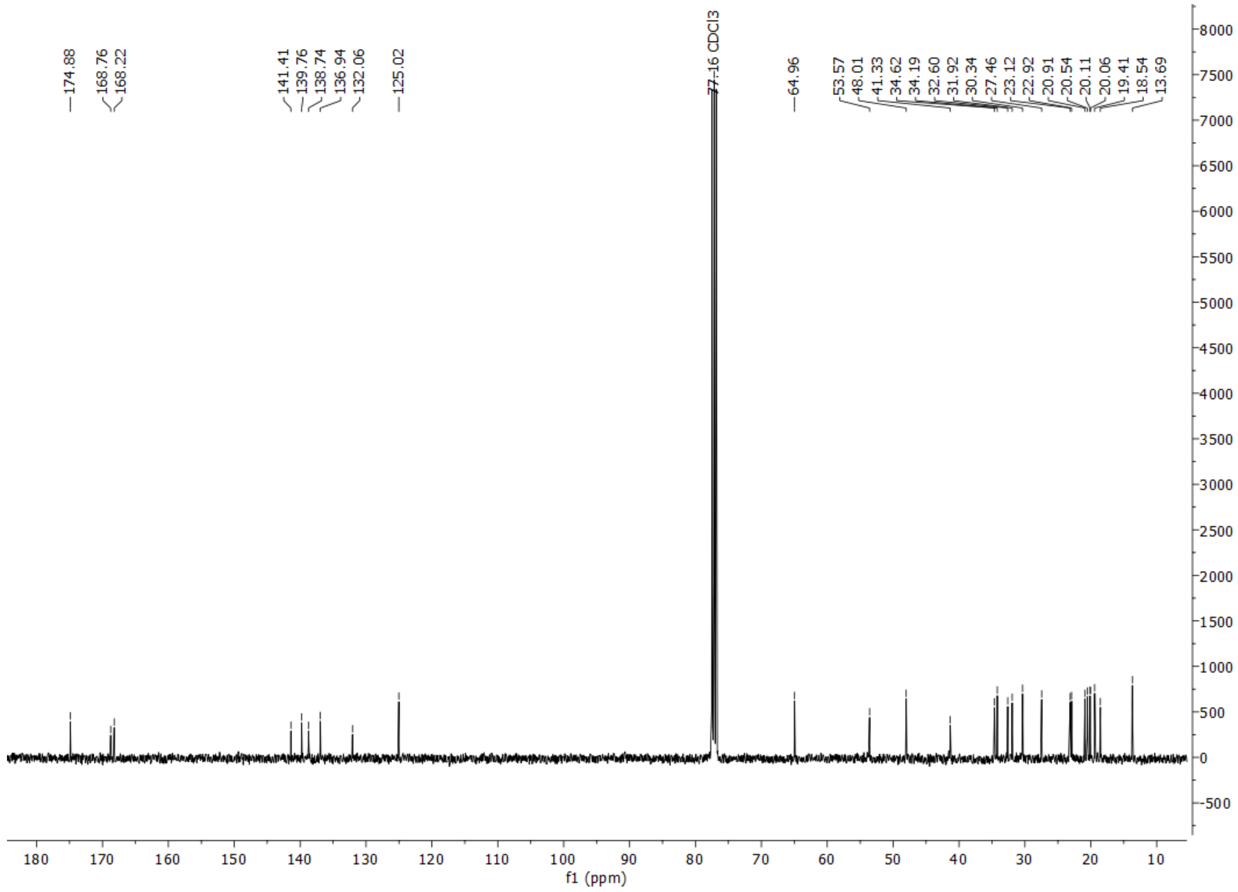

5. NMR data for compound 6

*5.1. ^{1}H spectrum for compound **6** recorded in $CDCl_3$*

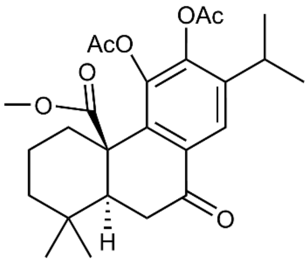

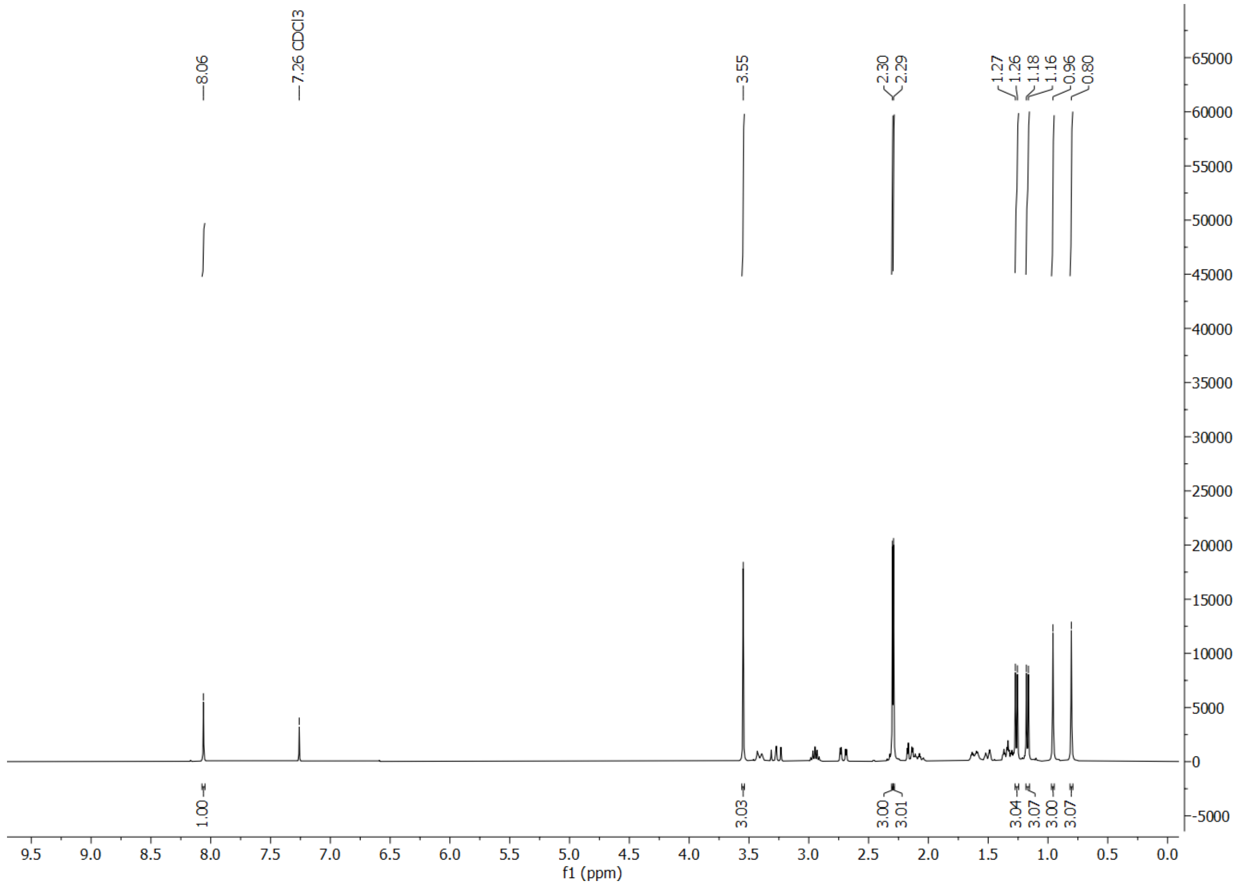

*5.2. ^{13}C spectrum for compound **6** recorded in $CDCl_3$*

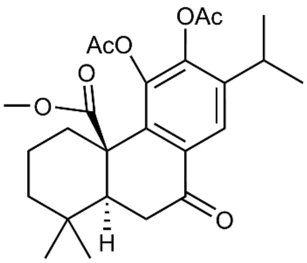

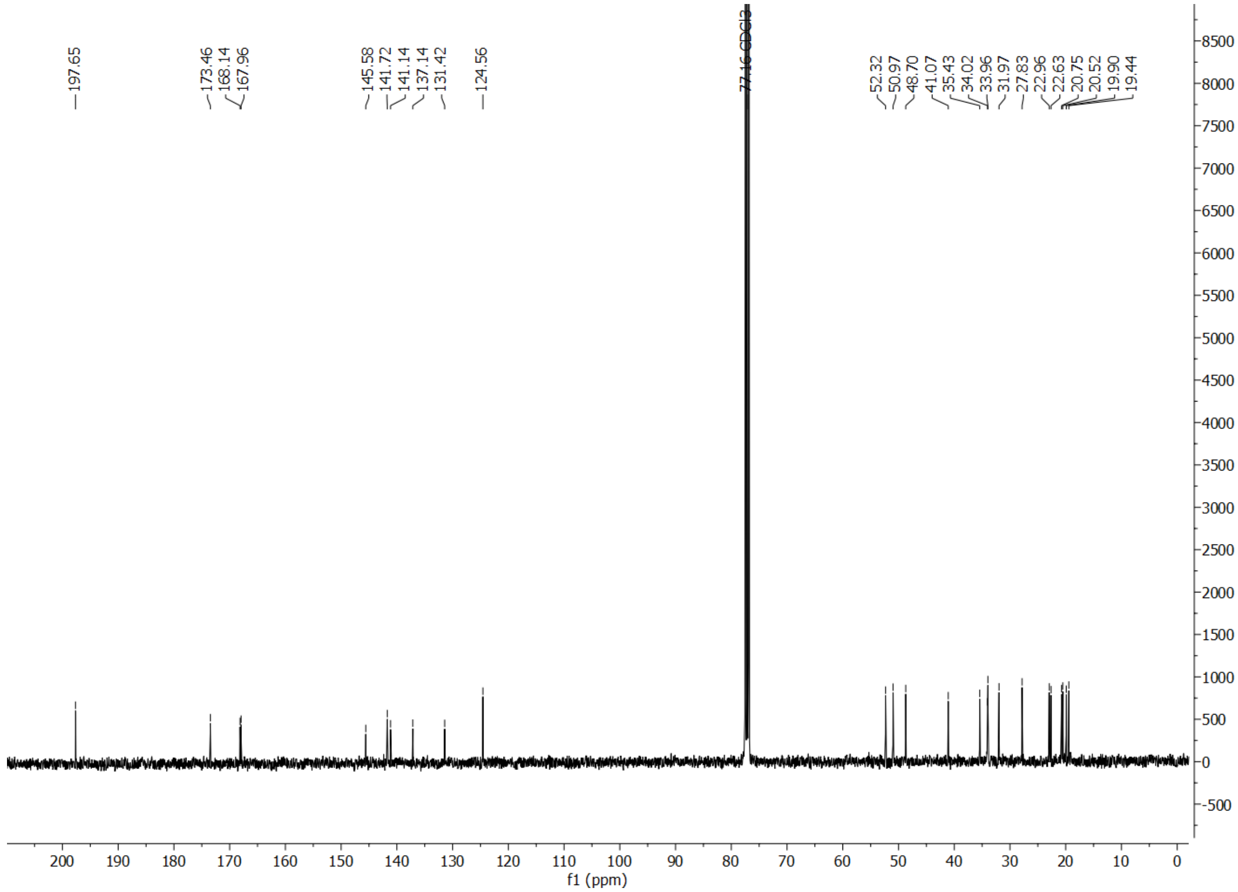

6. NMR data for compound 7

*6.1. ^{1}H spectrum for compound **7** recorded in $CDCl_3$*

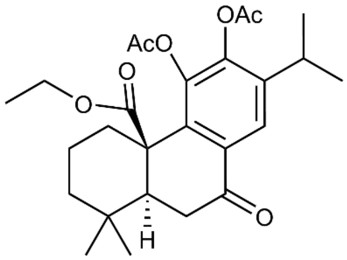

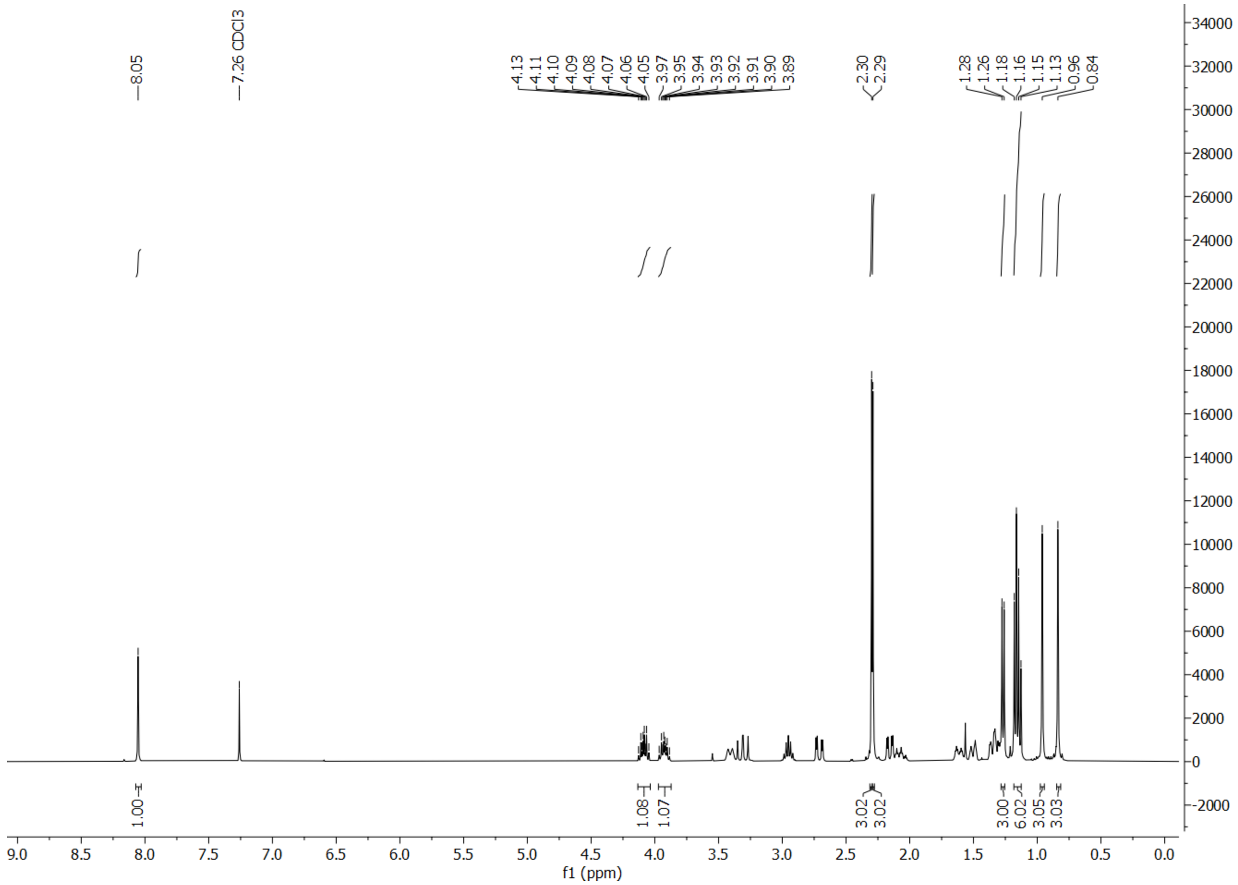

6.2. ¹³C spectrum for compound 7 recorded in $CDCl_3$

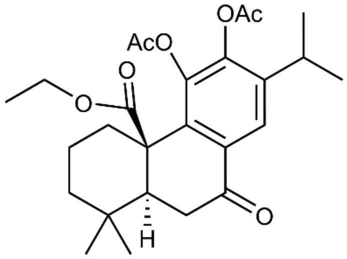

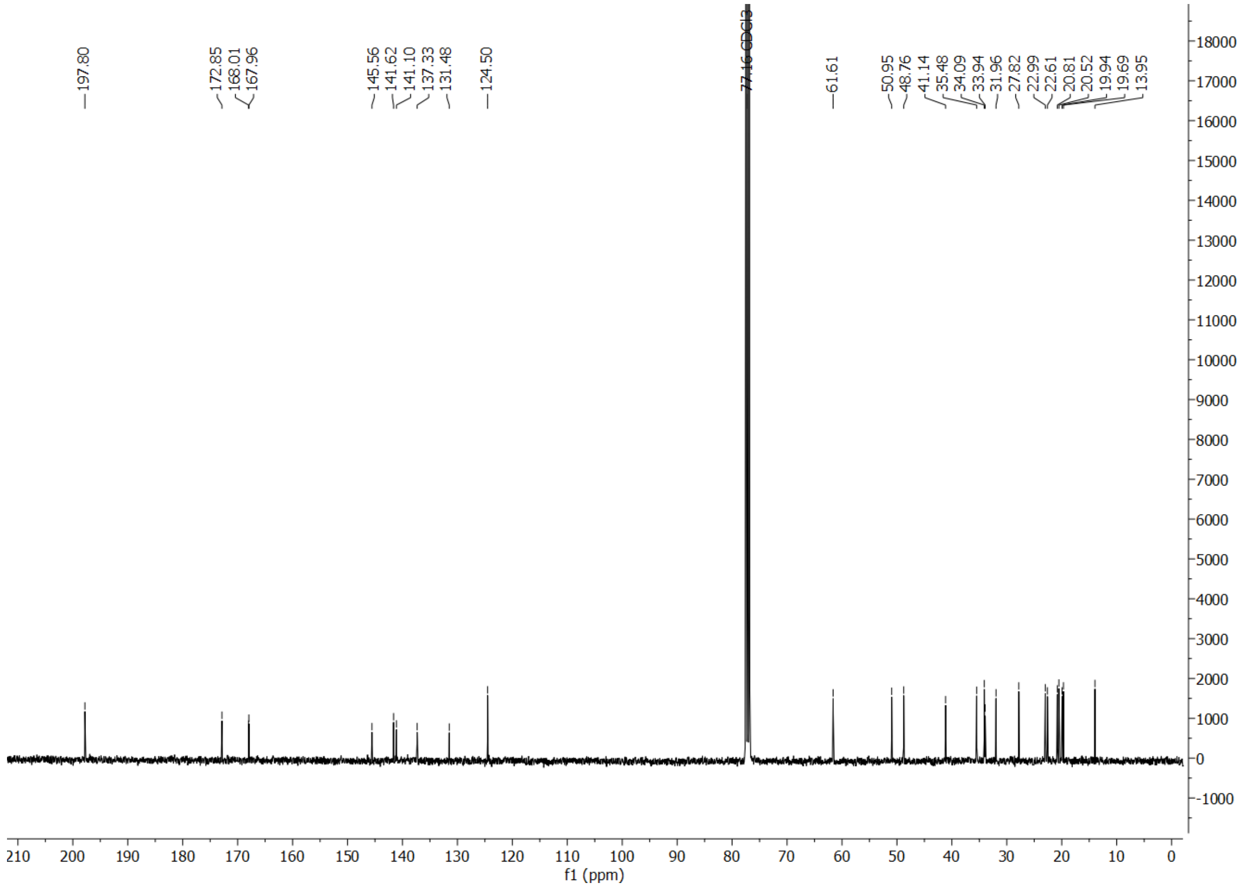

7. NMR data for compound 8

*7.1. ^{1}H spectrum for compound **8** recorded in $CDCl_3$*

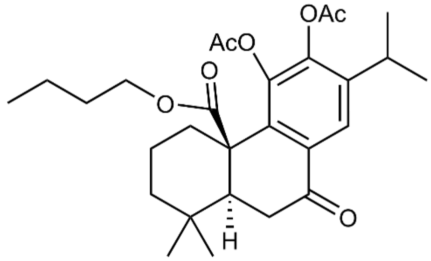

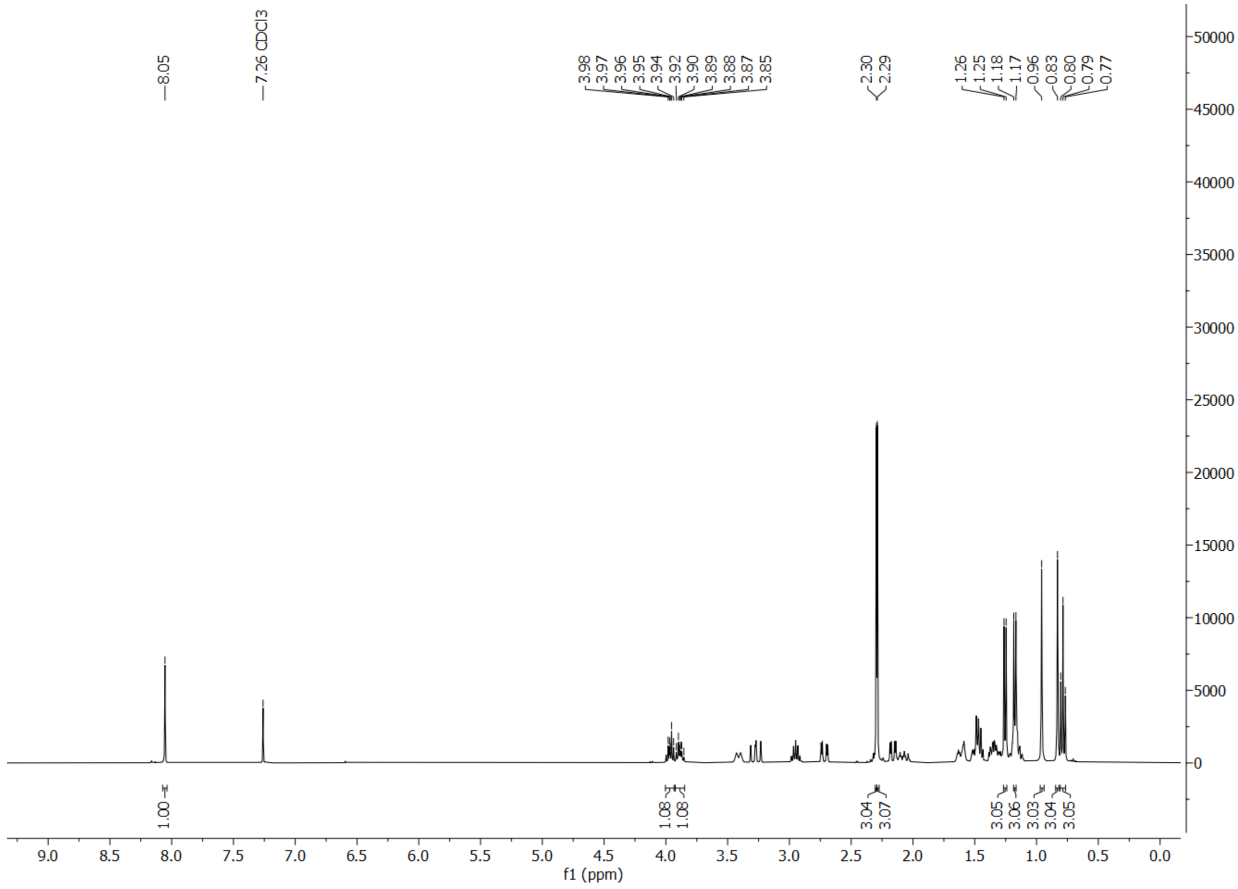

*7.2. ^{13}C spectrum for compound **8** recorded in $CDCl_3$*

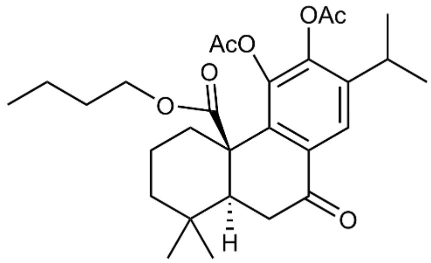

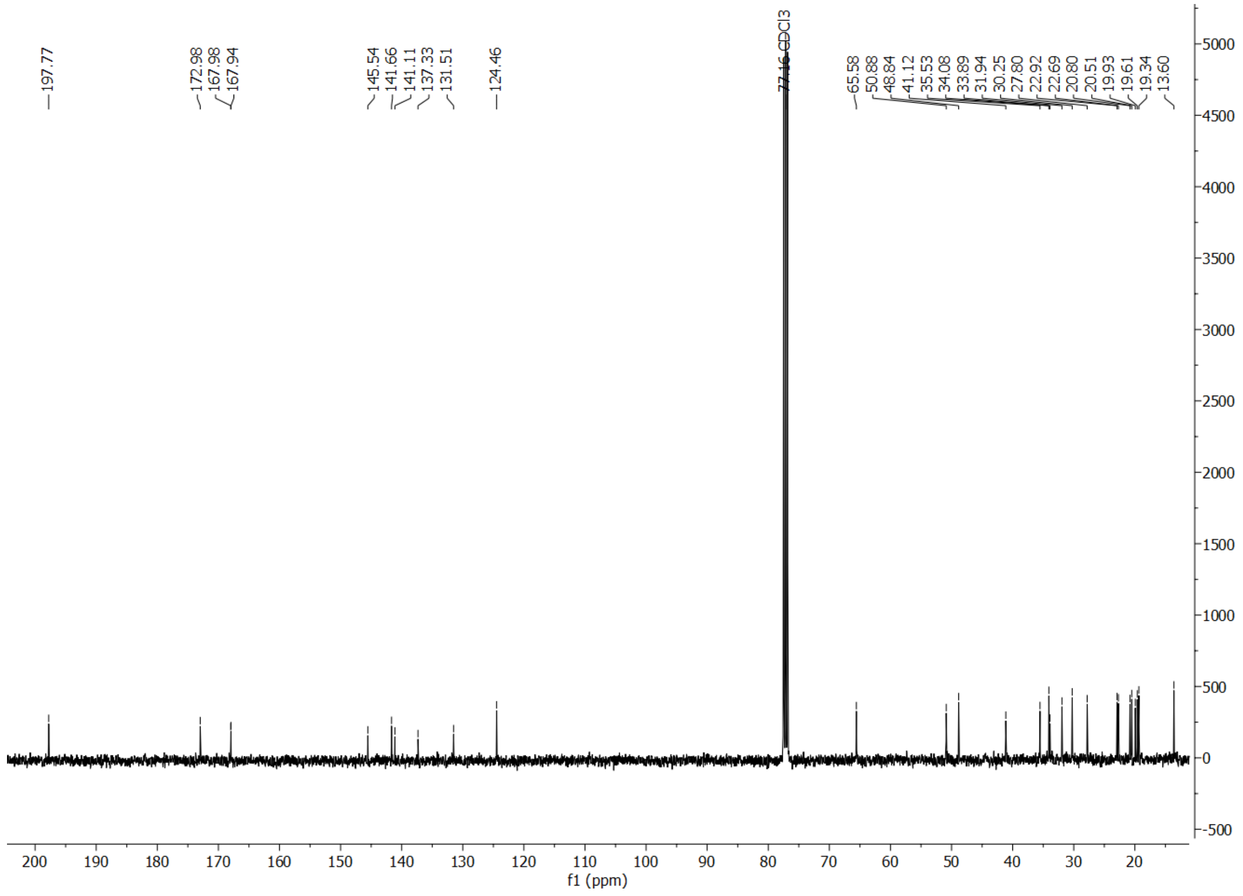

8. NMR data for compound 9

*8.1. ^{1}H spectrum for compound **9** recorded in $CDCl_3$*

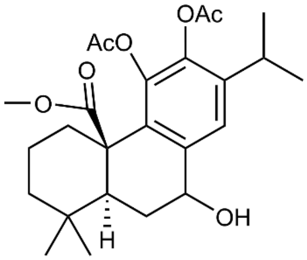

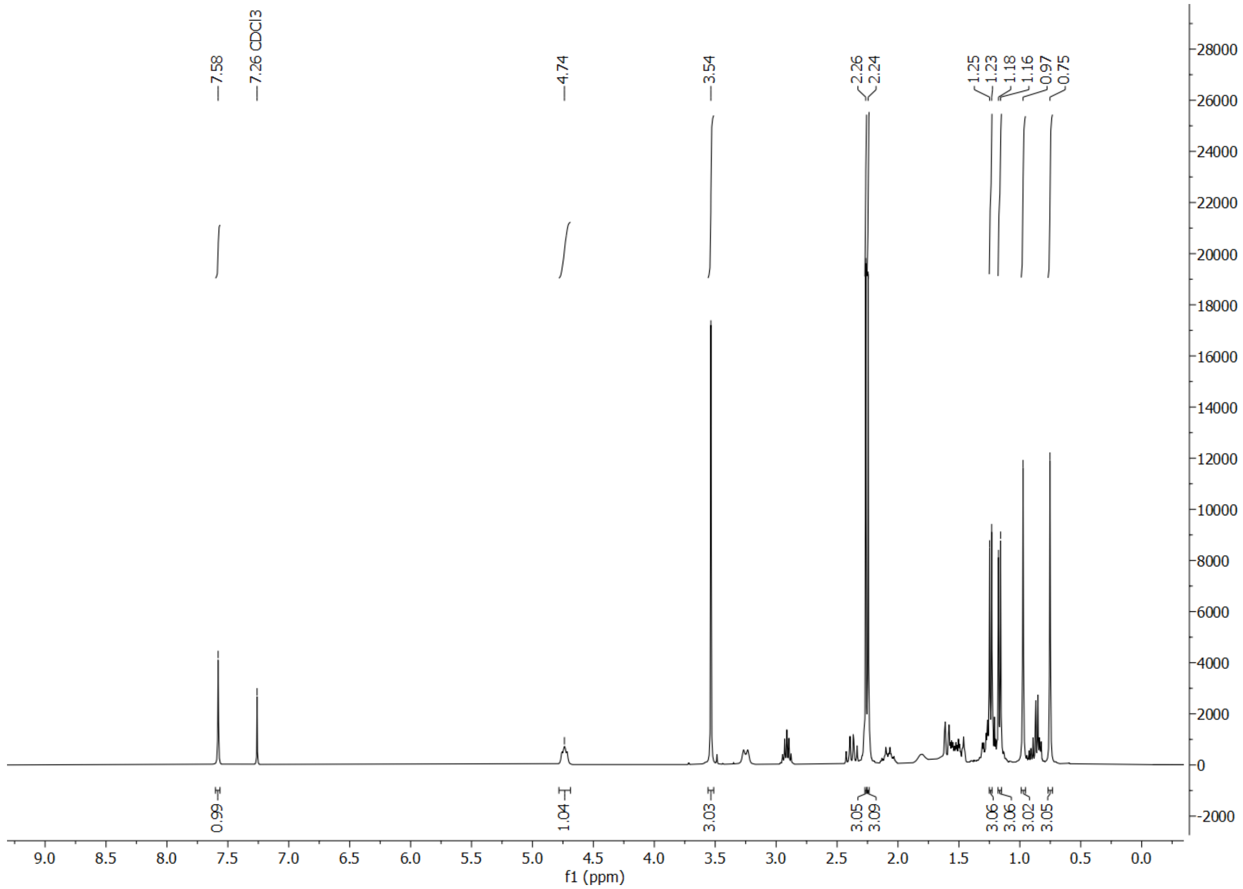

*8.2. ^{13}C spectrum for compound **9** recorded in $CDCl_3$*

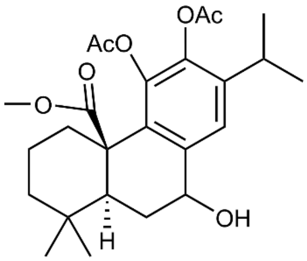

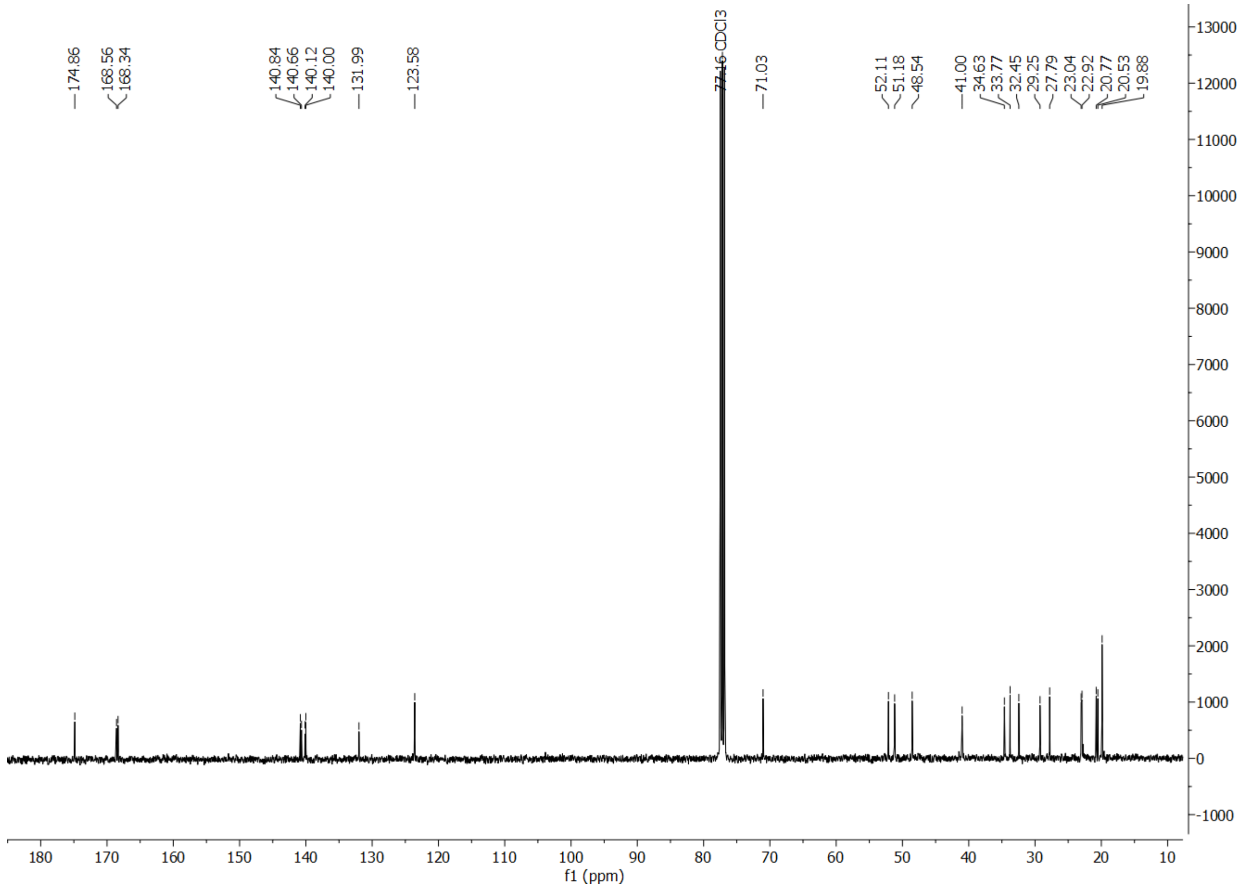

9. NMR data for compound 10

*9.1. ^{1}H spectrum for compound **10** recorded in $CDCl_3$*

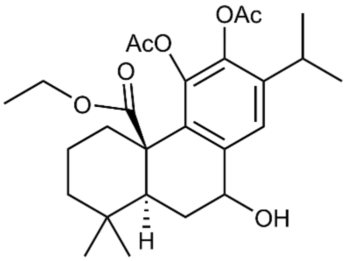

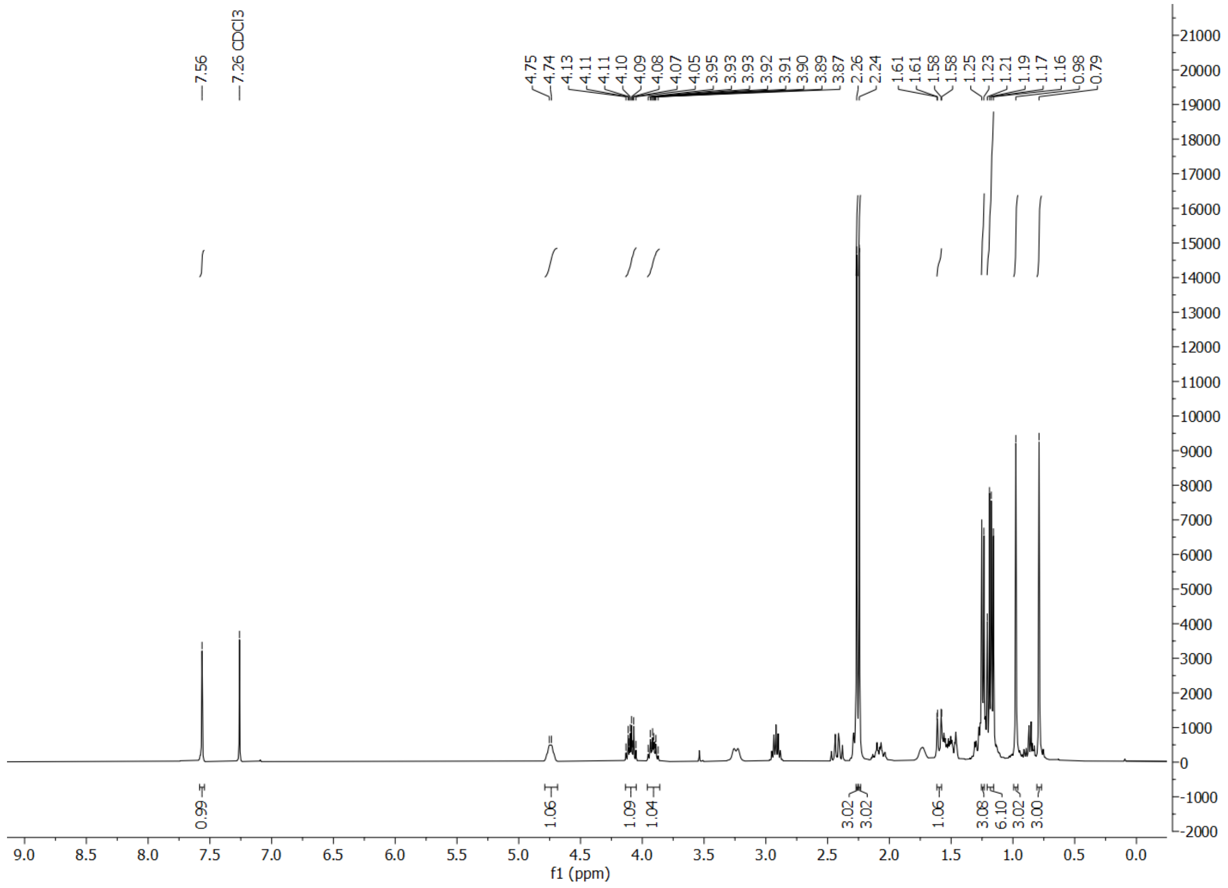

*9.2. ^{13}C spectrum for compound **10** recorded in $CDCl_3$*

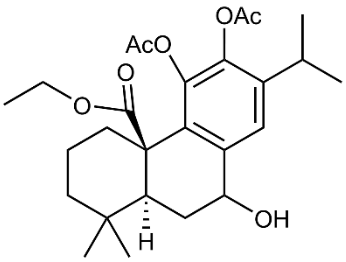

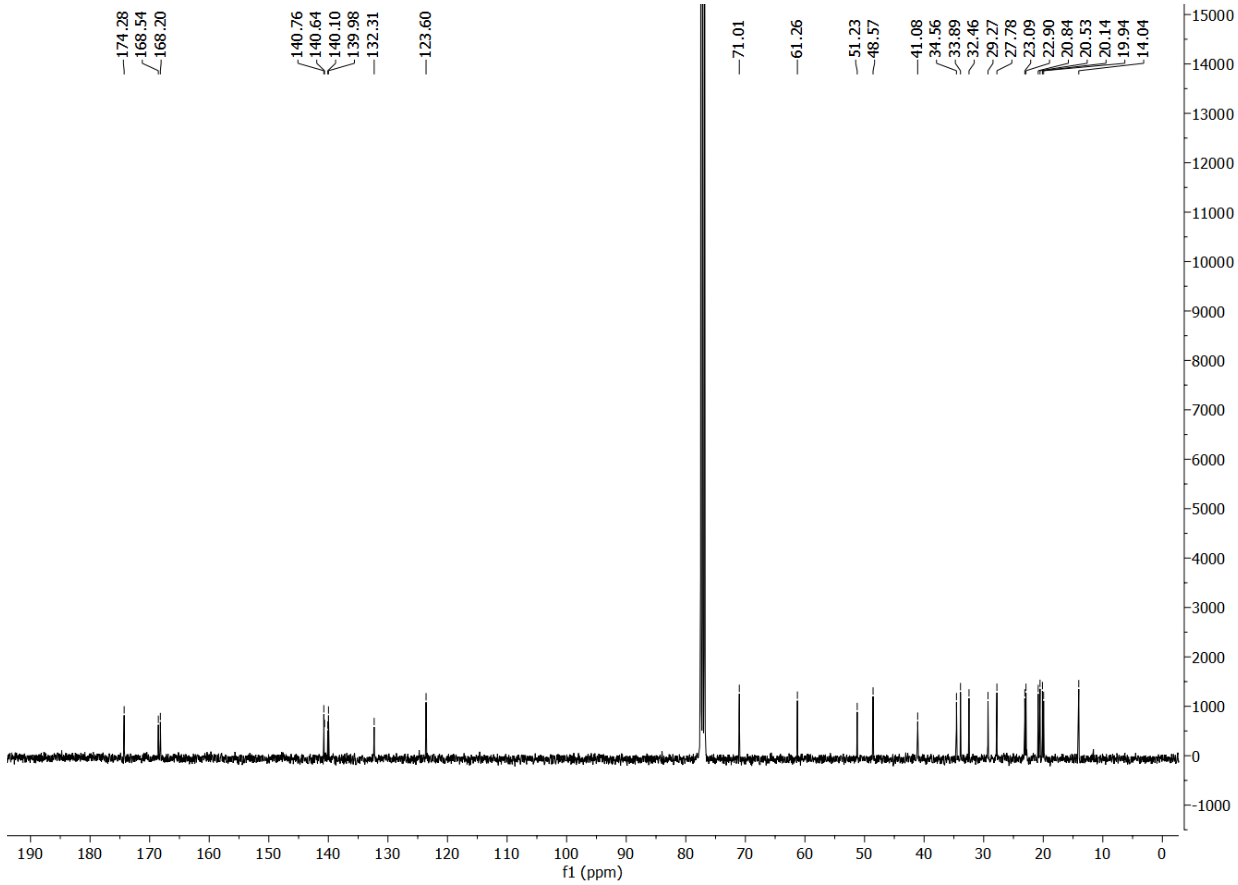

*9.3. NOESY spectrum for compound **10** recorded in $CDCl_3$*

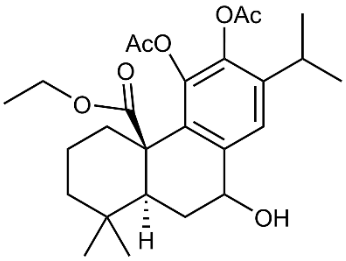

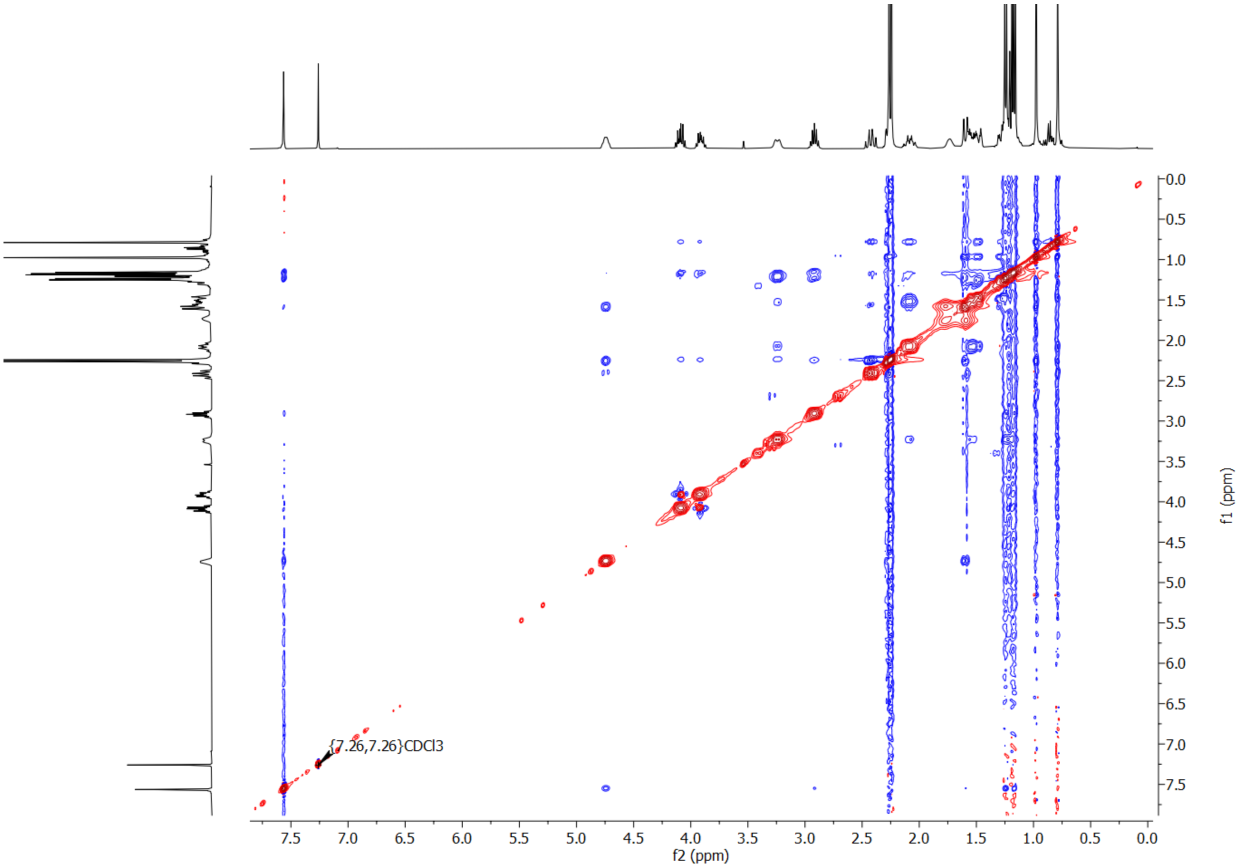

*9.4. HSQC spectrum for compound **10** recorded in $CDCl_3$*

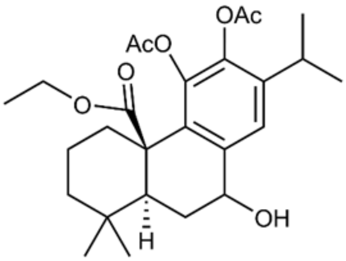

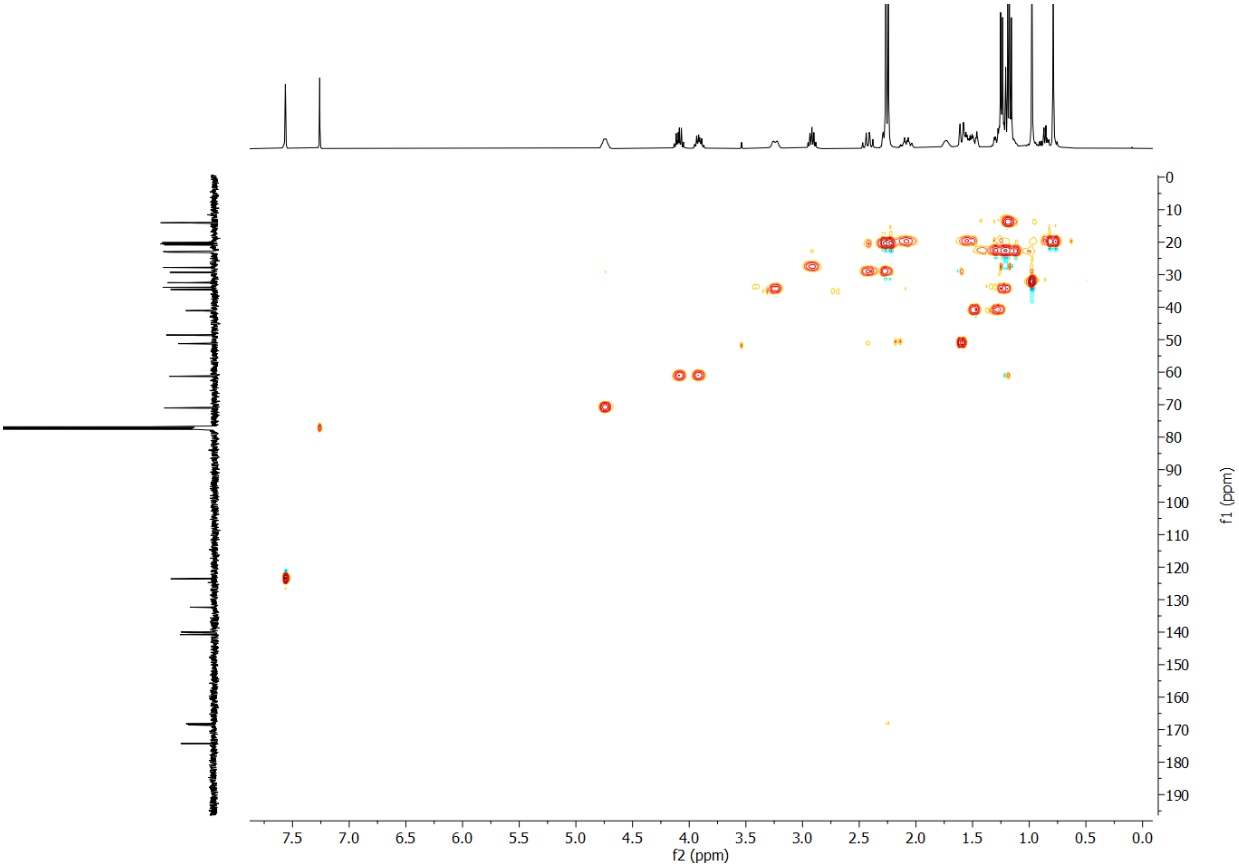

*9.5. HMBC spectrum for compound **10** recorded in $CDCl_3$*

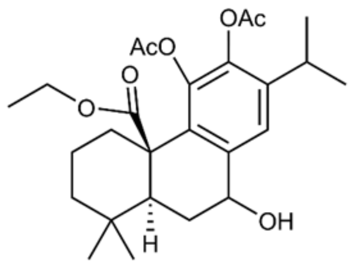

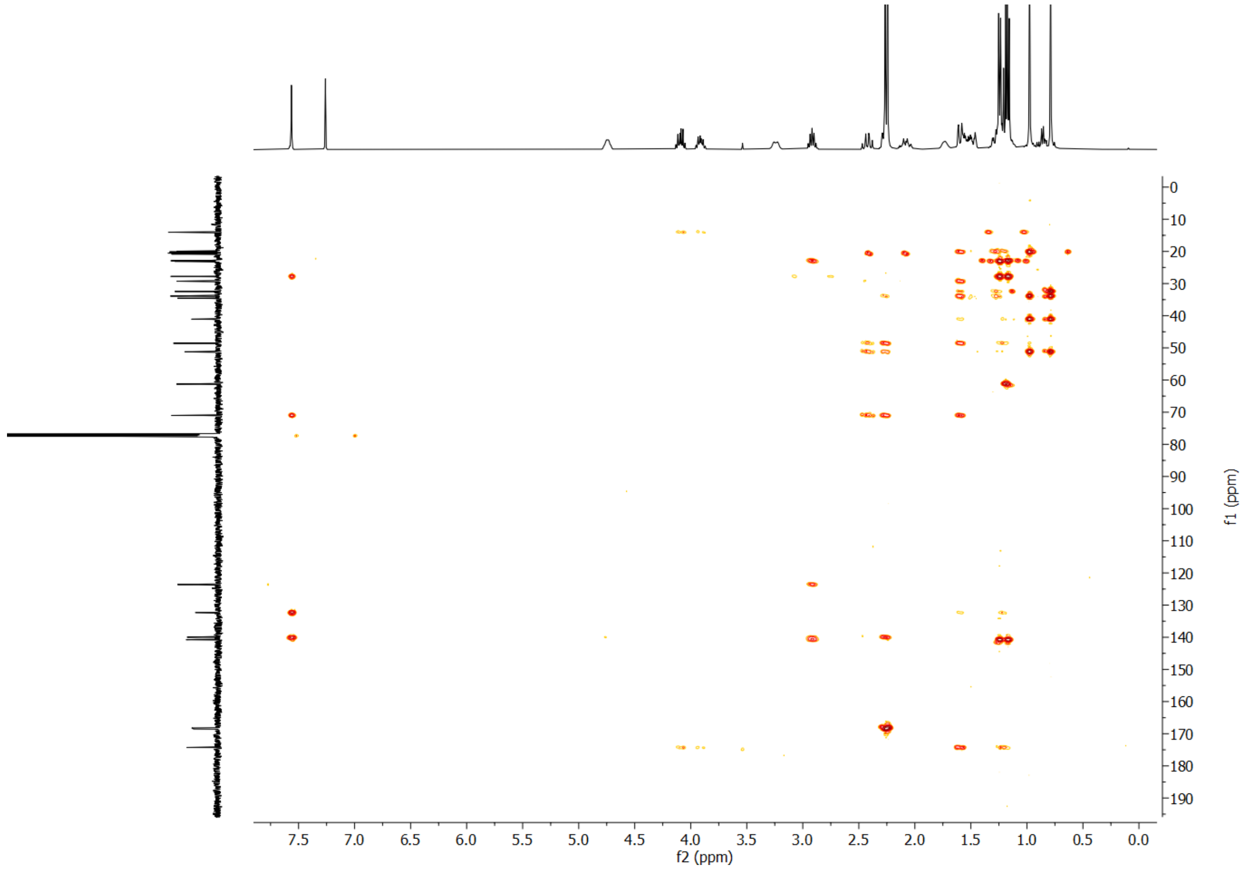

10. NMR data for compound 11

*10.1. ^{1}H spectrum for compound **11** recorded in $CDCl_3$*

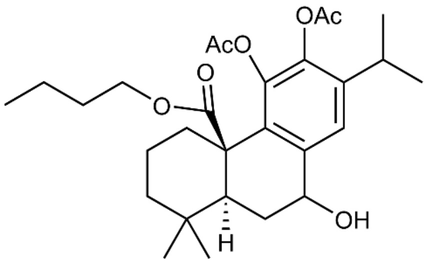

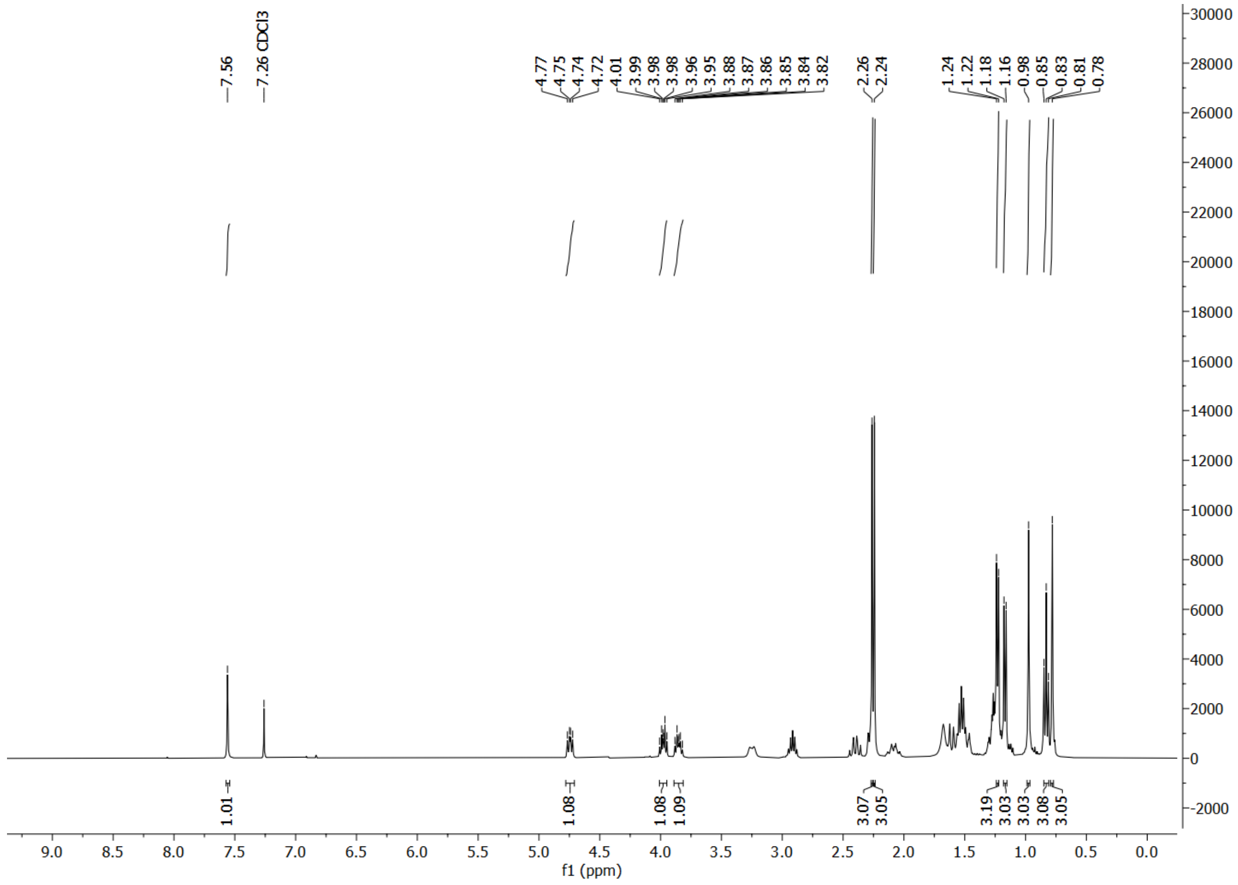

*10.2. ^{13}C spectrum for compound **11** recorded in $CDCl_3$*

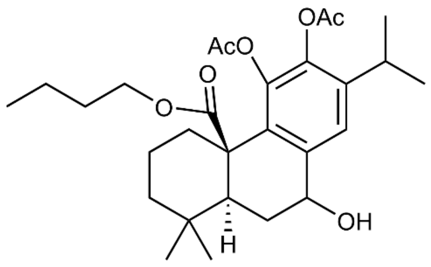

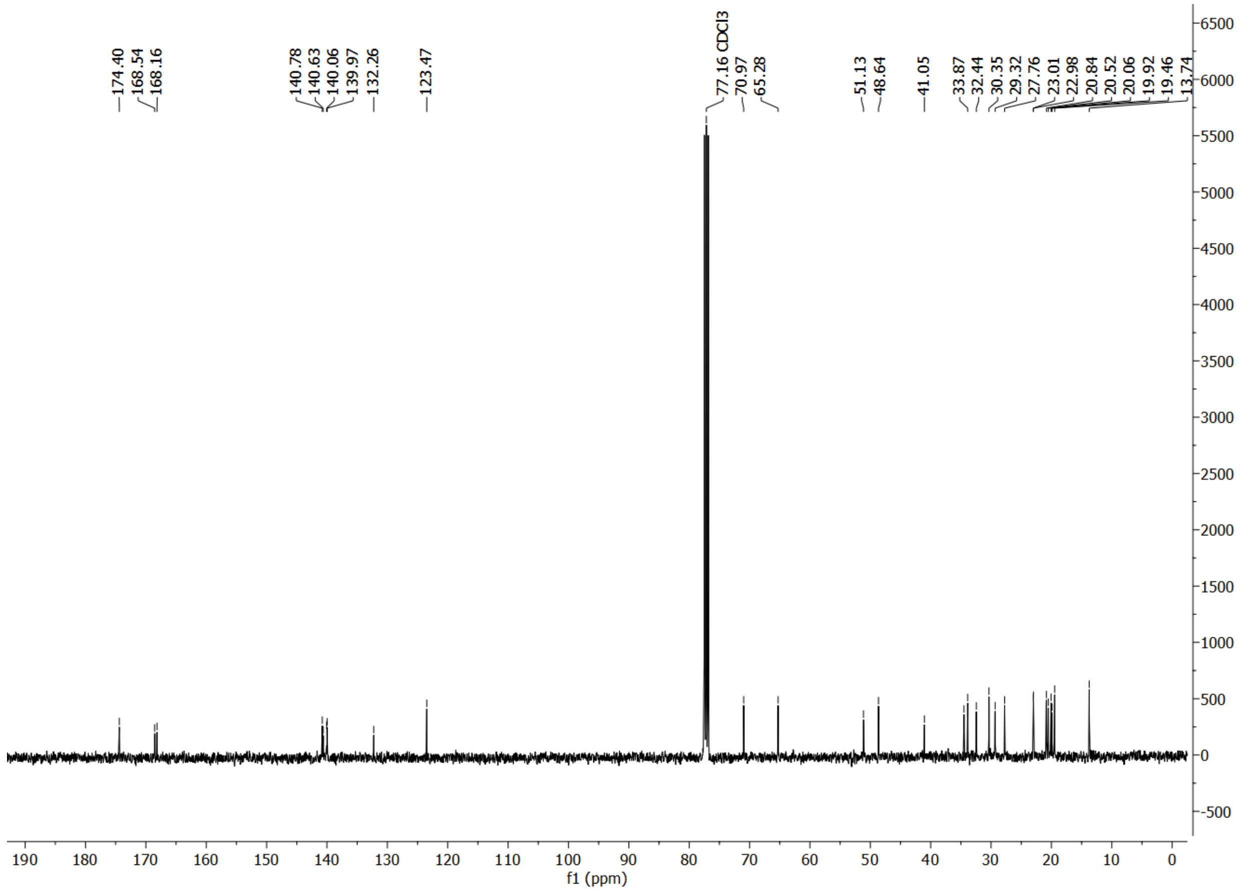

11. NMR data for compound 14

*11.1. ^{1}H spectrum for compound **14** recorded in $CDCl_3$*

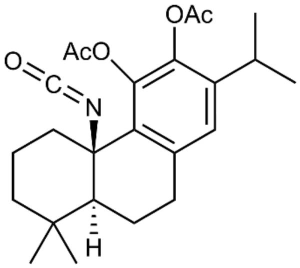

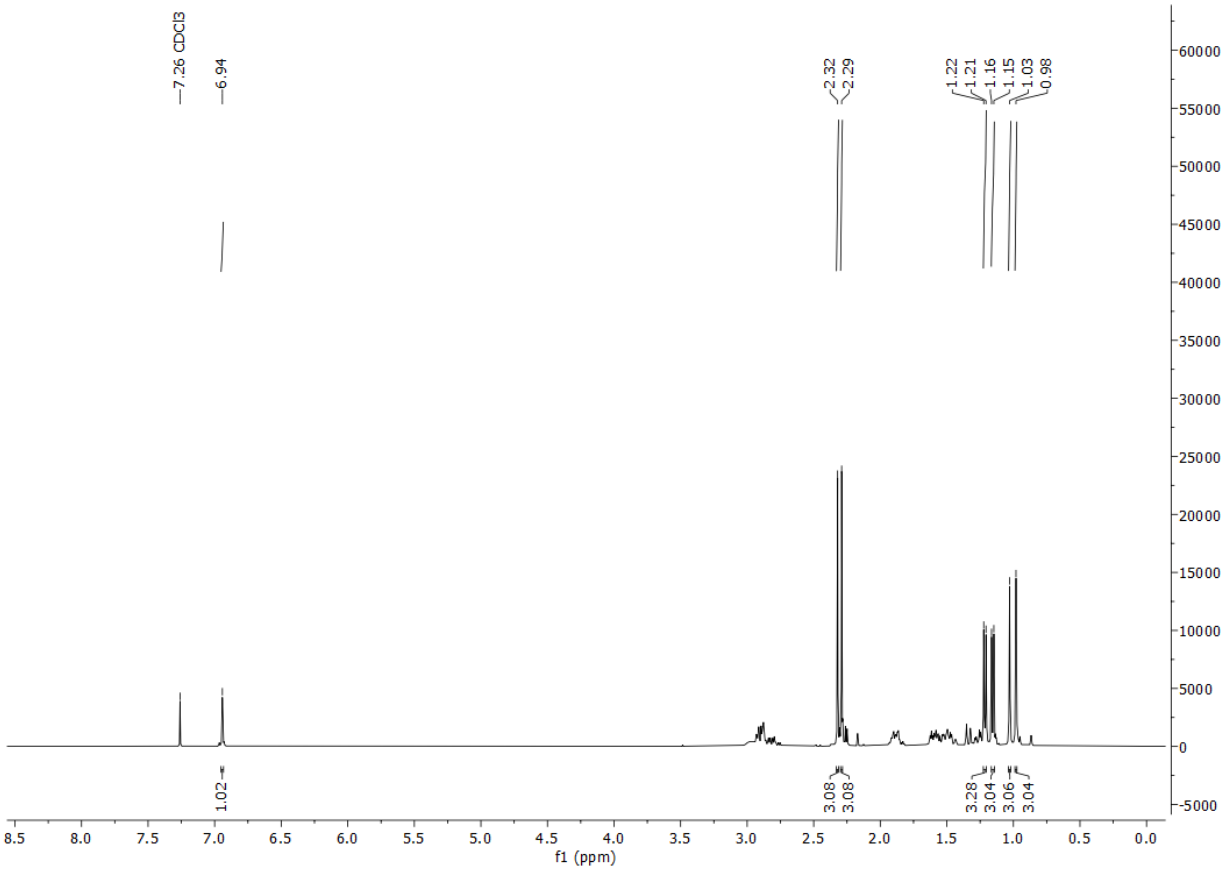

*11.2. ^{13}C spectrum for compound **14** recorded in $CDCl_3$*

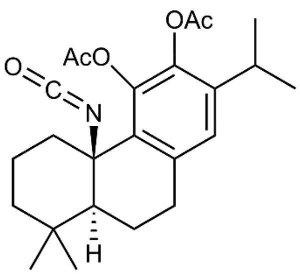

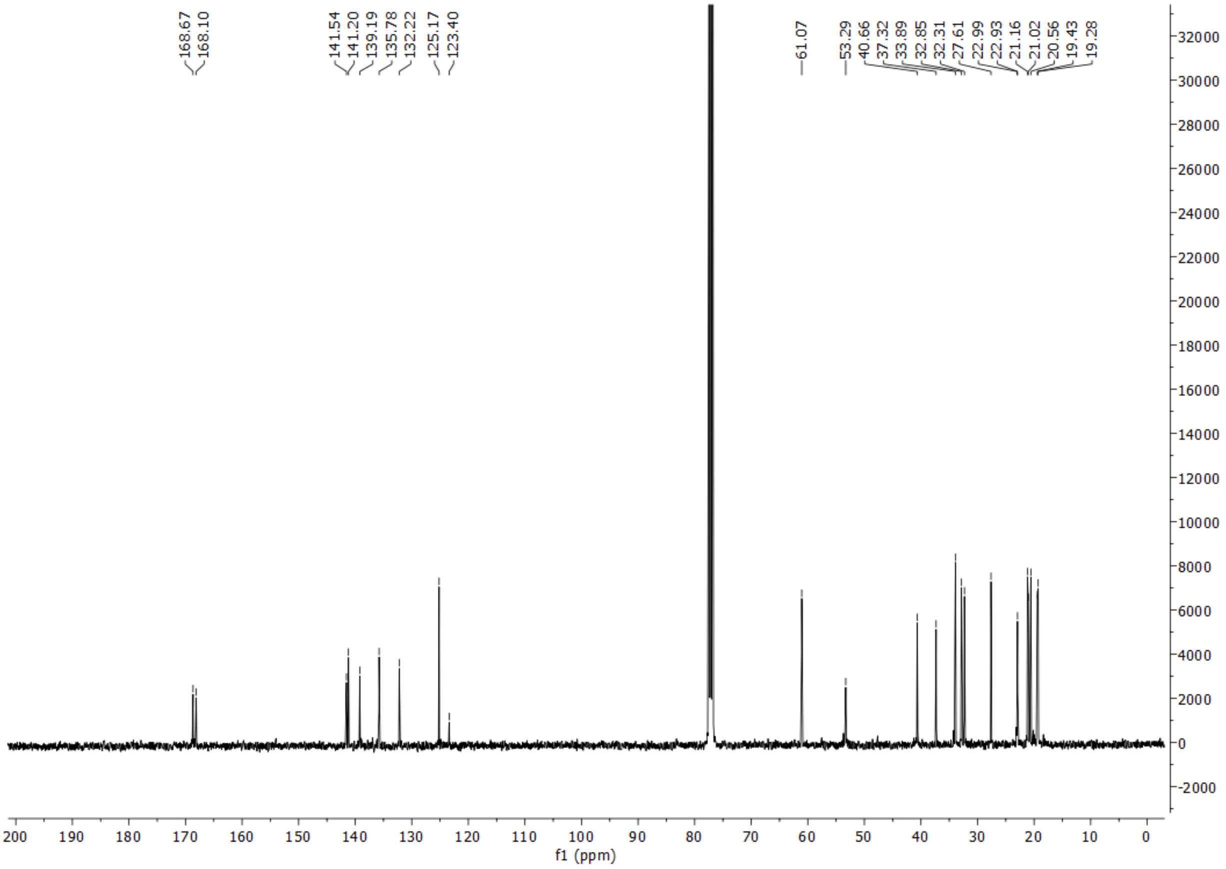

12. NMR data for compound 15

*12.1. ¹H spectrum for compound **15** recorded in $CDCl_3$*

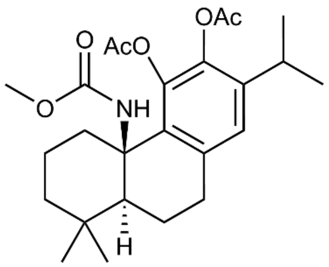

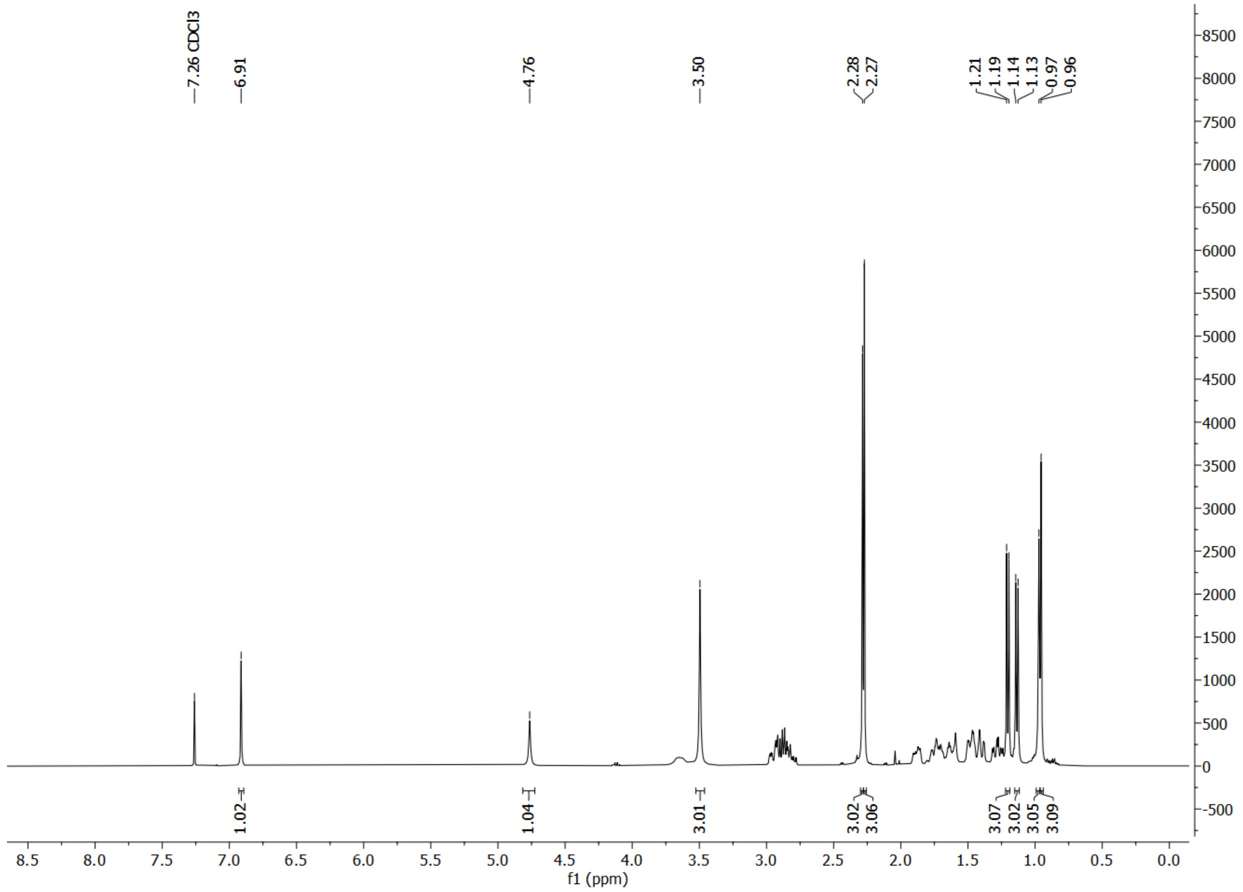

*12.2. ^{13}C spectrum for compound **15** recorded in $CDCl_3$*

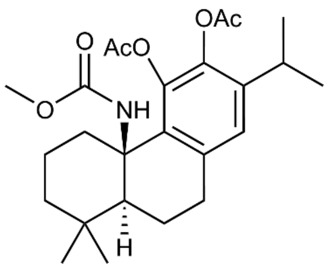

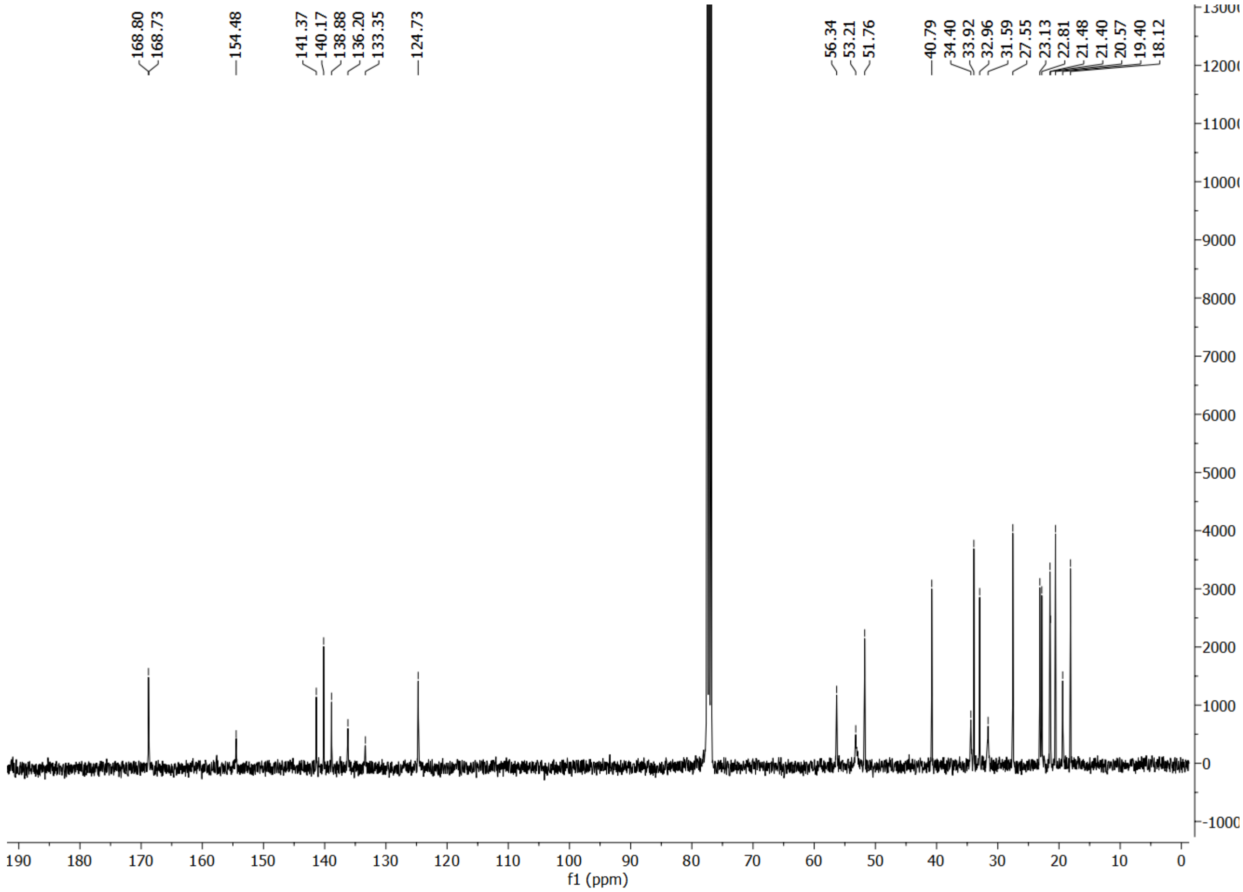

13. NMR data for compound 16

*13.1. ^{1}H spectrum for compound **16** recorded in $CDCl_3$*

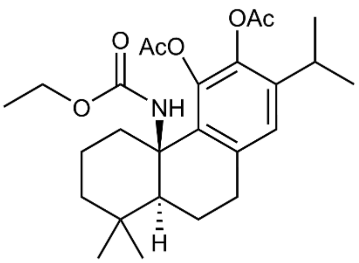

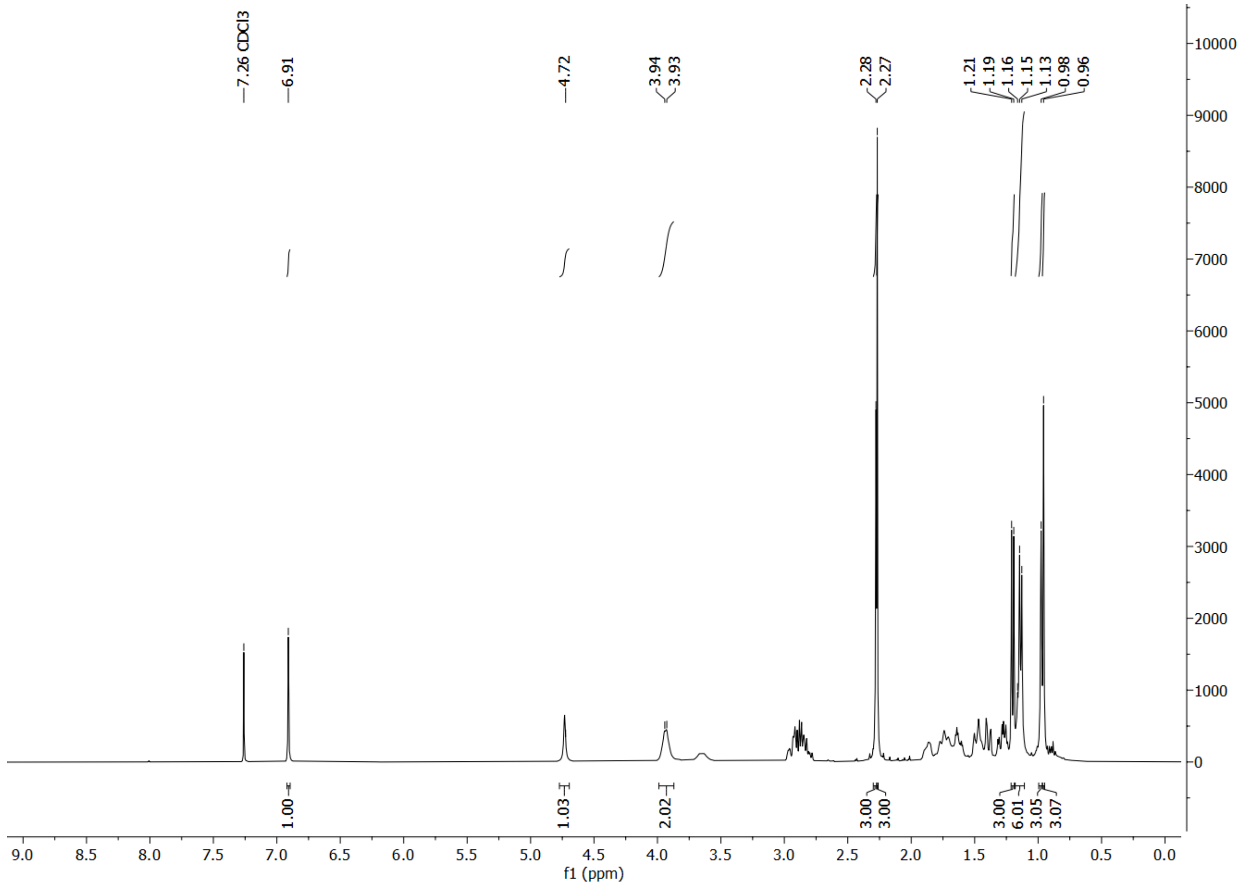

13.2. *^{13}C spectrum for compound **16** recorded in $CDCl_3$*

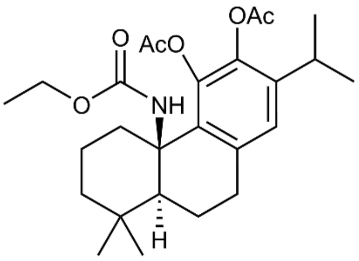

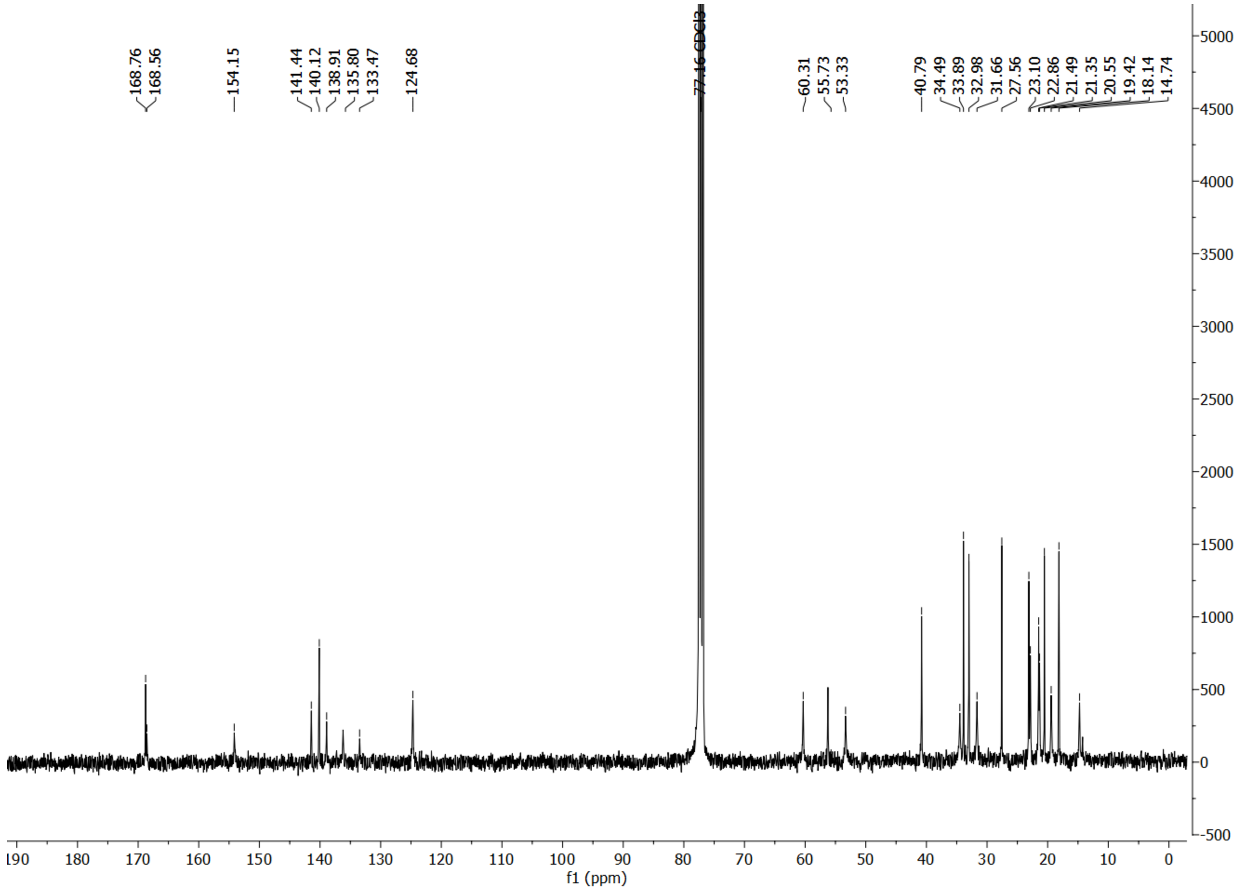

*13.3. COSY spectrum for compound **16** recorded in $CDCl_3$*

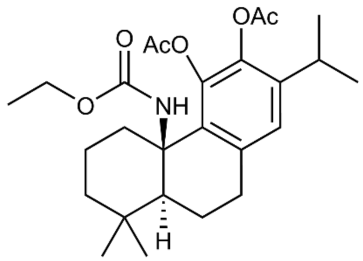

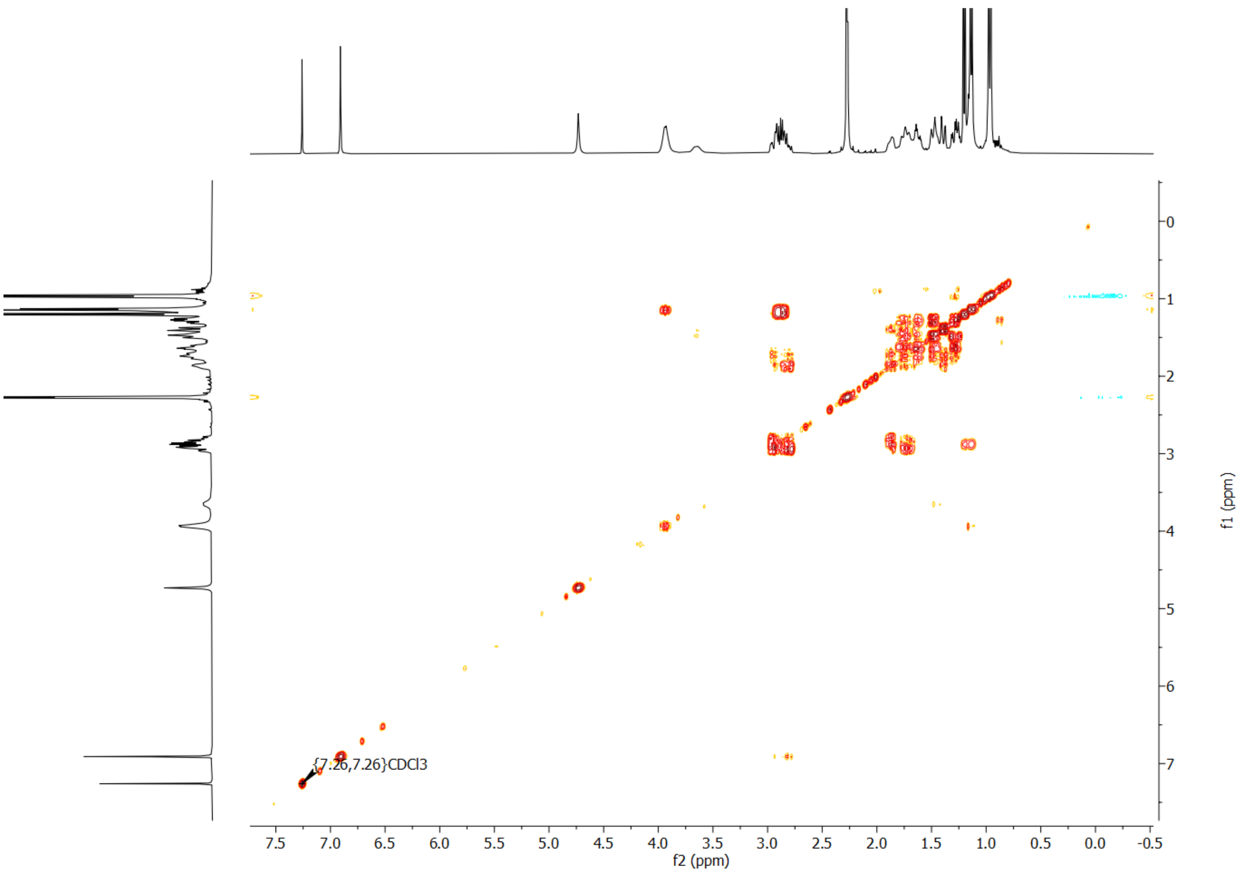

*13.4. HSQC spectrum for compound **16** recorded in $CDCl_3$*

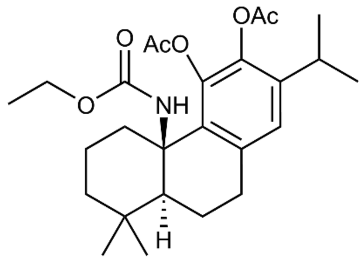

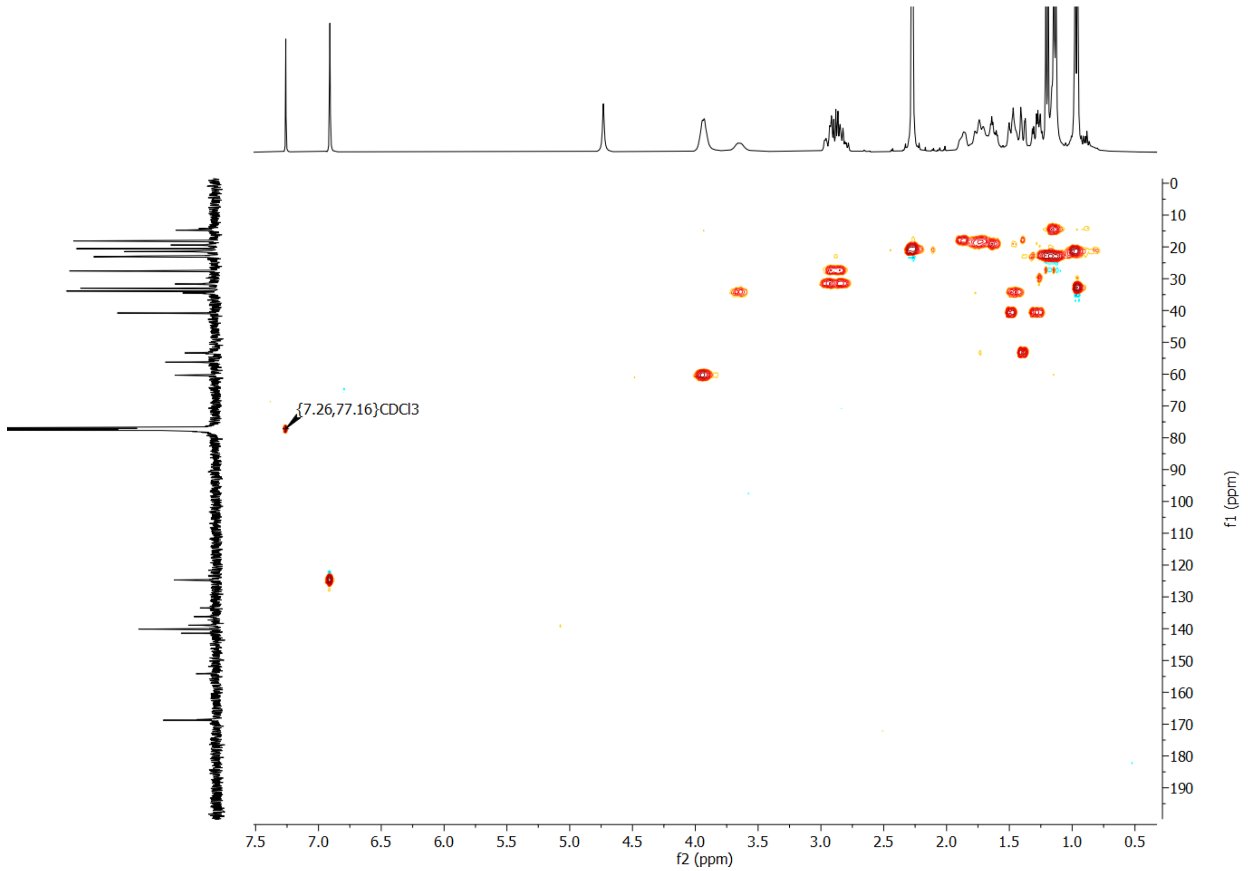

*13.5. HMBC spectrum for compound **16** recorded in $CDCl_3$*

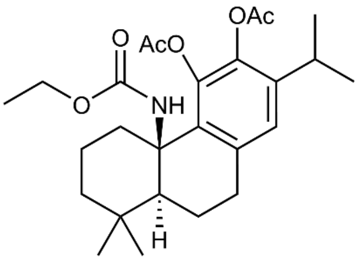

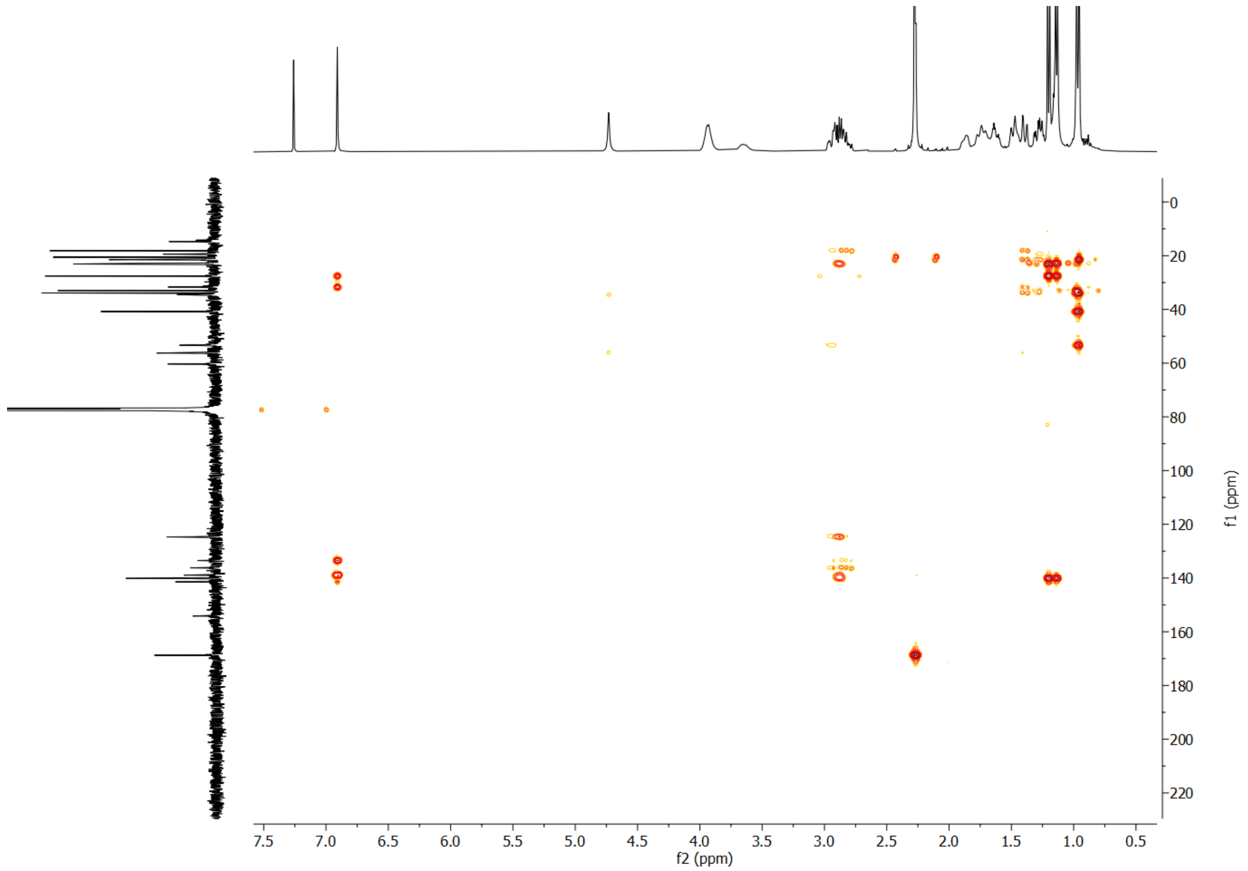

14. NMR data for compound 17

14.1. ^{1}H spectrum for compound ***17*** *recorded in CDCl₃*

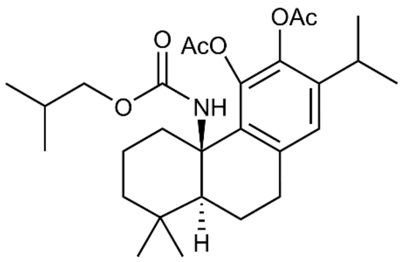

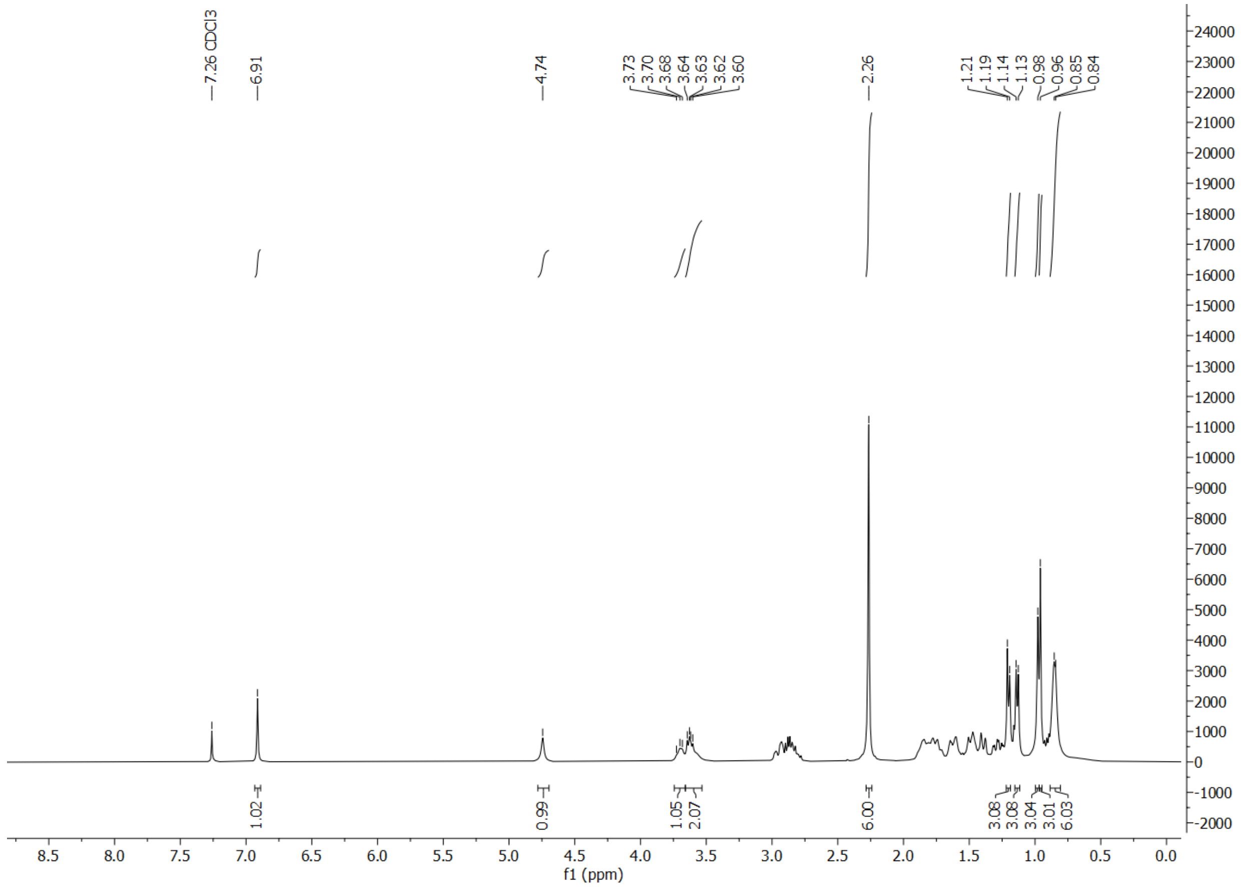

*14.2. ^{13}C spectrum for compound **17** recorded in $CDCl_3$*

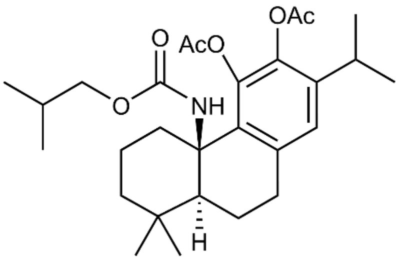

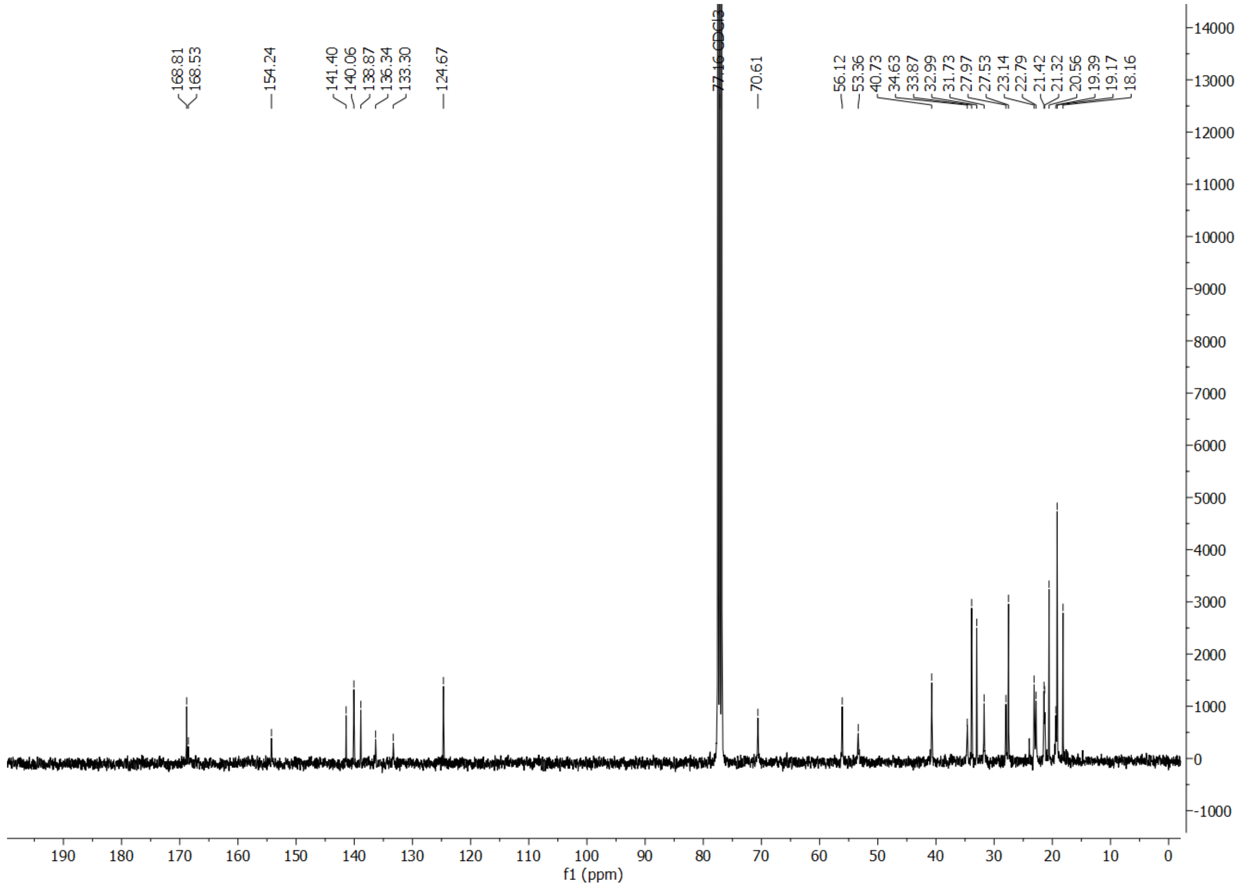

*14.3. COSY spectrum for compound **17** recorded in $CDCl_3$*

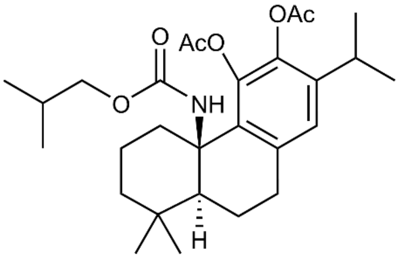

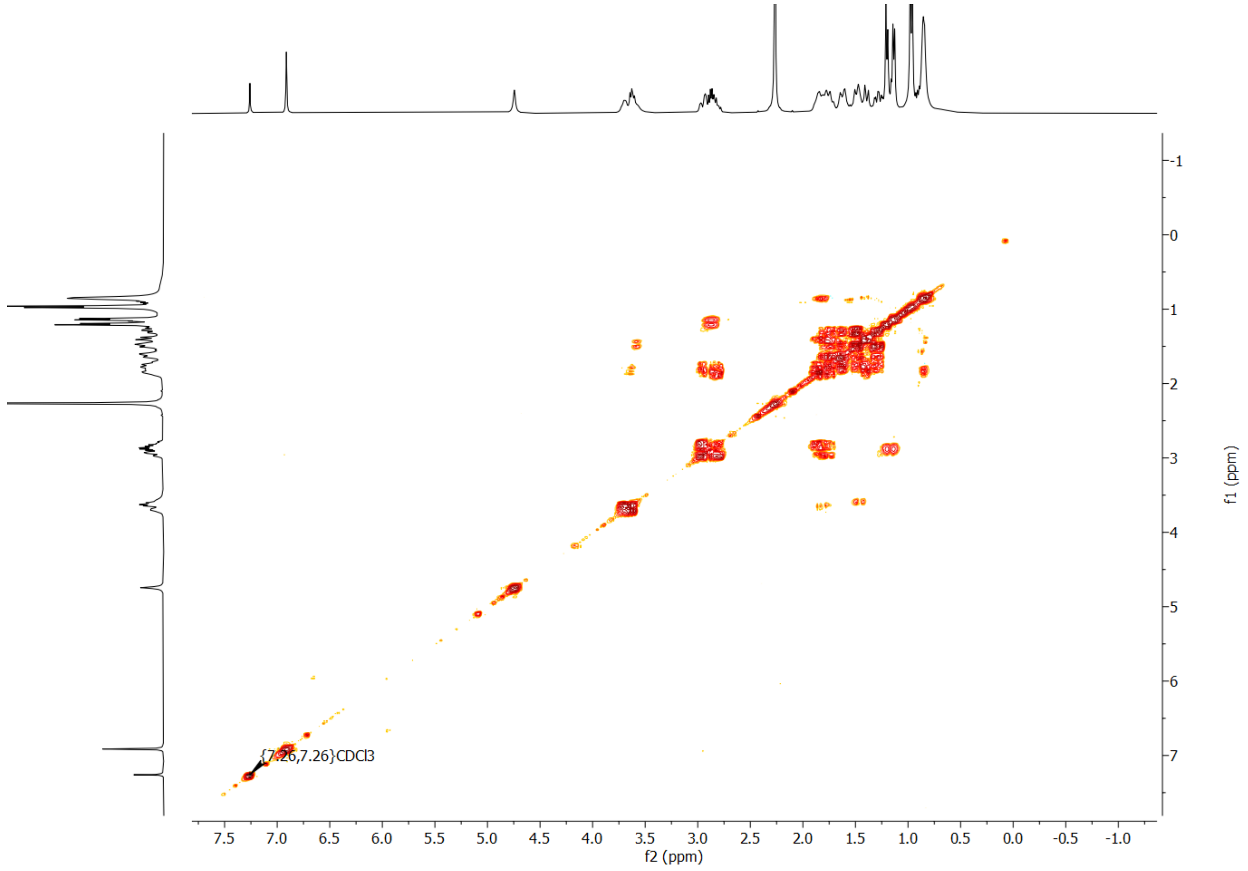

*14.4. HSQC spectrum for compound **17** recorded in $CDCl_3$*

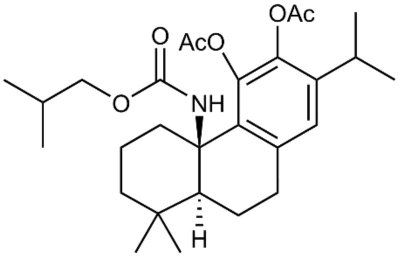

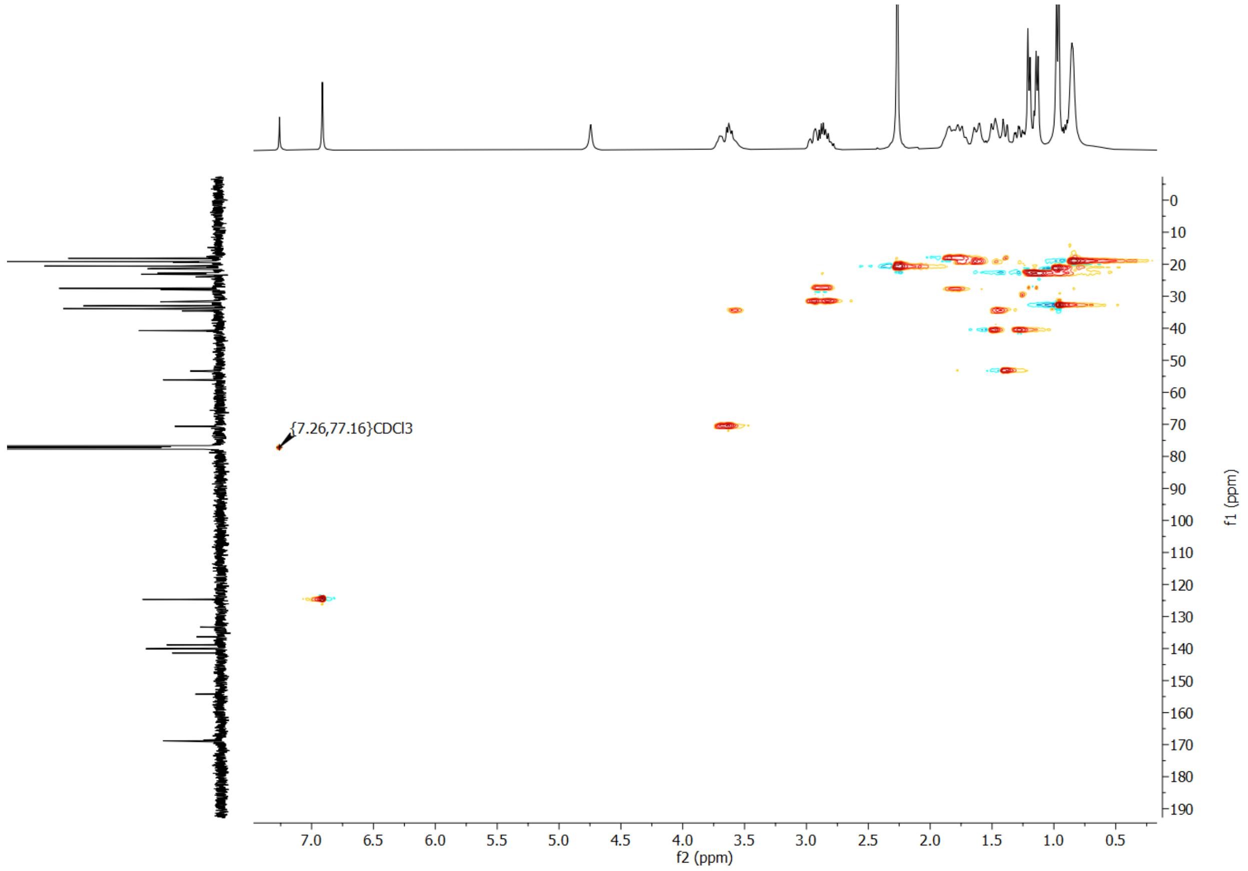

*14.5. HMBC spectrum for compound **17** recorded in $CDCl_3$*

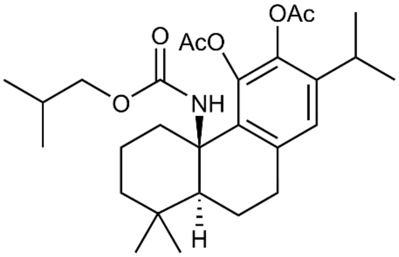

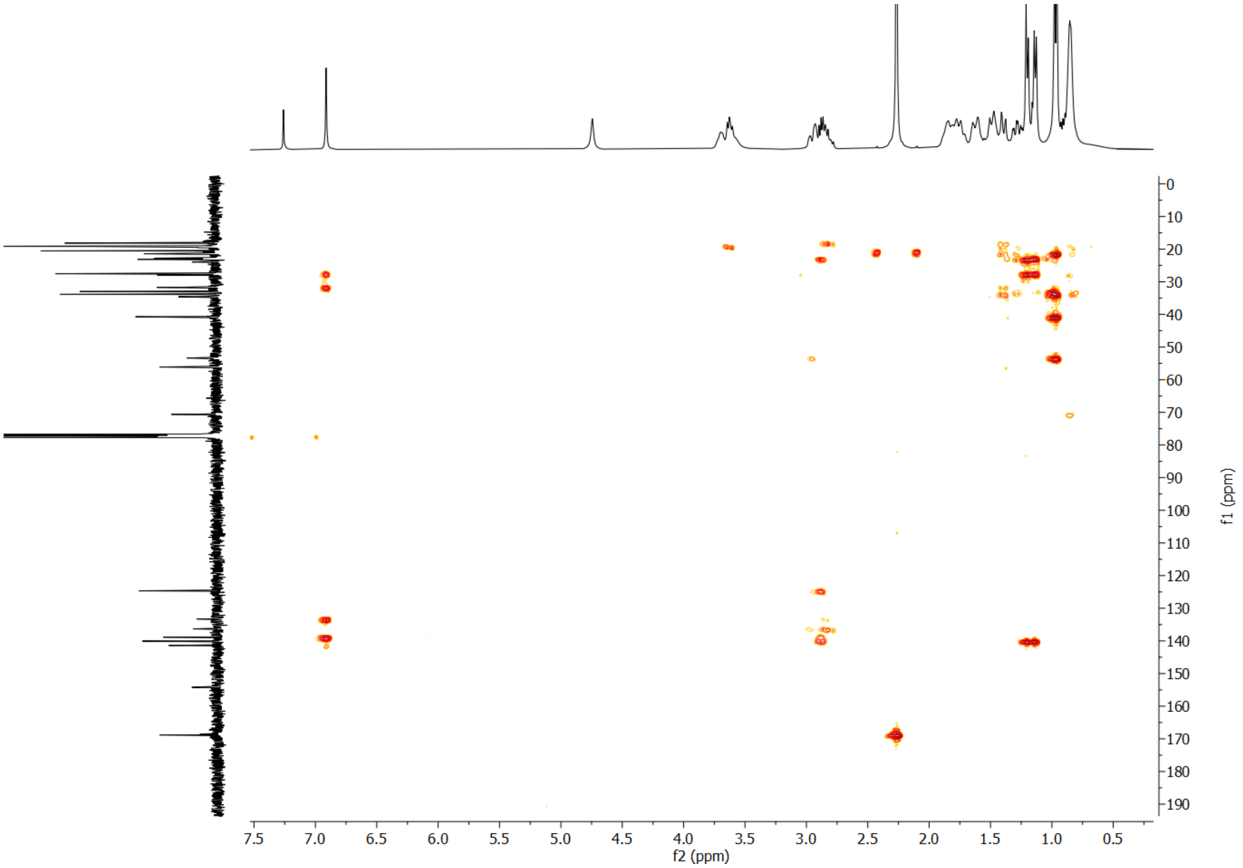

15. NMR data for compound 18

*15.1. ^{1}H spectrum for compound **18** recorded in CDCl₃*

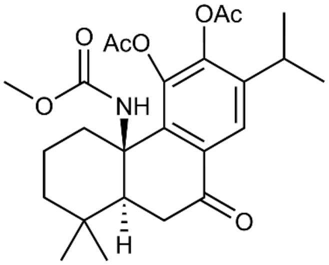

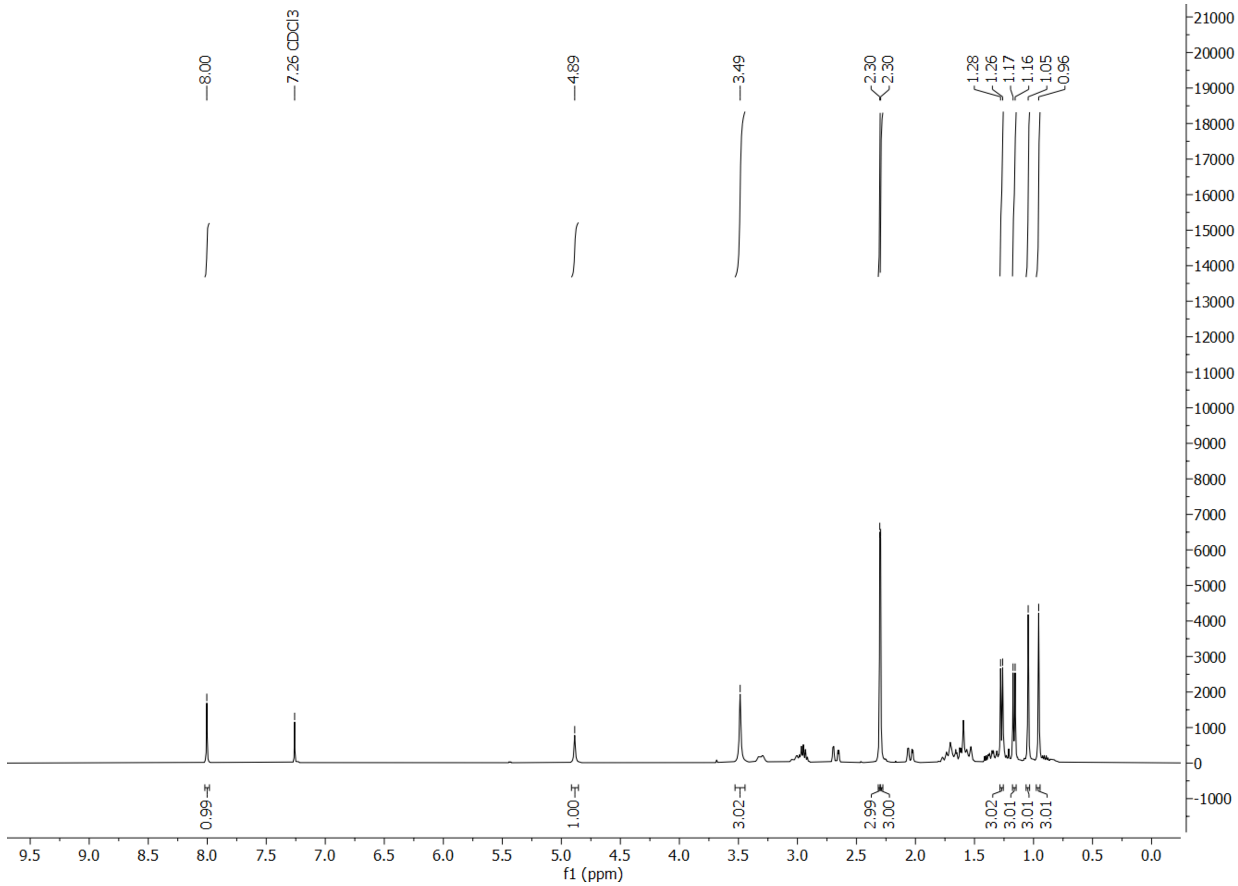

*15.2. ^{13}C spectrum for compound **18** recorded in CDCl$_3$*

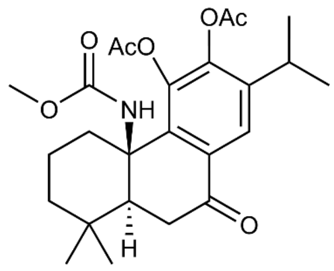

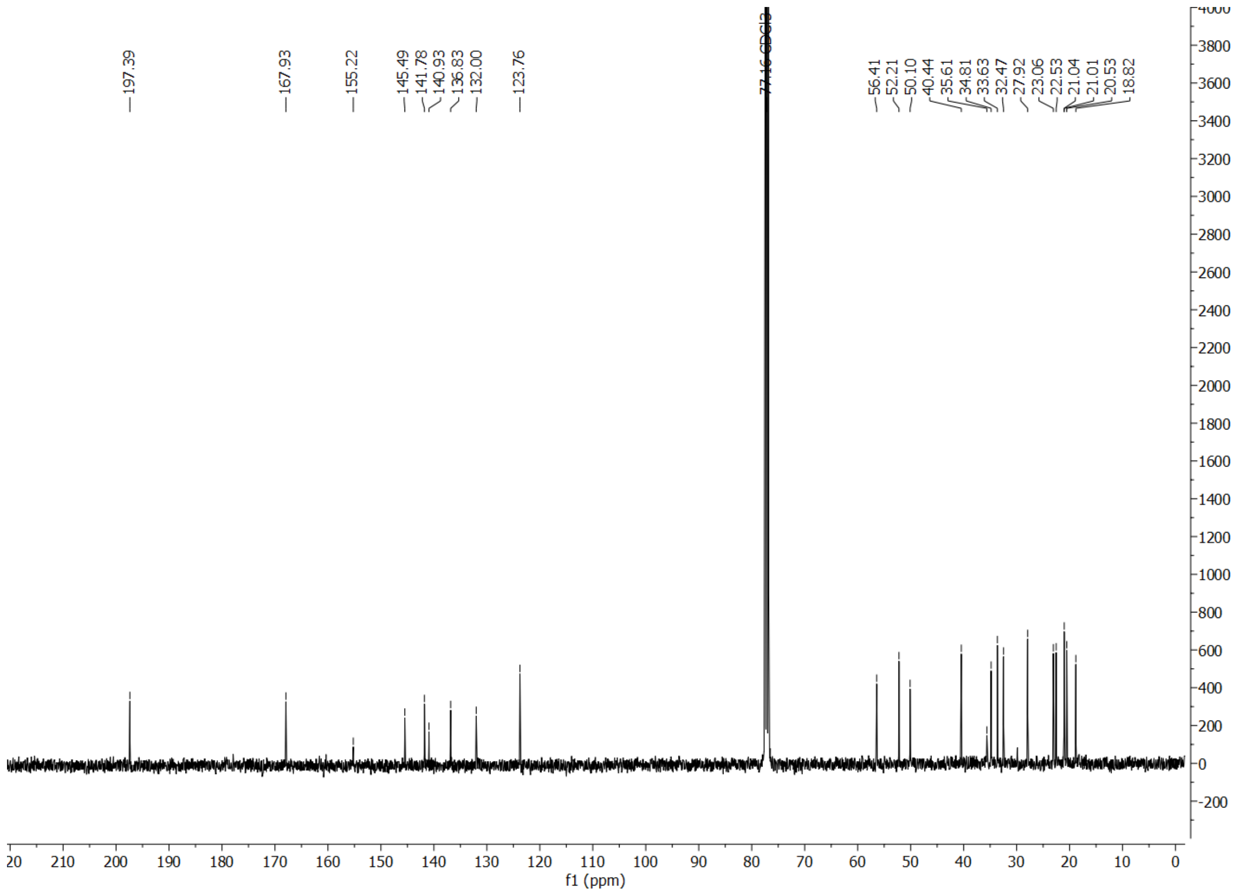

16. NMR data for compound 19

*16.1. ^{1}H spectrum for compound **19** recorded in $CDCl_3$*

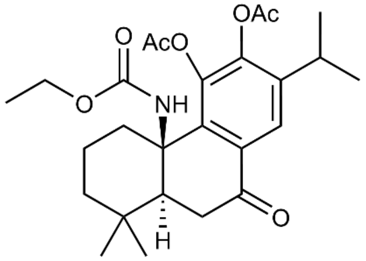

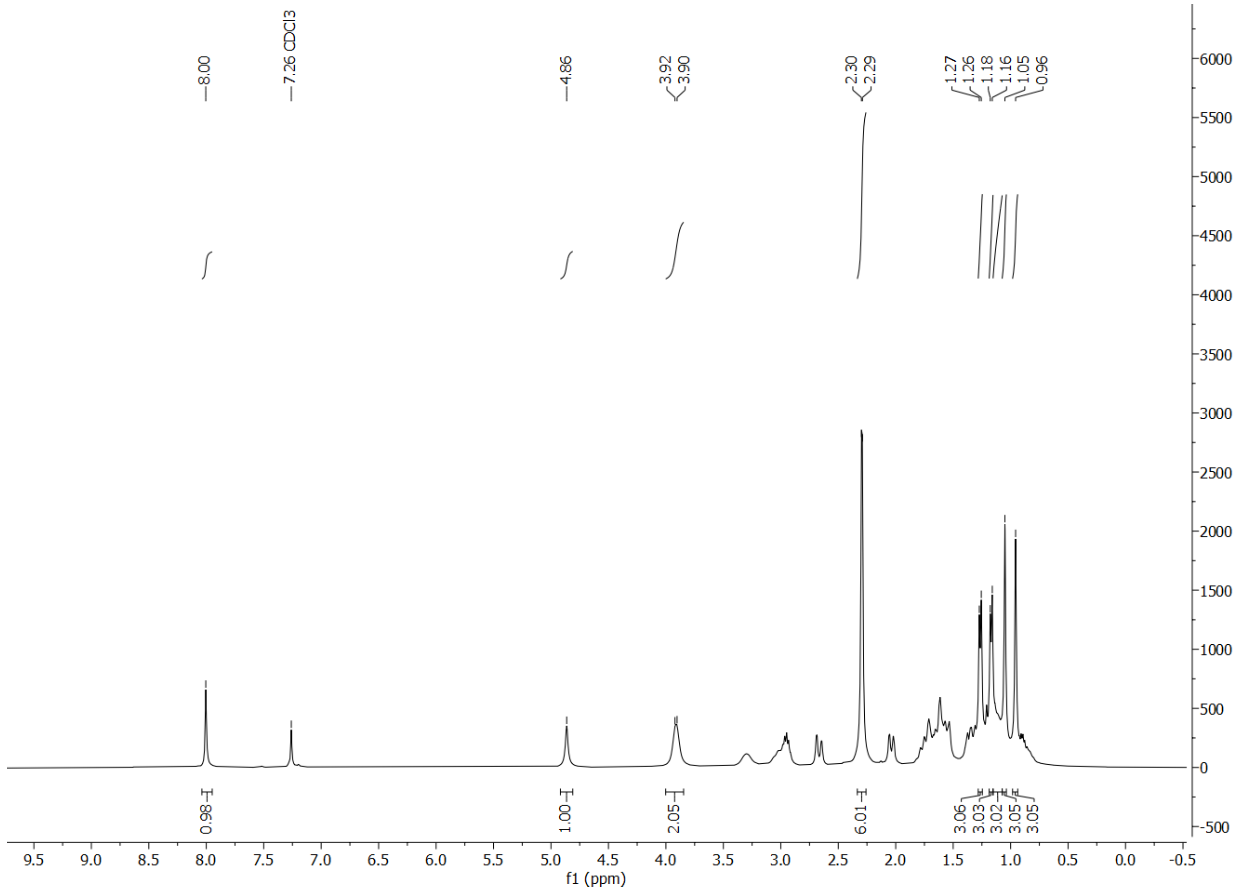

*16.2. ^{13}C spectrum for compound **19** recorded in $CDCl_3$*

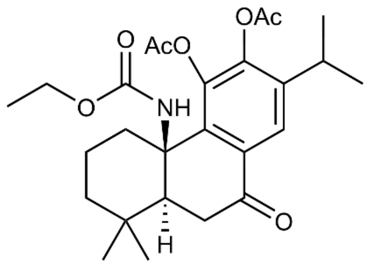

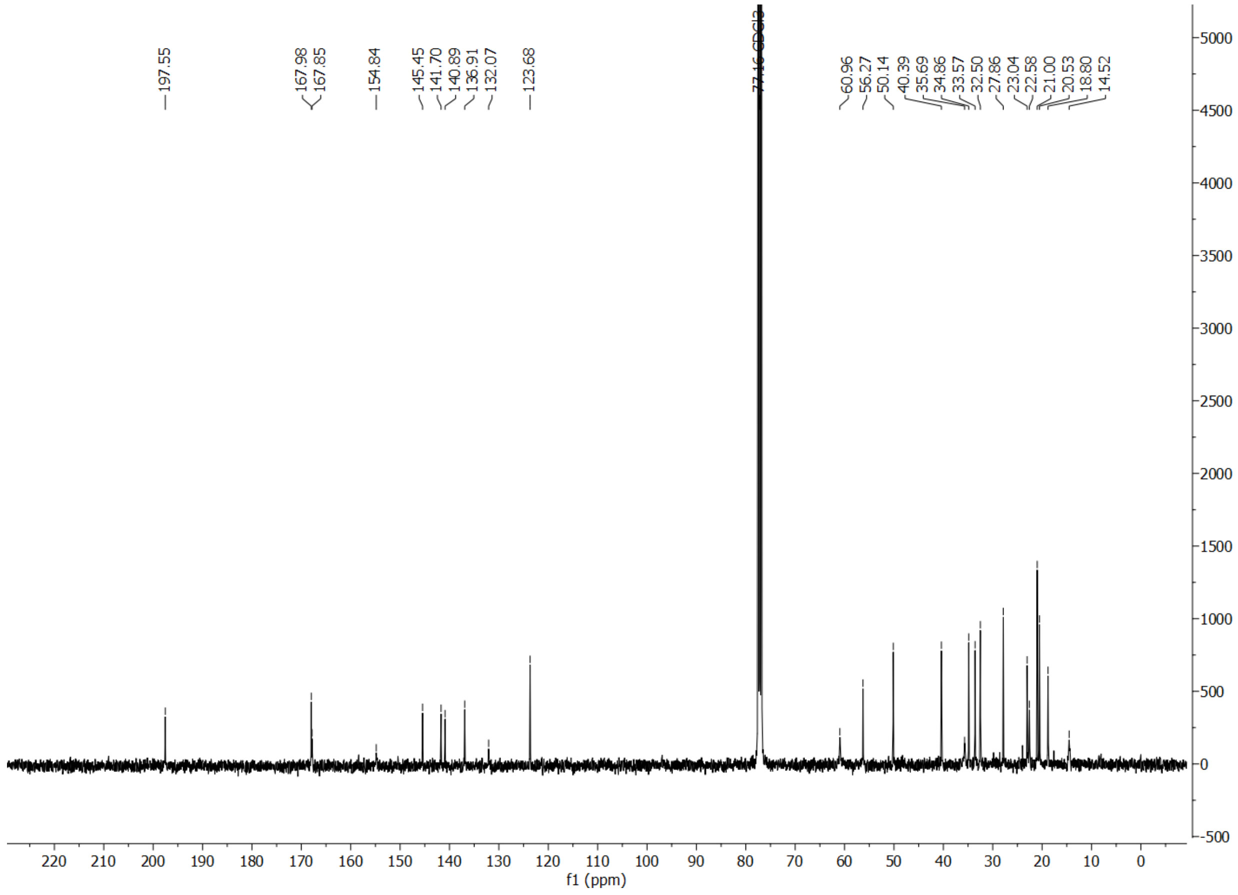

16.3. COSY spectrum for compound ***19*** *recorded in* $CDCl_3$

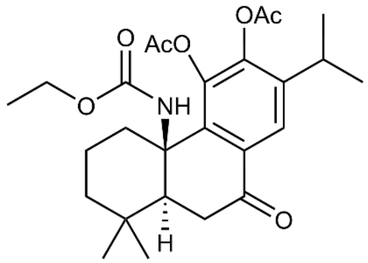

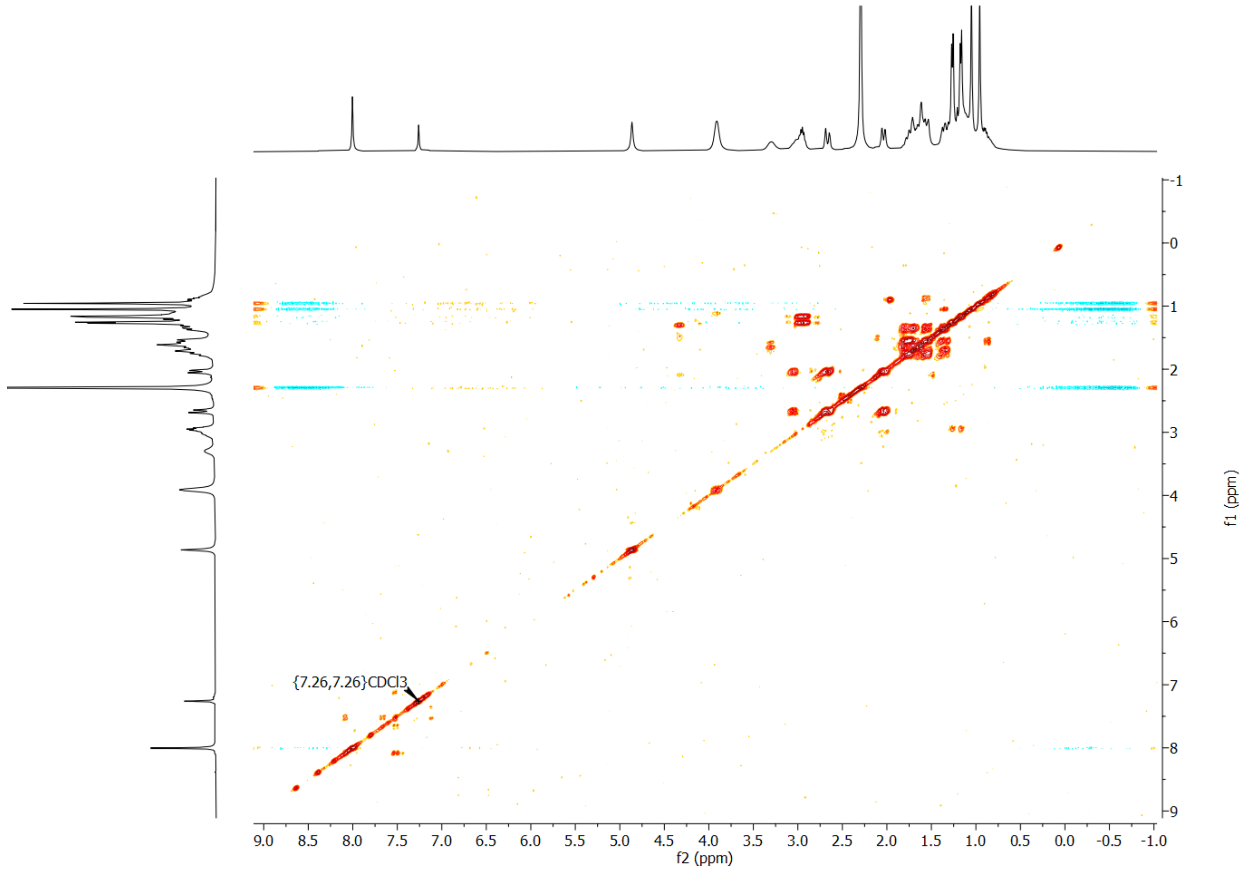

*16.4. NOESY spectrum for compound **19** recorded in $CDCl_3$*

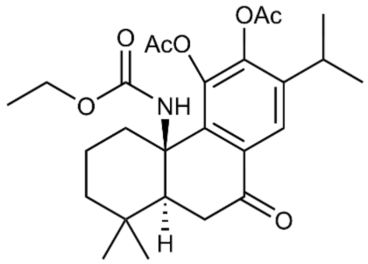

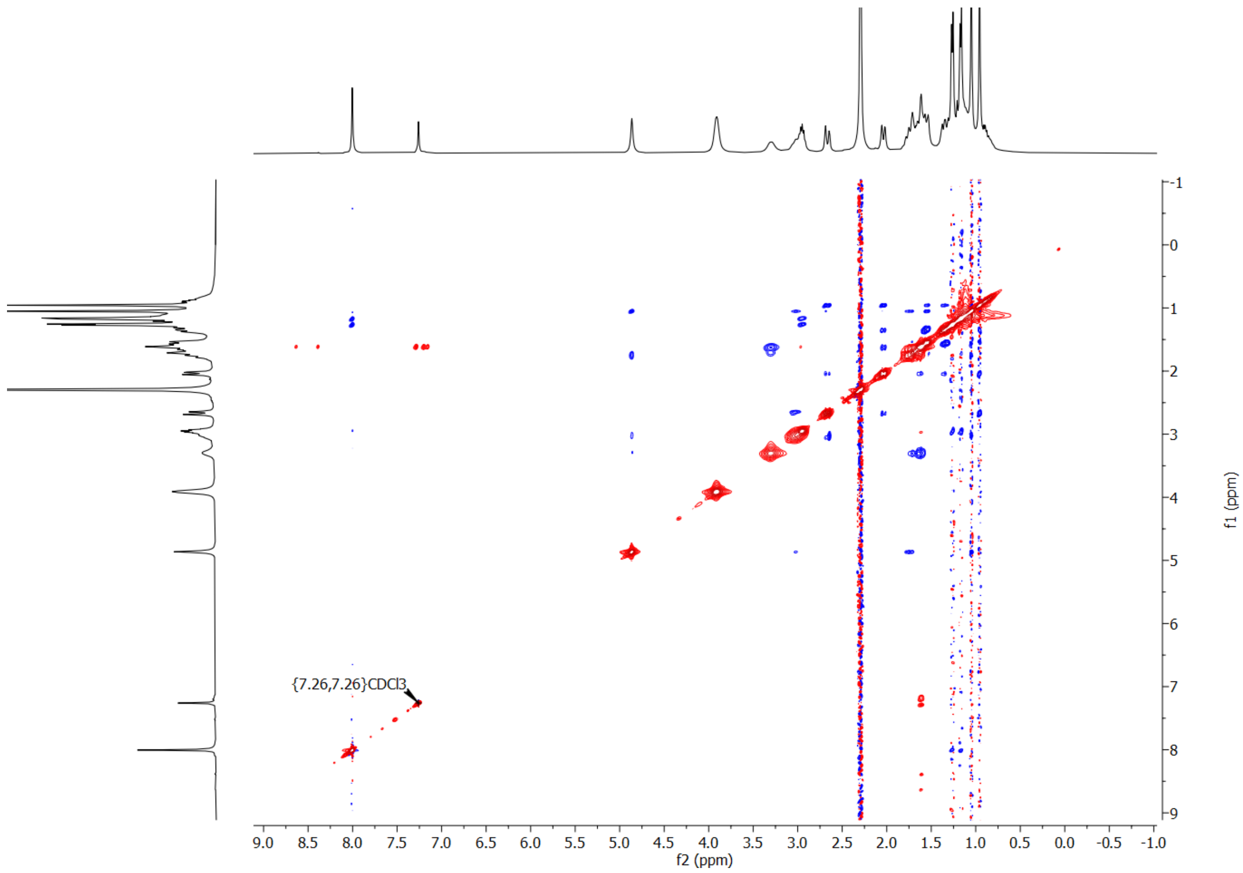

*16.5. HSQC spectrum for compound **19** recorded in $CDCl_3$*

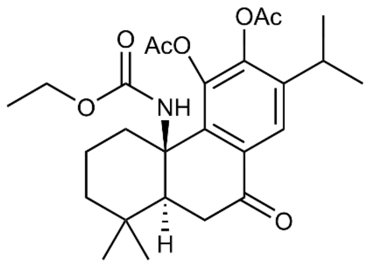

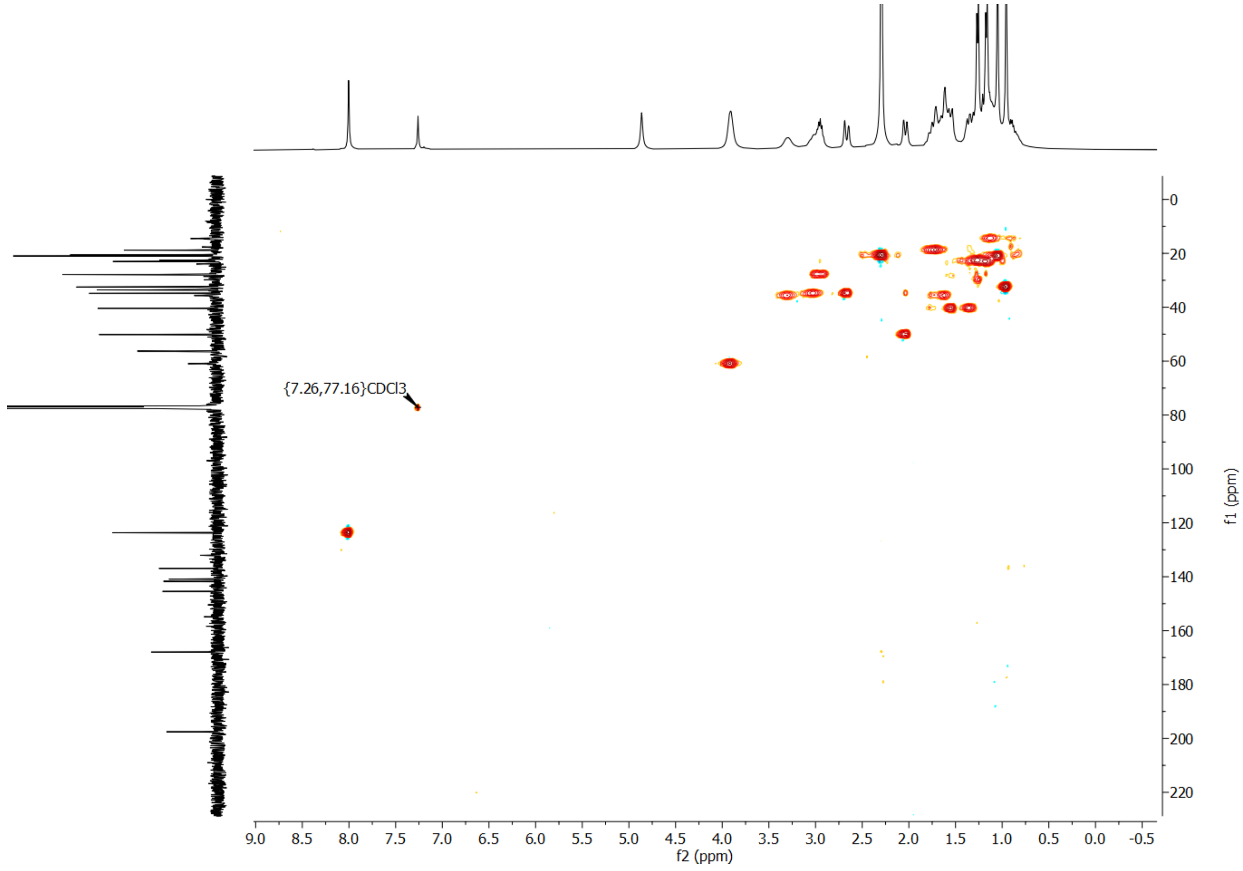

*16.6. HMBC spectrum for compound **19** recorded in $CDCl_3$*

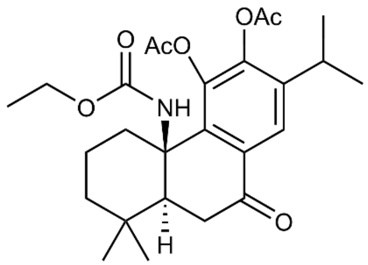

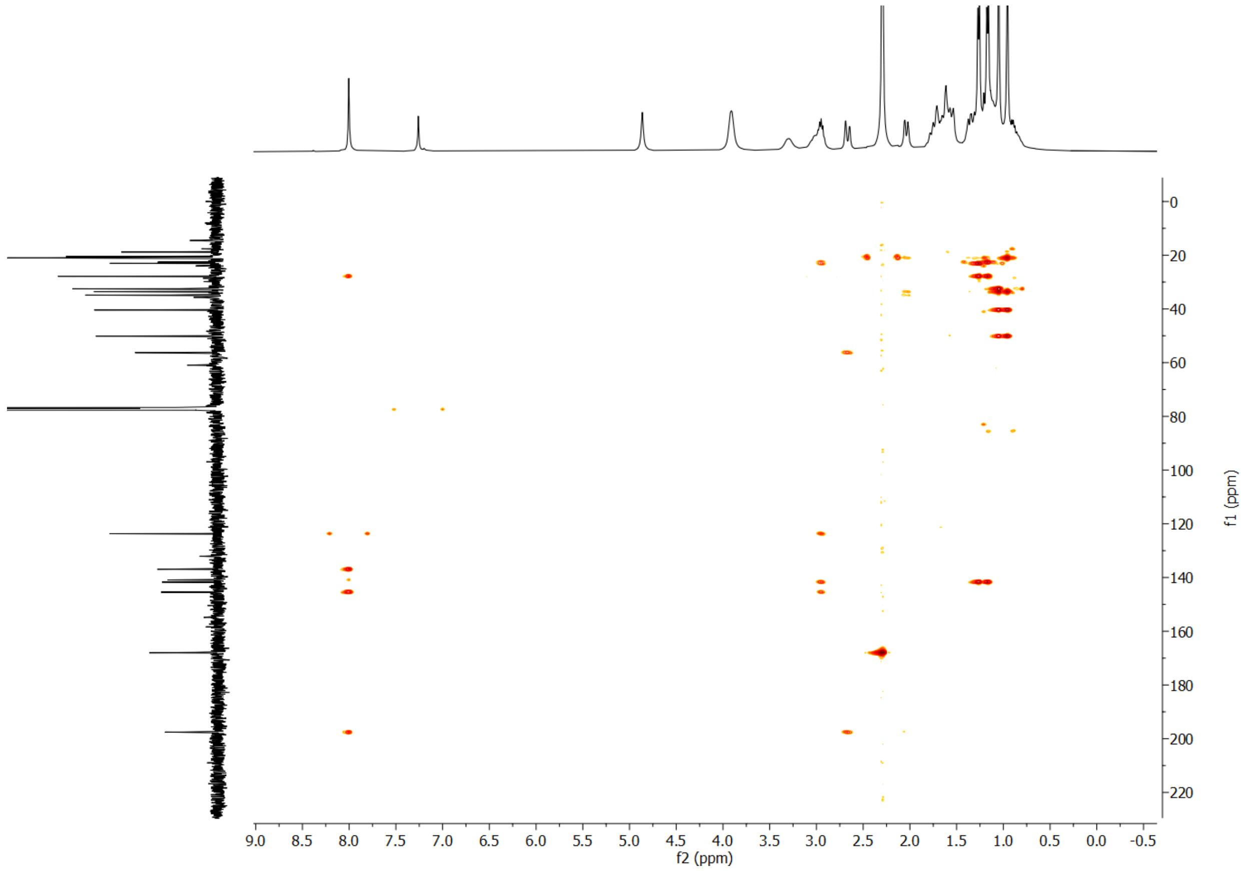

17. Dose-response curves

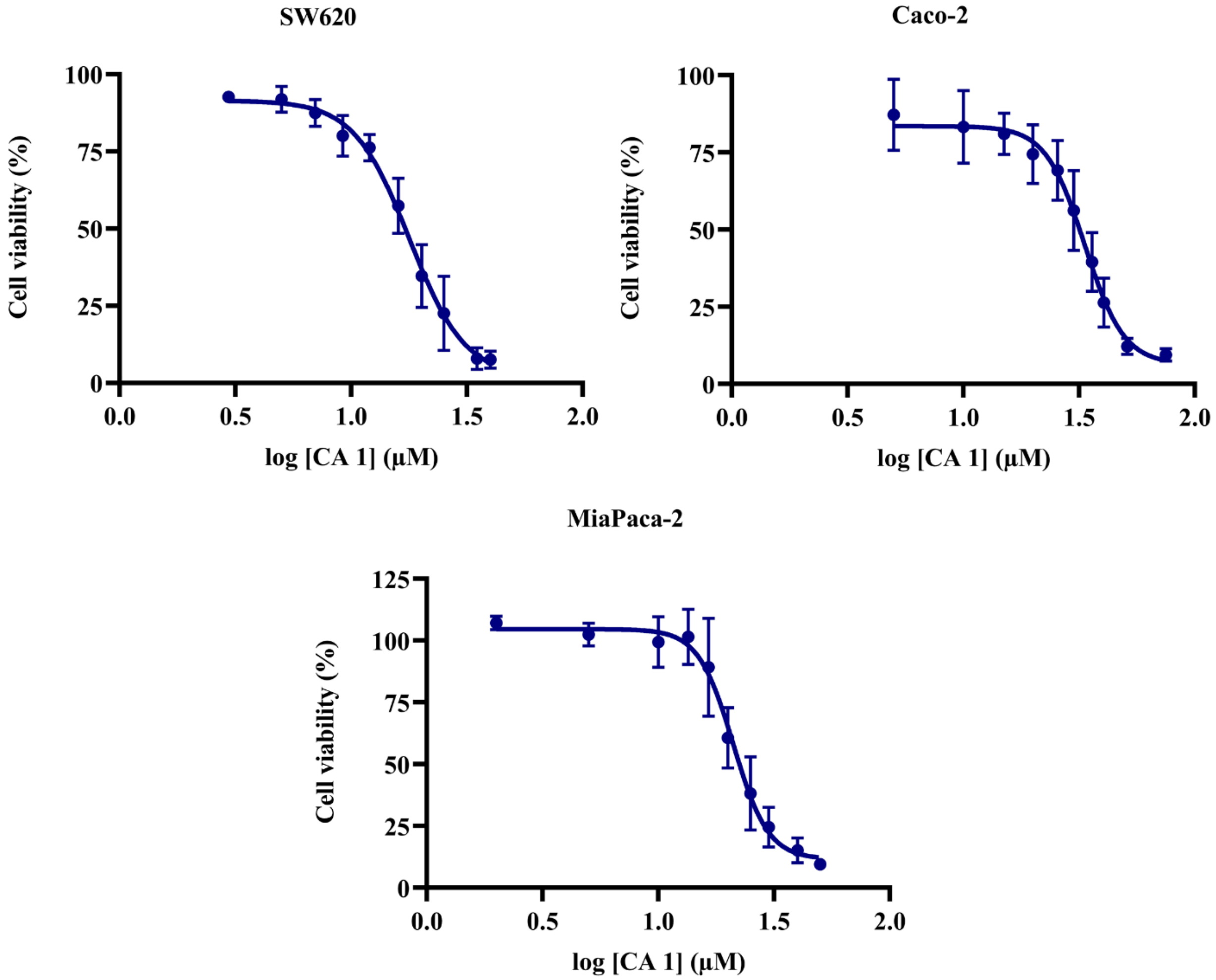

Fig. S1 Dose-response curves of CA **1** in SW620 (IC_{50} = 18 μM), Caco-2 (IC_{50} = 34 μM) and MiaPaca-2 (IC_{50} = 21 μM) cells after 72 h of treatment. Cell viability was determined using the MTT assay, with the results reported as the mean ± SD obtained from at least three independent experiments.

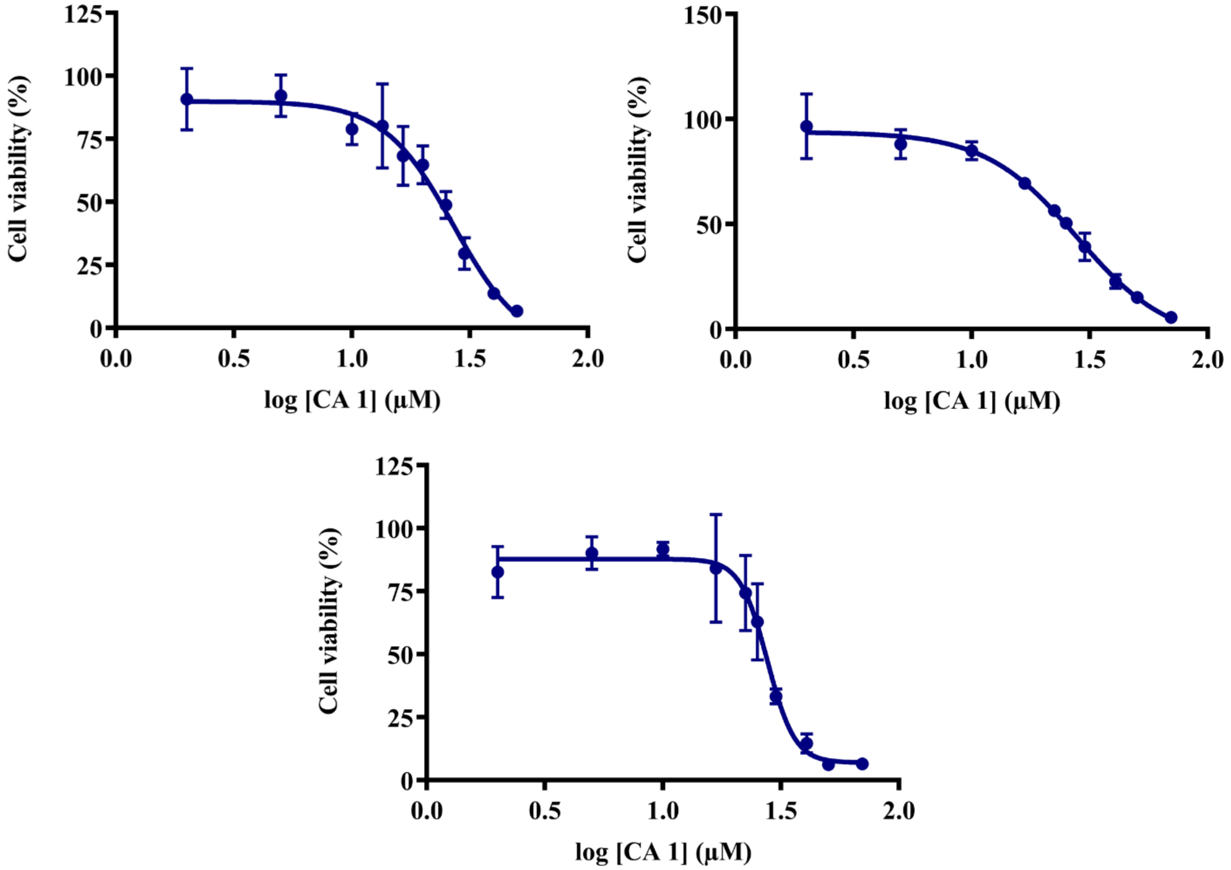

Fig. S2 Dose-response curves of CA **1** in A375 cells after 72 h of treatment. Cell viability was determined using the MTT assay, and the results are reported as mean ± SD. Each curve was obtained from an independent experiment, and the final IC_{50} value (27.6 μM) was calculated as the mean of the IC_{50} values obtained in each independent experiment.

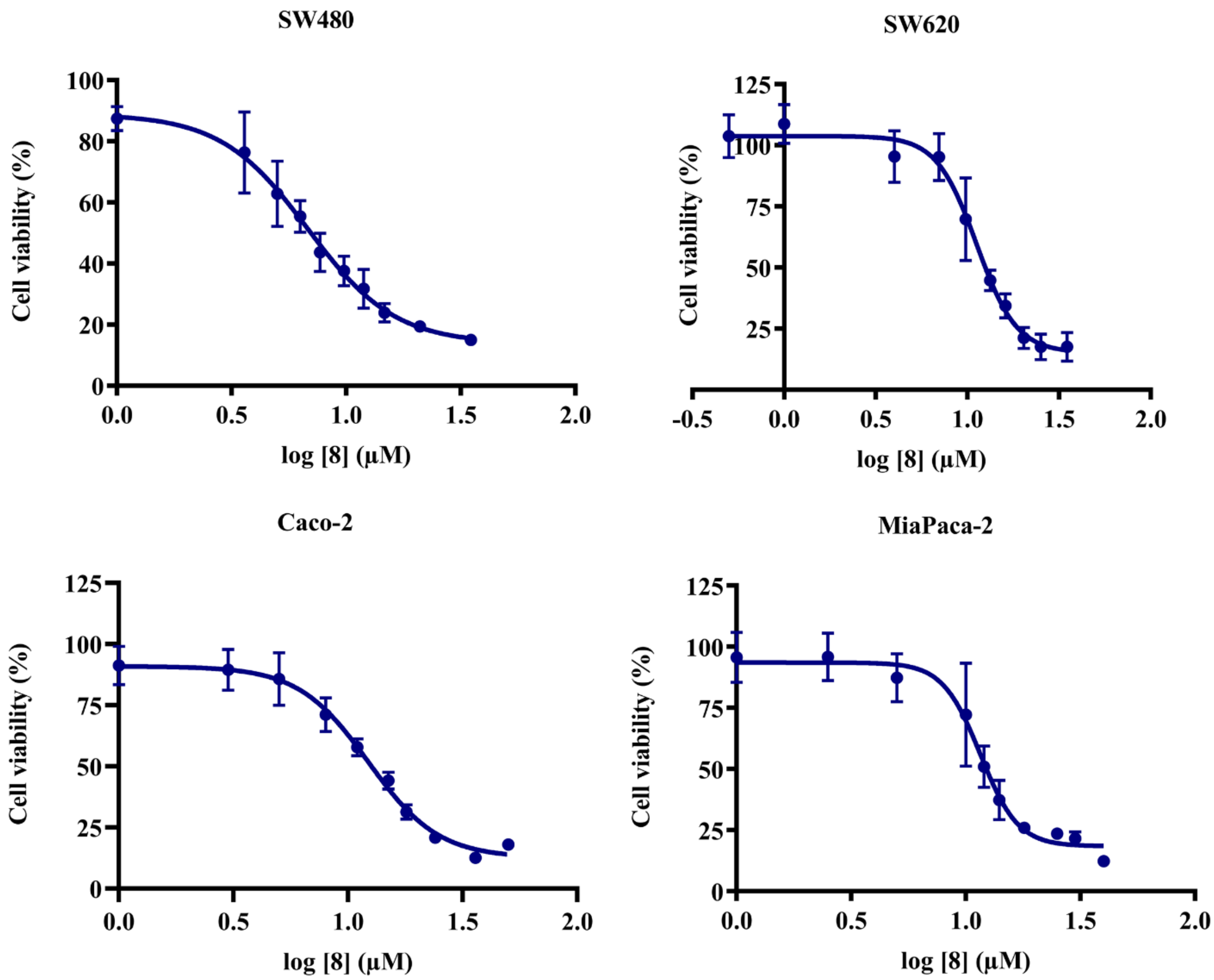

Fig. S3 Dose-response curves of compound **8** in SW480 (IC_{50} = 6.8 µM), SW620 (IC_{50} = 11 µM), Caco-2 (IC_{50} = 12.7 µM) and MiaPaca-2 (IC_{50} = 12 µM) cells after 72 h of treatment. Cell viability was determined using the MTT assay, with the results reported as the mean ± SD obtained from at least three independent experiments.

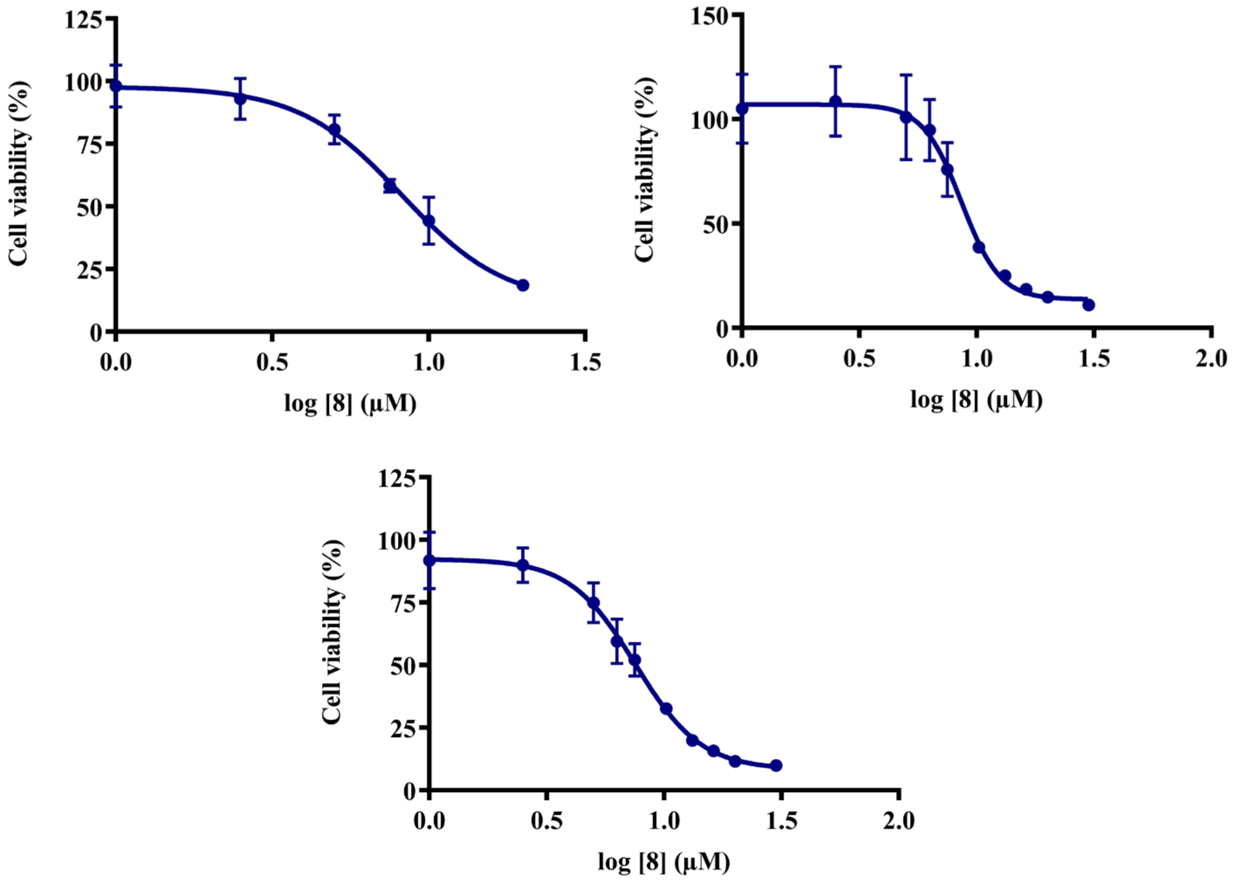

Fig. S4 Dose-response curves of compound **8** in A375 cells after 72 h of treatment. Cell viability was determined using the MTT assay, and the results are reported as mean ± SD. Each curve was obtained from an independent experiment, and the final IC_{50} value (8.1 μM) was calculated as the mean of the IC_{50} values obtained in each independent experiment.

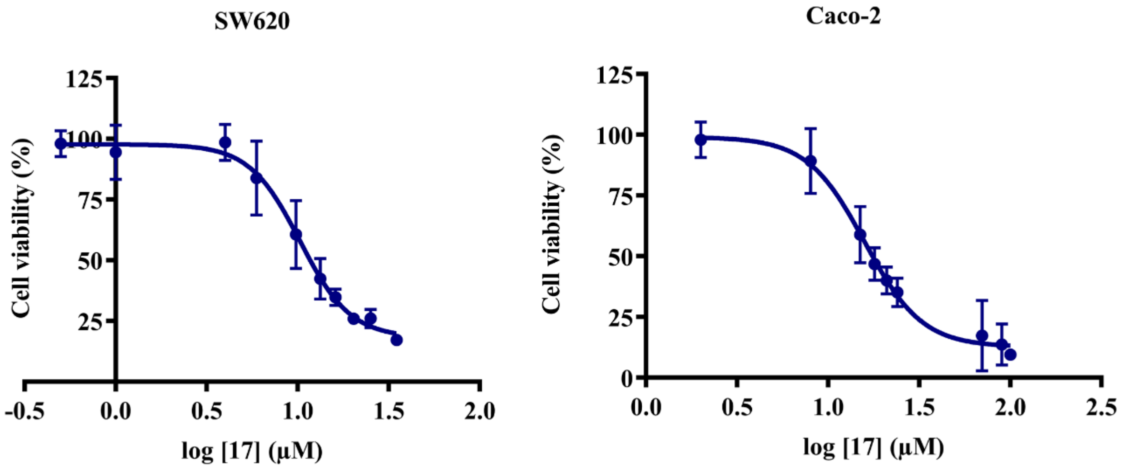

Fig. S5 Dose-response curves of compound **17** in SW620 (IC_{50} = 10 μM) and Caco-2 (IC_{50} = 16 μM) cells after 72 h of treatment. Cell viability was determined using the MTT assay, with the results reported as the mean ± SD obtained from at least three independent experiments.

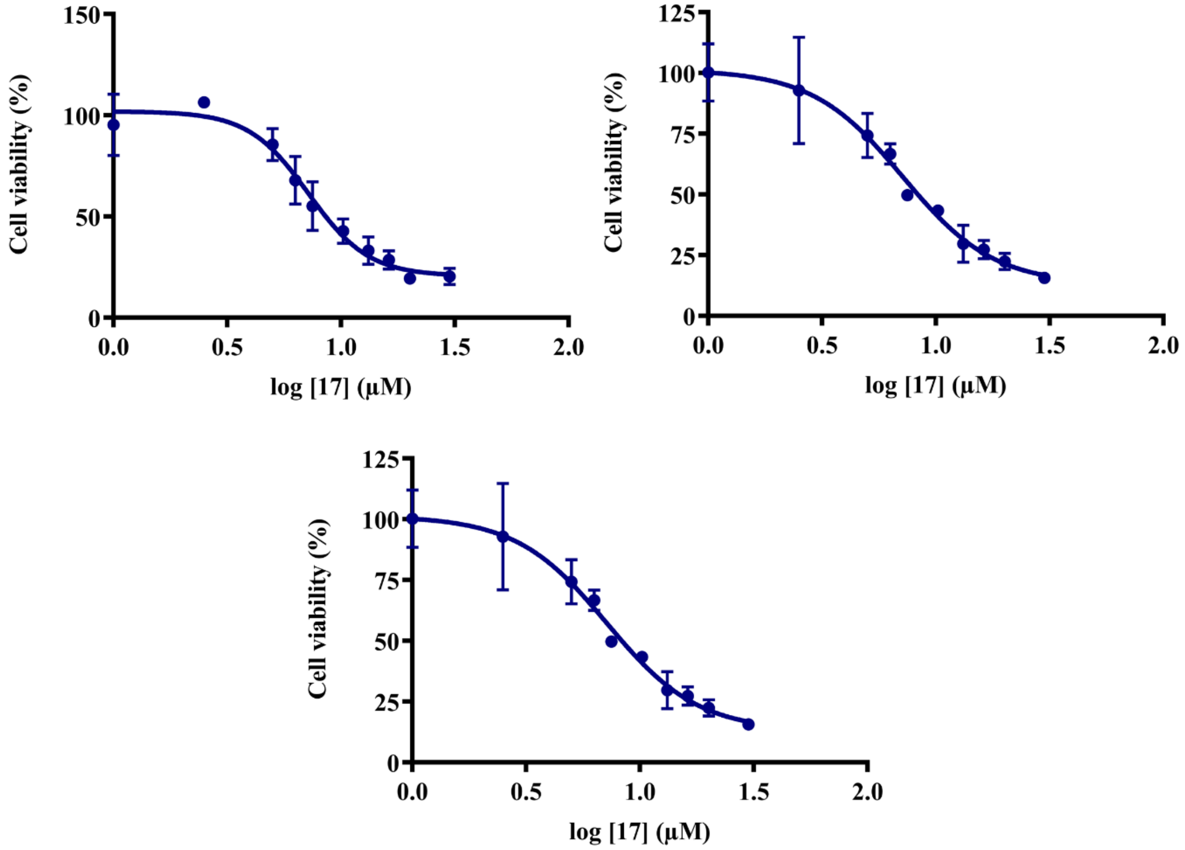

Fig. S6 Dose-response curves of compound **17** in MiaPaca-2 cells after 72 h of treatment. Cell viability was determined using the MTT assay, and the results are reported as mean ± SD. Each curve was obtained from an independent experiment, and the final IC_{50} value (7.5 μM) was calculated as the mean of the IC_{50} values obtained in each independent experiment.

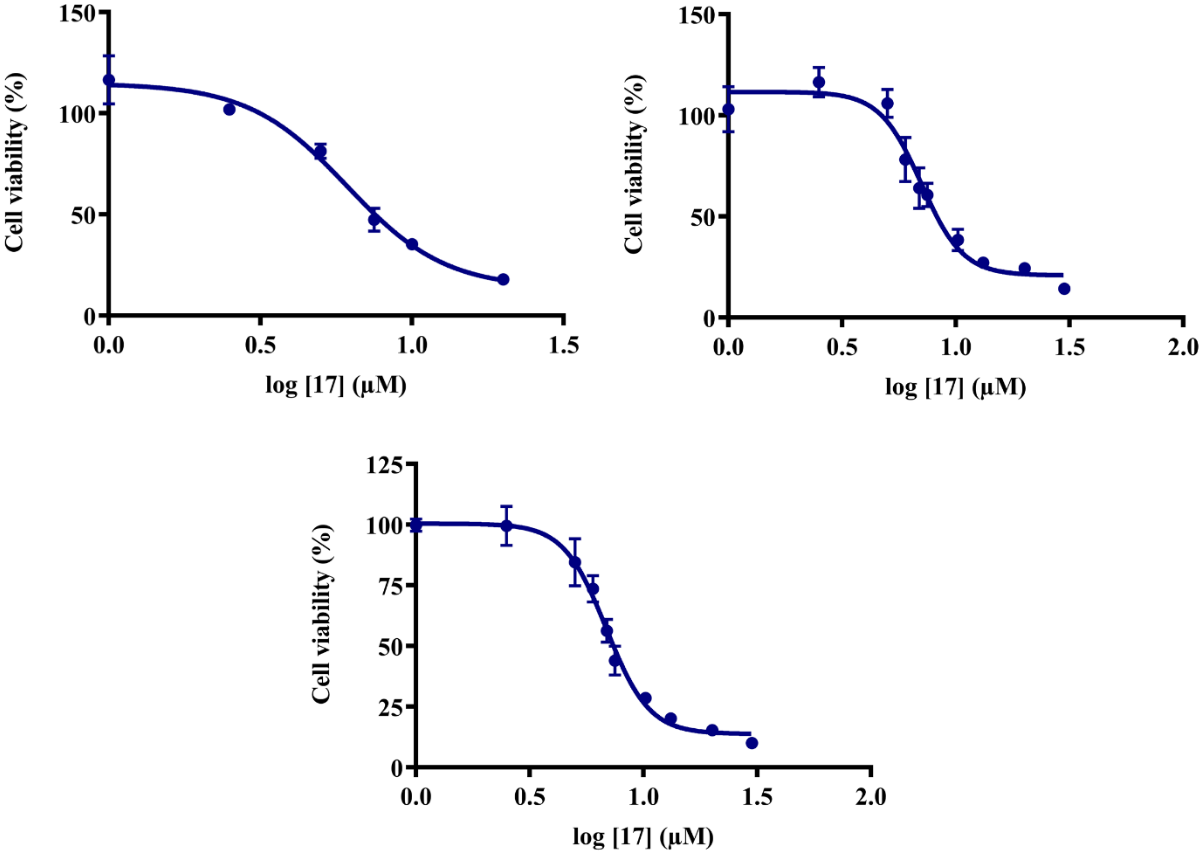

Fig. S7 Dose-response curves of compound **17** in A375 cells after 72 h of treatment. Cell viability was determined using the MTT assay, and the results are reported as mean ± SD. Each curve was obtained from an independent experiment, and the final IC_{50} value (6.7 μM) was calculated as the mean of the IC_{50} values obtained in each independent experiment.

18. Molecular docking studies

Table S1 Key molecular interactions between the CDK6 protein (PDB ID: 6OQL) and the top-ranked docking pose of compound **17**. Distances were measured between the heavy atoms directly involved in each interaction.

Residues	Type of interaction	Distance (Å)
ILE19	Hydrogen bond	3.6
ILE19	Hydrophobic	3.9
VAL27	Hydrophobic	3.9
HIS100	Hydrogen bond (weak)	3.7
VAL101	Hydrogen bond (weak)	3.3
LEU152	Hydrophobic	3.9

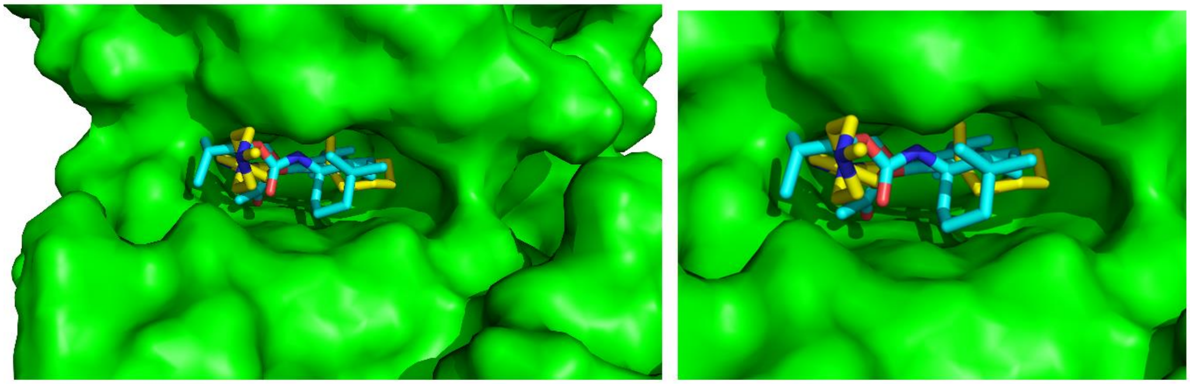

Fig. S8 Structural superposition of compound **17** (cyan) and native ligand in the 6OQL structure (yellow) within the CDK6 binding site, with the CDK6 surface highlighted in green.
